# K_2_[Ir(OH)_6_]: an isolable platform intermediate for the refining of complex chloroiridate mixtures to synthetically useful Ir(iii) coordination complexes

**DOI:** 10.1039/d6sc05573g

**Published:** 2026-07-27

**Authors:** Rebecca M. Willans, Jack H. Heaton, Kristof M. Altus, Simon K. Beaumont, Alison J. Edwards, Michael A. Hayward, Ben Freeman, Vlado K. Lazarov, Nicoleta M. Muresan, David Thompsett, Jackie A. Mosely, Andrew S. Weller, Charlotte E. Willans

**Affiliations:** a Department of Chemistry, University of York York YO10 5DD UK; b Department of Chemistry, Durham University South Road Durham DH1 3LE UK; c Australian Centre for Neutron Scattering, Australian Nuclear Science and Technology Organization Lucas Heights New South Wales 2234 Australia; d Department of Chemistry, Inorganic Chemistry Laboratory, University of Oxford South Parks Road Oxford OX1 3QR UK; e York JEOL Nanocentre, University of York Helix House, York Science Park, Heslington York YO10 5BR UK; f Johnson Matthey Technology Centre Blounts Court Road, Sonning Common Reading RG4 9NH UK

## Abstract

A holistic approach to iridium recycling is the synthesis of technologically useful iridium(iii) coordination complexes from complex aqueous waste streams. Simple, chloride-free complexes such as Ir(acac)_3_ are precursors for a variety of important applications: as homogeneous pre-catalysts, precursors to IrO_*x*_ nanoparticles, and molecular sources for iridium thin films. However, their synthesis from the major refinery intermediate [NH_4_]_2_[IrCl_6_] is typified by harsh reaction conditions and moderate yields. We now show that a key platform intermediate for such complexes, K_2_[Ir(OH)_6_], can be reliably isolated as a crystalline, compositionally pure, material from both [IrCl_6_]^2−^ salt precursors and crude refinery chloroiridate solutions, as optimized using the convenient analytical reporters of cyclic voltammetry and ultraviolet-visible spectroscopy. K_2_[Ir(OH)_6_] is shown to be a platform intermediate for the formation of iridium(iii) coordination complexes, demonstrated by mild, selective and high yielding routes to known Ir(acac)_3_, *cis*- and *trans*-K[Ir(oxalate)_2_(H_2_O)_2_], and new K_3_[Ir(citrate)_2_]. The reliable isolation of K_2_[Ir(OH)_6_] from refinery streams opens up opportunities for iridium recycling by the synthesis of industrially important, but compositionally simple, Ir(iii) complexes.

## Abstract 2

The platform intermediate K_2_[Ir(OH)_6_] is isolated from complex chloroiridate mixtures and can be used to make technologically useful Ir(iii) cooridnation complexes.

## Graphical abstract

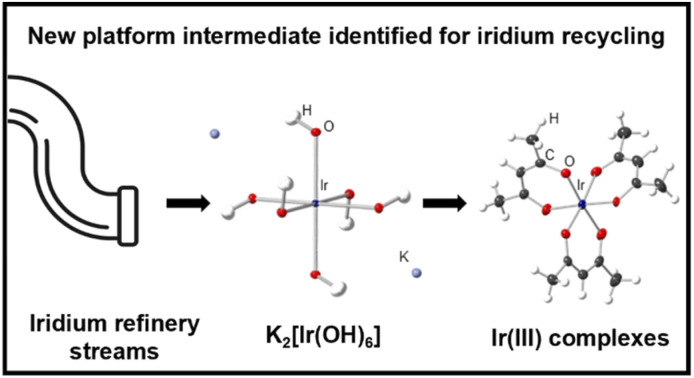

## Introduction

Iridium is one of the scarcest metals, with only 7–8 t per year mined globally from primary sources.^1^ In contrast, it is a technologically essential element given its role in Proton Exchange Membrane (PEM) water electrolysis (IrO_*x*_ nanoparticles), process catalysis (*e.g.* Cativa process), coating and electronic applications,^1^ and Organic Light Emitting Diodes.^2,3^ Simple, halide-free complexes such as Ir(acac)_3_ (acac = acetylacetonato) are widely used precursors for these applications. An attractive approach to iridium recycling would be the synthesis of such technologically useful coordination complexes from secondary, post-use, sources of iridium. Currently, these complex iridium waste streams are refined to a crude chloroiridate solution in concentrated hydrochloric acid.^4,5^ Using multi-step purification processes,^6^ synthetic intermediates [NH_4_]_2_[IrCl_6_] or iridium sponge can then be isolated from such solutions and used as precursors for subsequent manufacturing. However, [NH_4_]_2_[IrCl_6_] and iridium sponge both have disadvantages as intermediates for this process. While synthetic routes to simple coordination complexes from salts of [IrCl_6_]^2−^ are widely reported, they are typified by moderate yields and harsh conditions.^7^ Furthermore, the presence of halides as impurities in the target complexes, even in trace quantities, can have an inhibitory effect on performance in the final application.^8^ Iridium sponge, whilst halide-free, requires an alkaline fusion process at temperatures above 500 °C to form soluble species suitable for further synthesis.^9^ Given the importance of recycling iridium waste streams, and the challenges associated with the current methods, a soluble halide-free platform intermediate that sits between the crude refinery chloroiridate solutions and simple, chloride-free, iridium precursors is highly desirable.

Suitable intermediate complexes would be simple homoleptic iridium complexes with aqua- or hydroxy ligands. Whilst the formation of the hexa-aqua Ir(iii) complex [Ir(OH_2_)_6_][ClO_4_]_3_ in acidic aqueous solution has been demonstrated using ultraviolet-visible (UV-vis) spectroscopy,^10^ this complex has not been isolated. An alternative is insoluble ‘IrO_2_·*n*H_2_O’, frequently referred to as ‘hydrated iridium oxide’ or ‘iridium hydroxide’, which is of debated speciation.^11,12^ However, harsh redox reagents, such as hydrazine, or hydrothermal conditions (200 °C, 20 M NaOH), are required to generate soluble species from IrO_2_·*n*H_2_O.^12^

The homoleptic Ir(iv) hexahydroxide complexes M_2_[Ir(OH)_6_] [M = *e.g.* K^+^, Na^+^, H^+^] present more viable options as platform intermediates. Several hexahydroxy Platinum Group Metal (PGM) complexes have been isolated,^13–19^ and the analogous platinum species [Pt(OH)_6_]^2−^ is a commercially available precursor from which synthetic routes to simple platinum coordination complexes have been established.^20,21^ This demonstrates the potential suitability of this class of complex for subsequent, industrially relevant, synthetic transformations.

While ill-defined hydroxyiridate solutions containing mixtures of species including [Ir(OH)_6_]^2−^ are readily formed from chloroiridate precursors by the cleavage of Ir–Cl bonds under basic conditions and have been widely reported,^12,22–27^ isolated M_2_[Ir(OH)_6_] complexes are less common. The first reported salt of this kind was K_2_[Ir(OH)_6_], synthesised from Na_2_[IrCl_6_] under basic conditions *via* a complex and poorly defined multi-step protocol.^28^ A series of insoluble ‘M[Ir(OH)_6_]’ salts (M = Zn^2+^, Cd^2+^ and Ca^2+^) have also been isolated *via* precipitation from solutions of K_2_[IrCl_6_] adjusted to pH 12.^29,30^ Detailed structural characterisation was not reported, leaving ambiguity as to their exact molecular structure. Finally, crystallographically characterised Na_2_[Ir(OH)_6_] and Ba_2_[Ir(OH)_6_][OH]_2_ have been synthesised using energy-intensive hydrothermal conditions from IrCl_3_·3H_2_O and [NH_4_]_2_[IrCl_6_], respectively.^31,32^ Aside from the thermal dehydration of Na_2_[Ir(OH)_6_] to Na_2_[IrO_3_] at 220 °C,^31^ the subsequent reactivity of well-defined [Ir(OH)_6_]^2−^ species remains, to date, unexplored. A potentially scalable, solution-based route to an [Ir(OH)_6_]^2−^ salt that produces compositionally pure material would open up opportunities for the development of synthetic routes from this simple and useful iridium(iv) hydroxide.

**Scheme 1:**
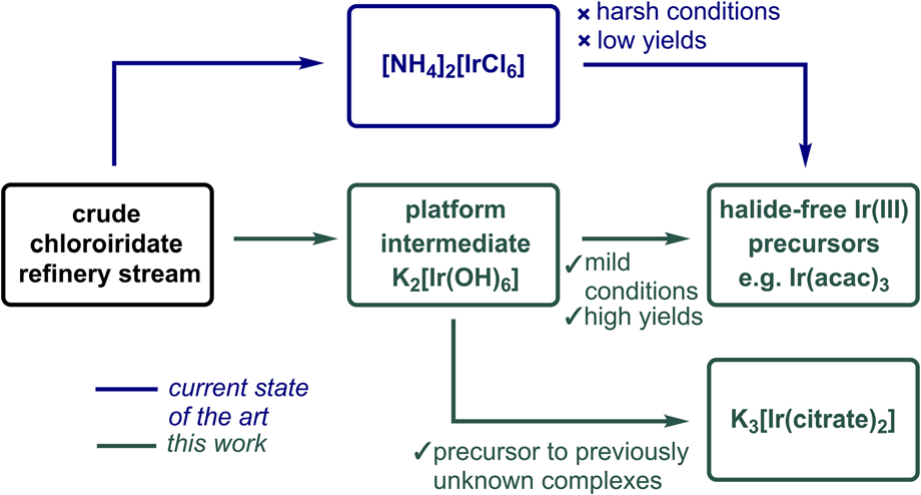
The synthesis of late-stage Ir(iii) coordination complexes from the halide-free platform intermediate K_2_[Ir(OH)_6_] isolated from a crude chloroiridate mixture.

In this contribution, we present K_2_[Ir(OH)_6_] as an isolable, chloride-free intermediate that is placed between complex post-refinery chloroiridate solutions and synthetically useful Ir(iii) coordination complexes (Scheme 1). We show that analytically pure, fully characterised, K_2_[Ir(OH)_6_] can be synthesised using a KOH/heating protocol in aqueous solution, which is developed through detailed speciation studies. Subsequent mild, selective, and high-yielding routes to Ir(acac)_3_, K[Ir(C_2_O_4_)_2_(H_2_O)_2_] (oxalate) and the previously unknown citrate complex K_3_[Ir(C_6_H_5_O_7_)_2_] are then reported. With the identification of reliable analytical handles for the synthesis of K_2_[Ir(OH)_6_], our simple methodology also has the potential to be scaled, offering a new method to refine chloroiridate solutions into pure, technologically useful, halide-free precursors.

This work complements, and contrasts with, the recent report by Williams and co-workers,^12^ in which the Ir(i) chloride precursor [(1,5-COD)IrCl]_2_ was isolated from organoiridium laboratory waste *via* treatment of ‘IrO_2_·*n*H_2_O’ with hydrazine.

## Results and discussion

### The complexity of the chloroiridate refinery stream demonstrated by nano-electrospray ionisation mass spectrometry (nESI-MS)

The aqueous speciation of chloroiridate solutions, even from clean precursor salts, is known to be complex.^33–35^ Iridium species in aqueous solution are prone to changes in oxidation state and coordination environment *via* aquation or hydroxylation, as determined by variables such as chloride concentration and pH.^33–37^ Identifying the species present in the resulting mixtures using readily available analytical techniques is challenging.^27,37^

**Fig. 1:**
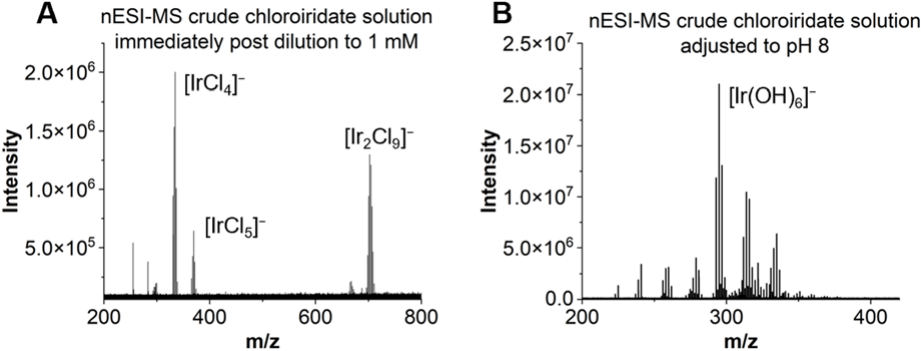
nESI-MS spectra (negative mode) of crude chloroiridate solution diluted to 1 mM [Ir], obtained using a SolariX FT-ICR mass spectrometer using an in-house offline nESI source with an API voltage of 1000 V. (A) Spectrum obtained immediately post dilution (10 minutes). (B) Spectrum obtained one hour post-basification to pH 8 with aqueous ammonia.

In order to provide a report on the complexity of the crude chloroiridate solutions, these were analysed by mass spectrometry using nano-electrospray ionisation, nESI (Fig. 1). nESI-MS enables the direct infusion of high concentration analytes,^38^ including acidic, metal-containing solutions, and uses subsequent stages of the mass spectrometer to reduce the high ion count to appropriate levels. The significantly reduced flow rate of nESI-MS means that other ion source parameters can be moderated, making for a gentler ionisation process. It must still be noted, however, that effects such as fragmentation, in-source dimerisation and oxidation state changes during ionisation mean that the gas phase is not always directly representative of speciation in solution.^39,40^

A crude refinery chloroiridate solution provided by Johnson Matthey was diluted from 21.85 wt% [Ir] to the analytically suitable concentration of 1 mM [Ir]. Under the assumption that equilibration is slow relative to the time frame of the analytical technique, a mass spectrum obtained immediately post dilution (10 minutes) (Fig. 1A) is considered representative of the speciation prior to dilution. Whilst [IrCl_6_]^2−^ is not directly observed due to the instability of doubly charged anions in the gas phase, [IrCl_5_]^−^ and [IrCl_4_]^−^ ions are confirmed by tandem mass spectrometry (MS^2^) experiments as the fragmentation products of [IrCl_6_]^2−^ (Fig. S8 and S9). Dimeric [Ir_2_Cl_9_]^−^ is also observed, attributed to an in-source dimerisation – as commonly observed by mass spectrometry for relatively high concentration samples.^39^ nESI-MS analysis is thus consistent with [IrCl_6_]^2−^ being the predominant species in the refinery chloroiridate solution.

Spectra obtained by nESI-MS of the chloroiridate refinery solution under different conditions show the complexity of speciation arising from these solutions (*e.g.* Fig. S5).^41^ As an example, the effect of changing pH is demonstrated over an experimentally relevant timeframe (Fig. 1B). The crude chloroiridate solution was diluted to 1 mM [Ir], basified with aqueous ammonia and analyzed by nESI-MS after one hour. Under these conditions, cleavage of the Ir–Cl bonds is observed, resulting in a complex mixture of monomeric species of aqua- and hydroxy-coordinated complexes.

With the complexity and variation of speciation within the chloroiridate refinery solution arising from changing conditions established, the synthesis of well-defined, stable K_2_[Ir(OH)_6_] from these mixtures is described next. This offers a simple starting point from which to synthesize late-stage iridium(iii) precursors.

### The development of a repeatable synthetic protocol for the formation of K_2_[Ir(OH)_6_] from Na_2_[IrCl_6_] and crude refinery mixtures using speciation insights from cyclic voltammetry and UV-vis spectroscopy

To enable the development of a synthetic protocol for the formation of K_2_[Ir(OH)_6_] from crude chloroiridate solutions, a repeatable procedure from the clean precursor salt Na_2_[IrCl_6_] was first established.

The synthesis of K_2_[Ir(OH)_6_] from Na_2_[IrCl_6_] *via* a multi-step process under basic conditions was first reported in 1973.^28^ In our hands, replication of this procedure did not result in the formation of the desired product, either as crystalline K_2_[Ir(OH)_6_] or as a species in solution as determined using cyclic voltammetry (CV, *vide supra*, Fig. S30). However, an adaptation of this procedure, optimized using CV and UV-vis spectroscopy, results in a repeatable 60% yield of K_2_[Ir(OH)_6_] from the precursor salt Na_2_[IrCl_6_]. Furthermore, K_2_[Ir(OH)_6_] can be isolated from a crude refinery chloroiridate solution in a 35% yield by a simple modification.

The developed protocol takes the form of three discrete steps. Na_2_[IrCl_6_] (0.5 g, 1.1 mmol) is first dissolved in KOH solution (2 M, 50 mL) with H_2_O_2_ (35% solution, 0.5 mL) and heated to 130 °C for 15 minutes (heating step 1). The resulting solution is left to sit at room temperature for three hours (three hour sit). Subsequently, this solution is heated to 110 °C in air until its volume halves (heating step 2) and left to slowly concentrate at room temperature over 36 hours. This sequence results in the isolation of red prismatic crystals of K_2_[Ir(OH)_6_] (0.25 g, 60%).

The challenge in identifying and optimizing a repeatable synthetic protocol for the formation of K_2_[Ir(OH)_6_] lay in the difficulty of distinguishing individual iridium species in these basic solutions.^42^ The specificity of the described procedure is such that changes in any one variable, including length of the resting period, temperature (±10 °C), or the rate of evaporation, result in the failure to isolate K_2_[Ir(OH)_6_] (Tables S3/S4). This presents a challenge both in developing a repeatable synthetic protocol and in enabling scale-up, which we have addressed through establishing a simple and reliable analytical handle to assess, and thus stage-gate, each reaction step.

**Fig. 2:**
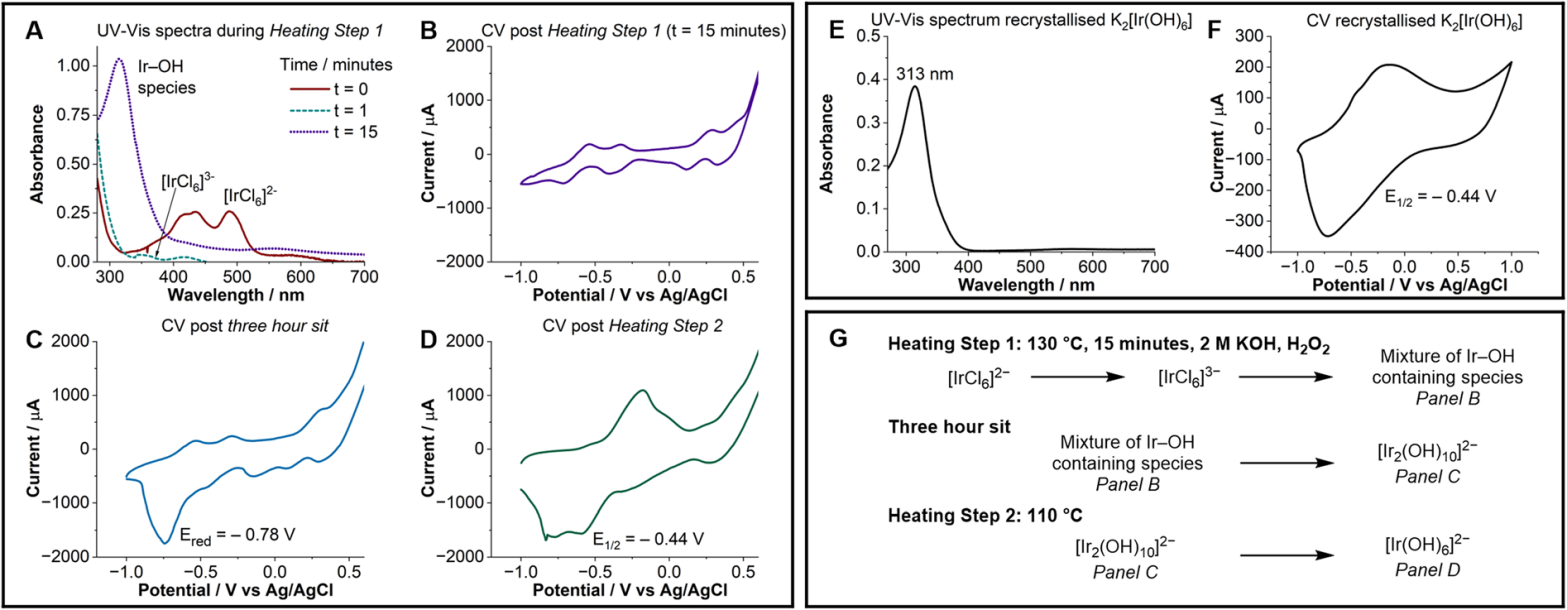
Reaction monitoring of the synthesis of K_2_[Ir(OH)_6_] from Na_2_[IrCl_6_] using UV-vis spectroscopy and CV. CV data are plotted in the IUPAC convention, with more positive potentials to the right and the anodic current upwards. (A) UV-vis spectra of aliquots of the crude reaction mixture during heating step 1, showing reduction of Ir(iv) to Ir(iii) followed by a hydroxylation process. 10 µL (*t* = 0, *t* = 15) or 100 µL (*t* = 1) aliquots were taken and quenched by rapid cooling and dilution in 0.3 mL of 2 M KOH. Note the tenfold concentration of the *t* = 1 spectrum to enable visualisation given the relative extinction coefficients of the Ir(iv) and Ir(iii) species. (B) CV post heating step 1 showing a mixture of Ir–OH containing species present. (C) CV post three hour sit showing solution speciation tending to a major species with an irreversible reduction event at −0.78 V *vs.* Ag/AgCl. (D) CV post heating step 2, prior to separation of the crystalline product, showing the characteristic trace of [Ir(OH)_6_]^2−^ in solution. (E) UV-vis spectrum of recrystallized K_2_[Ir(OH)_6_] in 2 M KOH (*ε* = 2500 M^−1^ cm^−1^) showing a characteristic Ir–O LMCT band at 313 nm. (F) CV of recrystallized K_2_[Ir(OH)_6_] in 2 M KOH with redox potential *E*_1/2_ = −0.44 V *vs.* Ag/AgCl. (G) Scheme showing proposed species accounting for observed data during each key reaction stage.

Data from CV and UV-vis spectroscopy enabled the identification of key speciation features during the synthesis of K_2_[Ir(OH)_6_] from Na_2_[IrCl_6_] (Fig. 2). Full reduction from Ir(iv) to Ir(iii) is observed by UV-vis spectroscopy within the first five minutes of heating step 1. The intense Ligand-to-Metal Charge Transfer (LMCT) bands of low-spin d^5^ Ir(iv) [IrCl_6_]^2−^ (*e.g.* 489 nm, *ε* = 3500 M^−1^ cm^−1^) are quickly (1 minute) replaced by the lower intensity, Laporte-forbidden d–d transitions of low-spin d^6^ Ir(iii) [IrCl_6_]^3−^ (*e.g.* 415 nm, *ε* = 120 M^−1^ cm^−1^). Complete conversion to Ir–OH containing species, with a characteristic LMCT band at 313 nm,^10,23–25,43^ occurs within 15 minutes (Fig. 2A). Aliquots from this point onwards in the protocol all show a broad absorbance band at 313 nm (Fig. S31).

UV-vis spectroscopy is therefore unsuitable for distinguishing between mixtures of Ir–OH containing species. Subsequent steps of the synthetic protocol were thus investigated using cyclic voltammetry enabled by single-use Screen Printed Electrodes (SPEs). These require only small volumes of analyte solution (0.1 mL), enabling reaction monitoring through the sampling of aliquots. Analysis by cyclic voltammetry of Ir–OH containing species is challenging using traditional electrode set-ups due to the loss of resolution arising from the electrodeposition of iridium oxide onto the electrode surface upon repeated potential scans.^44^ CV analysis using SPEs demonstrates that the broad UV-vis absorbance band at 313 nm corresponds to multiple iridium species in solution at the end of heating step 1 (Fig. 2A and B).

The mixture of species formed post heating step 1 evolves to a major species after a three hour sit, which exhibits an irreversible reduction at −0.78 V *vs.* Ag/AgCl (Fig. 2C). By comparison to the electrochemical behaviour of independently synthesised [NBu_4_]_2_[Ir_2_Cl_10_],^45^ it is postulated that this species is likely a bridged dimer such as [Ir_2_(OH)_10_]^2−^.^46^ Cyclic voltammetry of both [NBu_4_]_2_[Ir_2_Cl_10_] (+0.45 V *vs.* Ag/AgCl) and the crude reaction solution post three hour sit (–0.78 V *vs.* Ag/AgCl) shows a characteristic broad irreversible reduction at a potential of approximately 0.3 V below that of the equivalent monomeric species, *i.e.*, [IrCl_6_]^2−^ (+0.74 V *vs.* Ag/AgCl) and [Ir(OH)_6_]^2−^ (*vide supra*, −0.44 V *vs.* Ag/AgCl), respectively (Fig. S33).

Finally, the conversion of the bulk of the crude solution into [Ir(OH)_6_]^2−^ through concentration in heating step 2 was determined. A new species was observed in the reaction solution, containing a major redox event at −0.44 V (Fig. 2D). Concentration *via* the slow evaporation of water resulted in the formation of crystalline K_2_[Ir(OH)_6_] with a 60% isolated yield (250 mg scale). The cyclic voltammogram of the isolated material (2 M KOH) shows a quasi-reversible event at −0.44 V *vs.* Ag/AgCl (Fig. 2F). A broad and intense LMCT absorbance, typical of Ir–OH containing species, is observed at 313 nm by UV-vis spectroscopy. The large extinction coefficient (*ε* = 2500 M^−1^ cm^−1^) is indicative of a charge transfer band; d–d bands are not observed (Fig. 2E). The remaining reaction solution post removal of crystalline K_2_[Ir(OH)_6_] shows a complex mixture of iridium species as analysed by cyclic voltammetry (Fig. S32).

An adaptation of this protocol enables the isolation of K_2_[Ir(OH)_6_] in a 35% yield from a crude, post-refinery, chloroiridate solution. An initial neutralisation step is required, following which the total solution is made up to a 2 M concentration of KOH. From this point, the same procedure is followed, resulting in the isolation of crystalline K_2_[Ir(OH)_6_] that is indistinguishable analytically from materials produced from Na_2_[IrCl_6_].

Thus, a repeatable protocol for the synthesis of K_2_[Ir(OH)_6_] has been established, from both the clean precursor salt Na_2_[IrCl_6_] and crude refinery chloroiridate solutions, using a reliable analytical reaction handle that can be deployed for *in situ* reaction monitoring and synthetic stage-gating. The isolation of crystalline K_2_[Ir(OH)_6_] now enables the full and detailed characterization of this complex to be conducted, as described next.

### The isolation of K_2_[Ir(OH)_6_] as single crystals on a 250 mg scale and structural characterisation in the solid state using X-ray diffraction, single-crystal neutron diffraction, vibrational spectroscopy and magnetometry

**Fig. 3:**
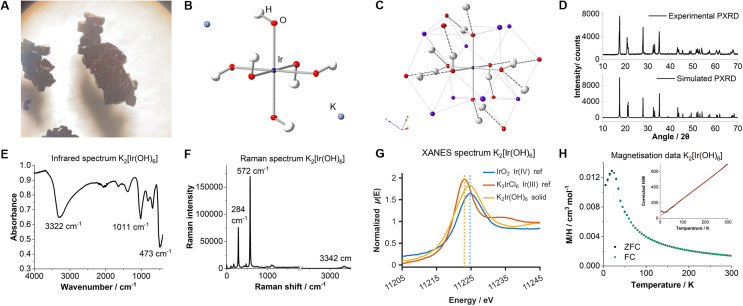
The structural characterisation of K_2_[Ir(OH)_6_]. (A) Photograph of red prismatic crystals of K_2_[Ir(OH)_6_]. (B) Formula unit of K_2_[Ir(OH)_6_] as determined by single-crystal neutron diffraction. Selected bond lengths (Å): O1–H1 0.9744 (7) and Ir1–O1: 2.016 (4) and angles (°): Ir1–O1–H1 108.75 (5), Ir–O: 2.016 (4), and O–Ir–O 90.53 (17). (C) Packing diagram generated from the neutron diffraction data, highlighting O–H⋯O and K^+^···O interactions. (D) Powder XRD (PXRD) pattern of the bulk crystalline material and pattern simulated from the SC-XRD structure of K_2_[Ir(OH)_6_]. (E) Infrared spectrum of K_2_[Ir(OH)_6_]. (F) Raman spectrum of K_2_[Ir(OH)_6_]. (G) Ir L_3_-edge X-ray absorption spectrum of solid K_2_[Ir(OH)_6_] in the XANES (X-ray absorption near edge structure) region shown in comparison to K_3_[IrCl_6_] and IrO_2_ reference compounds for Ir(iii) and Ir(iv), respectively. The vertical dashed lines are a guide to the eye for the white line peak maximum of the two reference compounds Ir(iii) orange and Ir(iv) blue. (H) Zero-field-cooled and field-cooled magnetisation data collected for K_2_[Ir(OH)_6_] in an applied field of 10 000 Oe. Inset shows fit to the Curie–Weiss Law in the range 70 < *T*/*K* < 300 to yield values of *C* = 0.450(3) cm^3^ K mol^−1^, *θ* = −11.9(4) K, and *K* = −4.11(6) × 10^−5^ cm^3^ mol^−1^.

Crystals of K_2_[Ir(OH)_6_] suitable for single-crystal X-ray diffraction (SC-XRD) were isolated through the slow evaporation of water over 36 hours from the reaction mixture post heating step 2 of the K_2_[Ir(OH)_6_] synthetic protocol. The resulting red prismatic crystals (Fig. 3A) were stable in air for approximately one week; however, over extended periods a colour change to blue was observed. The formation of iridium oxide under these conditions was demonstrated by infrared spectroscopy and mass spectrometry (Fig. S34/S35). K_2_[Ir(OH)_6_] was therefore stored under an atmosphere of nitrogen. No colour change was observed under these conditions upon storage for several months.

Whilst single-crystal precession and powder diffraction data for K_2_[Ir(OH)_6_] have previously been reported, the unambiguous molecular structure has not been determined.^47^ SC-XRD data collected at 110 K show that K_2_[Ir(OH)_6_] crystallizes in an *R*3̄ space group (*R*_1_ = 2.02%) (Fig. S58). The iridium centre sits in a special position so that the asymmetric unit consists of Ir–OH and K, with Ir and K at 1/6 and 2/6 occupancies, respectively. The Ir–O bond length is 2.016(4) Å and the IrO_6_ core is rigorously octahedral. The powder X-ray diffraction (PXRD) pattern of the material generated from recrystallisation closely matches the powder pattern simulated from the SC-XRD data, confirming the identity of the bulk as K_2_[Ir(OH)_6_] (Fig. 3D). Precise determination of the position of the hydrogen atoms was enabled by single-crystal neutron diffraction. Single crystals (2 mm × 2 mm × 1 mm) were studied by Laue neutron diffraction at 260 K using the KOALA 2 beamline at the Australian Nuclear Science and Technology Organisation (ANSTO). The structure was refined with no evidence of disorder to give the same heavy atom structure as SC-XRD (Fig. 3B) as well as the precise location of the hydrogen atoms. An O–H distance of 0.9744(7) Å and an Ir–O–H angle of 108.75(5)° were measured. An intermolecular O_D_(H)⋯O_A_ distance of 2.8591(6) Å and an O_D_–H⋯O_A_ angle of 164.40(6)° are indicative of moderate strength hydrogen bonding.^48–50^ When combined with K^+^···O interactions, a high symmetry three-dimensional network thus results from this bonding pattern (Fig. 3C).

The infrared spectrum of K_2_[Ir(OH)_6_] matches datapreviously reported,^28^ with a broad *ν*(O–H) stretch observed at 3322 cm^−1^ and two strong bands at 1011 cm^−1^ and 479 cm^−1^, assigned to a *δ*(O–H) bend and a *ν*(Ir–O) stretch, respectively (Fig. 3E).^51,52^ K_2_[Ir(OH)_6_] shows two major Raman shifts at 284 cm^–1^ and 572 cm^−1^ (Fig. 3F). These fall in the expected ranges for an Ir–O bend and an Ir–O stretch, respectively.^53^

Ir L_3_-edge X-ray Absorption Spectroscopy (XAS) (Fig. 3G) confirms the oxidation state of solid K_2_[Ir(OH)_6_], showing a shape and white line position closely aligned with those of the Ir(iv) reference compound (IrO_2_), with a white line centred at 11225 eV. Fig. S42 shows the derivative spectrum and the zero crossing point. It has been noted that the white line position and intensity are particularly sensitive to the oxidation state, and the overall line shape is similar to that of both IrO_2_ and other Ir(iv) complexes.^54^ The Ir(iii) reference complex, K_3_[IrCl_6_], shows a different line shape and white-line center (∼11223 eV).

K_2_[Ir(OH)_6_] is a low-spin d^5^ species that is expected to exhibit paramagnetic behaviour. The paramagnetic susceptibility of K_2_[Ir(OH)_6_] as a function of temperature was measured using SQUID magnetometry (Superconducting QUantum Interference Device) (Fig. 3H). These data fit the Curie–Weiss Law in the range 70 < *T* < 300 K with values of *C* = 0.450(3) cm^3^ K^−1^ mol^−1^, *θ* = −11.9(4) K and *K* = − 4.11(6) × 10^−5^ cm^3^ mol^−1^. A magnetic moment value of *µ*_eff_^2^ of 3.60 *µ*_B_^2^ (*µ*_eff_ = 1.90 *µ*_B_) was determined. This is larger than expected for a spin-only *s* = ½ system (*µ*_eff_ = 1.7 *µ*_B_), but consistent with the presence of first-order spin–orbit coupling in a low-spin octahedral t^5^_2g_ Ir(iv) system. This magnetic moment corresponds closely to those reported previously for Na_2_[Ir(OH)_6_] (*µ*_eff_ = 1.9 *µ*_B_, vibrating sample magnetometer)^31^ and Cd[Ir(OH)_6_] (*µ*_eff_ = 1.91 *µ*_B_, Cahn microbalance).^30^ Below 70 K, data are consistent with inter-molecular magnetic coupling.

### Differential temporal speciation of K_2_[Ir(OH)_6_] in non-buffered aqueous solution in air and under argon

With pure K_2_[Ir(OH)_6_] in hand, the behaviour of this complex in aqueous solution was investigated. K_2_[Ir(OH)_6_] is not soluble in common organic solvents, including ethanol, methanol, acetonitrile, tetrahydrofuran, 1,4-dioxane and dimethoxyethane. The aqueous speciation of K_2_[Ir(OH)_6_] was therefore explored.

**Fig. 4:**
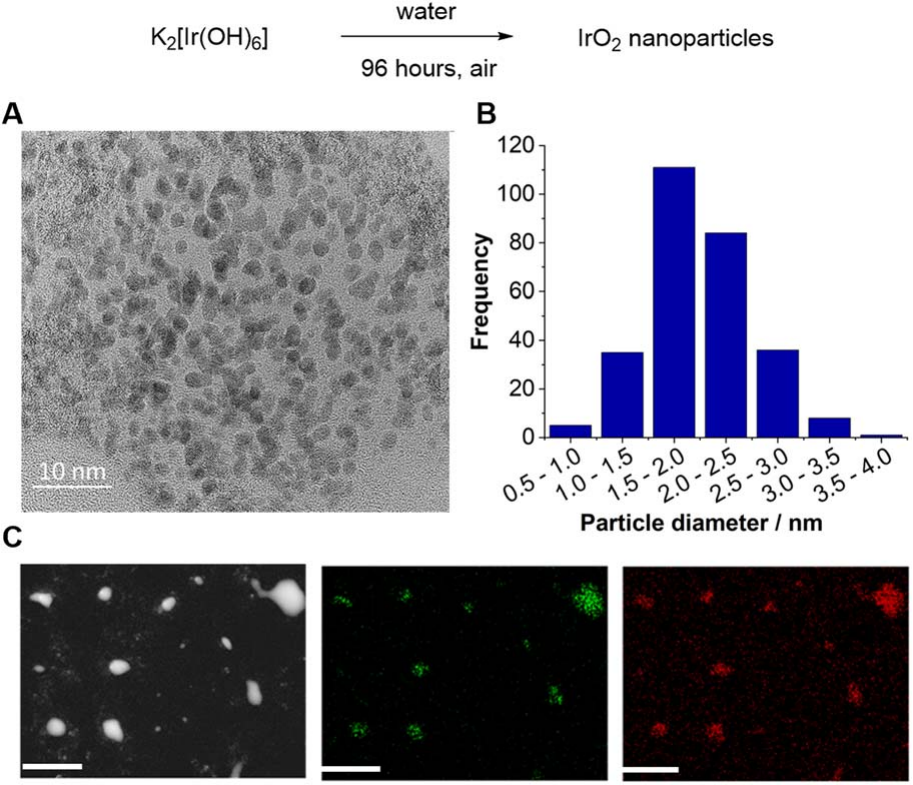
Analysis of iridium oxide nanoparticles formed from an aqueous solution of K_2_[Ir(OH)_6_] after storage in air for 96 hours. (A) HRTEM of dispersed IrO_2_ nanoparticles on a carbon grid. (B) Histogram of the IrO_2_ particle sizes showing a narrow particle size distribution with a 2.0 nm mean size and a standard deviation of 0.5 nm. (C) SEM images of the IrO_2_ nanoparticles, with energy dispersive X-ray maps of the Ir M edge (green) and the O K edge (red) showing the distribution of Ir and O in the clusters of nanoparticles. Scale bar in the SEM maps is 2.5 µm.

Immediately post dissolution in water (∼10 minutes open to air), a single [Ir(OH)_6_]^2−^ species is observed by ESI-MS with *m*/*z* 333.94355 (calc. [Ir(OH)_6_ + K]^−^ 333.94346 u) (Fig. S11). In air over 96 hours, K_2_[Ir(OH)_6_] in non-buffered aqueous solution undergoes a change in colour from purple to deep blue. UV-vis spectroscopy of the resulting solution shows the appearance of a broad absorbance band at 580 nm associated with iridium oxide nanoparticles (Fig. S37).^55,56^ The formation of IrO_*x*_·*n*H_2_O nanoparticles from solutions containing [Ir(OH)_6_]^2−^ has previously been reported through an acidic condensation process.^26^ High resolution TEM (HRTEM) (Fig. 4A) confirms the formation of IrO_2_ nanoparticles with a narrow size distribution of mean diameters of 2.0 nm. These small nanoparticles combine to form larger agglomerates (Fig. S38/39). The cubic crystalline structure of IrO_2_ was demonstrated by selected area electron diffraction (SAED) patterns obtained from these nanoparticle aggregates (Fig. S39). Scanning Electron Microscopy-Energy Dispersive X-ray (SEM-EDX) analysis performed on nanoparticle clusters clearly shows the presence of both Ir and O (Fig. 4C).

**Fig. 5:**
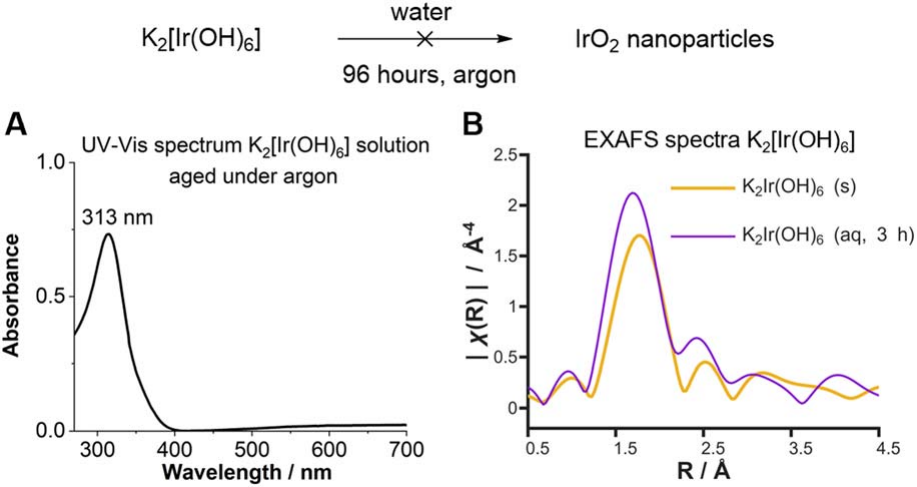
The behaviour of K_2_[Ir(OH)_6_] in aqueous solution under argon. (A) UV-vis spectrum post 96 hours. (B) Fourier-transformed Ir L_3_-edge EXAFS (R-space) spectra showing the magnitude of the *k*^2^-weighted Fourier transform of *χ*(*k*) (without phase correction) for K_2_[Ir(OH)_6_] in aqueous solution.

By contrast, under an argon atmosphere, iridium oxide nanoparticle formation is eliminated at experimentally relevant concentrations (10 mg mL^−1^) over a timescale of 96 hours. Solutions remain purple and the distinctive 580 nm UV-vis absorbance band is not observed (Fig. 5A). While X-ray absorption spectroscopy (XAS) data obtained three hours after dissolution in water show an oxidation state shift towards d^6^ Ir(iii) (Fig. S43), in contrast, an Evans measurement is consistent with a single unpaired electron (*µ*_eff_ = 1.90 *µ*_B_, Fig. S44 and S45). While the precise speciation is not currently resolved, one possibility that reconciles these data is the formation of an Ir(iii) oxyl complex [Ir(OH)_5_(O˙)]^3−^*via* a PCET process.^57^ This new species is likely mononuclear and pseudo-octahedral, as there is no evidence for a spatially nearby Ir neighbour from EXAFS (Fig. 5B), which only shows evidence for Ir–O proximity (∼1.8 Å).

Given these observations under both argon and air, subsequent synthetic procedures were conducted with freshly prepared solutions of K_2_[Ir(OH)_6_].

### The development of synthetic routes to simple Ir(iii) coordination complexes from K_2_[Ir(OH)_6_]

**Scheme 2:**
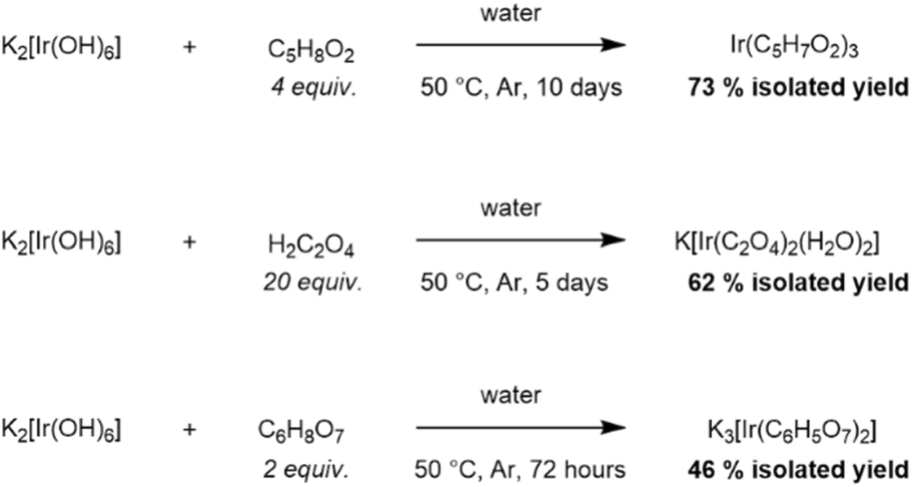
The synthesis of Ir(acac)_3_, *cis*- and *trans*-K[Ir(C_2_O_4_)_2_(H_2_O)_2_], and K_3_[Ir(C_6_H_5_O_7_)_2_] from K_2_[Ir(OH)_6_].

The applicability of K_2_[Ir(OH)_6_] as a platform intermediate for the synthesis of Ir(acac)_3_, K[Ir(C_2_O_4_)_2_(H_2_O)_2_] (oxalate) and K_3_[Ir(C_6_H_5_O_7_)_2_] (citrate) (Scheme 2) was next demonstrated. These are industrially relevant coordination complexes that have applications in homogeneous catalysis, the deposition of iridium oxide films,^3,58^ and Organic Light Emitting Diodes.^2^

### A high-yielding solution route to Ir(acac)_3_ from K_2_[Ir(OH)_6_]

The synthesis of Ir(acac)_3_*via* conventional solution state methods from chloroiridate precursors such as IrCl_3_·3H_2_O or K_2_[IrCl_6_] typically results in yields of below 25%.^7,59,60^ A higher yielding solution-state synthesis for Ir(acac)_3_ would offer a significant improvement. Whilst there is literature precedent for the reactions of metal hydroxides including Fe, Co, Ni, Ru and Re with stoichiometric quantities of acetylacetone to form M(acac)_3_ complexes,^61^ an attempt to synthesize Ir(acac)_3_ from K_2_[IrCl_6_] *via* an Ir(iii) hydroxide species achieved only a low overall yield of 10%.^60^ Here, a mild and efficient route to Ir(acac)_3_ with an isolated yield of 73% directly from K_2_[Ir(OH)_6_] is described.^62^ This represents a two-step yield from Na_2_[IrCl_6_] to Ir(acac)_3_ of 44%, which is a significant improvement on the 25% previously reported.^7,59,60^

An aqueous solution of K_2_[Ir(OH)_6_] (52 mg, 0.14 mmol) was heated with acetylacetone (4 equiv.) to 50 °C under argon for 10 days. The resulting water-insoluble yellow precipitate was separated from the crude reaction mixture by cannula filtration and dried under vacuum to yield Ir(acac)_3_ as a yellow solid (50 mg, 73%). The reduction of the paramagnetic Ir(iv) precursor to diamagnetic Ir(iii) requires a sacrificial equivalent of acetylacetone to be oxidised. The oxidation of acetylacetonate by hydroxide species has previously been demonstrated, with oxidation products including acetic acid.^63^ Here, potassium acetate is formed as a byproduct, with a singlet at *δ* 1.9 observed in the crude ^1^H NMR spectrum (Fig. S50).^64^

**Fig. 6:**
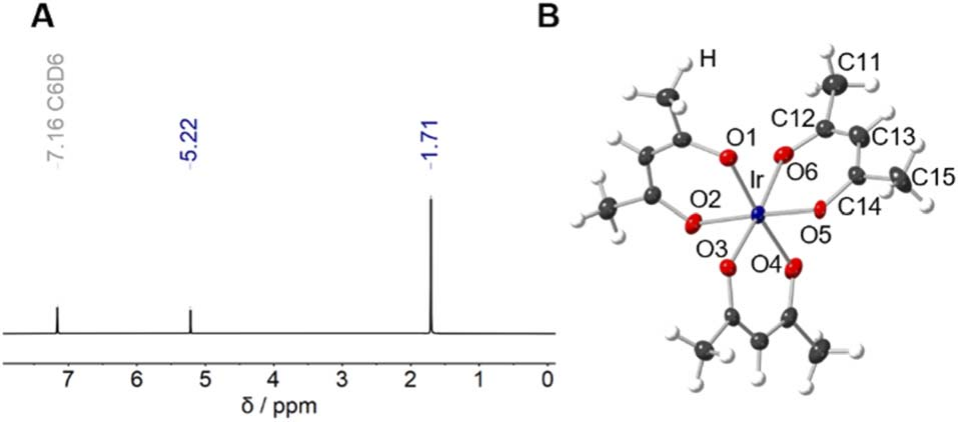
A) ^1^H NMR spectrum (600 MHz, 298 K, C_6_D_6_) of isolated Ir(acac)_3_ showing resonances at *δ* 5.22 and 1.71 ppm. (B) Molecular structure of Ir(acac)_3_ as determined by SC-XRD. Displacement ellipsoids are shown at the 50% probability level. Selected bond lengths (Å) and angles (°): Ir1–O6 2.027(19), O6–C12 1.278(3), C12–C11 1.502(4), C12–C13 1.397(4), Ir1–O6–C12 122.05(16), O6–C12–C13 126.2 (2), and O6–C12–C11 114.1(2). Hydrogen atoms were geometrically placed and allowed to ride on their parent atoms.

In the ^1^H NMR spectrum (benzene-*d*_6_) of isolated Ir(acac)_3_, two signals are observed at *δ* 5.22 and 1.71 with a 1 : 6 integration, respectively, matching previously reported data (Fig. 6A).^65^ Yellow needle-like crystals suitable for single-crystal X-ray diffraction were grown from the slow diffusion of a benzene-*d*_6_: pentane layered solution under argon. SC-XRD confirms the structure and offers an improvement of that previously reported for this complex (*R*_1_ = 1.53% compared to *R*_1_ = 4.4%), Fig. 6B.^66^ The iridium(iii) center sits in an octahedral coordination environment with an average Ir–O distance of 2.017 Å and an average O–Ir–O chelate angle of 95.13° (Fig. 6B).

### The synthesis of iridium oxalate species from K_2_[Ir(OH)_6_]: the isolation and characterisation of *cis*- and *trans*-K[Ir(C_2_O_4_)_2_(H_2_O)_2_]

The synthesis of iridium oxalate complexes such as Na[Ir(C_2_O_4_)_2_(H_2_O)_2_] and K_3_[Ir(C_2_O_4_)_3_] from chloroiridate precursors is known, but typified by moderate yields, long reaction times and high temperatures.^67–73^ The challenge in achieving high yields has been attributed to the difficulty in isolating the desired complexes from crude reaction solutions.^72^ Complexes of *trans*-X[Ir(C_2_O_4_)_2_(H_2_O)_2_] (X = Na^+^, [H_5_O_2_]^+^) are known,^74^ but the synthesis and characterisation of *cis*-[Ir(C_2_O_4_)_2_(H_2_O)_2_]^−^ have not been previously reported with any counterion. The synthesis of K[Ir(C_2_O_4_)_2_(H_2_O)_2_] from K_2_[Ir(OH)_6_] is now demonstrated under milder conditions and in shorter reaction times compared to previously reported procedures starting from chloroiridate precursors.^67–73^

K_2_[Ir(OH)_6_] (30 mg, 0.081 mmol) and oxalic acid (21 equiv.) were dissolved in water under argon and heated to 50 °C for five days. Work-up by addition of tetra-*n*-butylammonium hydrogen sulfate, extraction with nitromethane, and addition of potassium acetate in ethanol resulted in the isolation of a fine yellow precipitate of K[Ir(C_2_O_4_)_2_(H_2_O)_2_] as a water soluble, pale-yellow solid (21 mg, 62%).

This crude product is isolated as a mixture of the *cis*- and *trans*-isomers of K[Ir(C_2_O_4_)_2_(H_2_O)_2_], as determined by IR spectroscopy (Fig. S14). Selective recrystallisation procedures using different antisolvents enable the isolation of crystalline *cis*- and *trans*-K[Ir(C_2_O_4_)_2_(H_2_O)_2_].

**Fig. 7:**
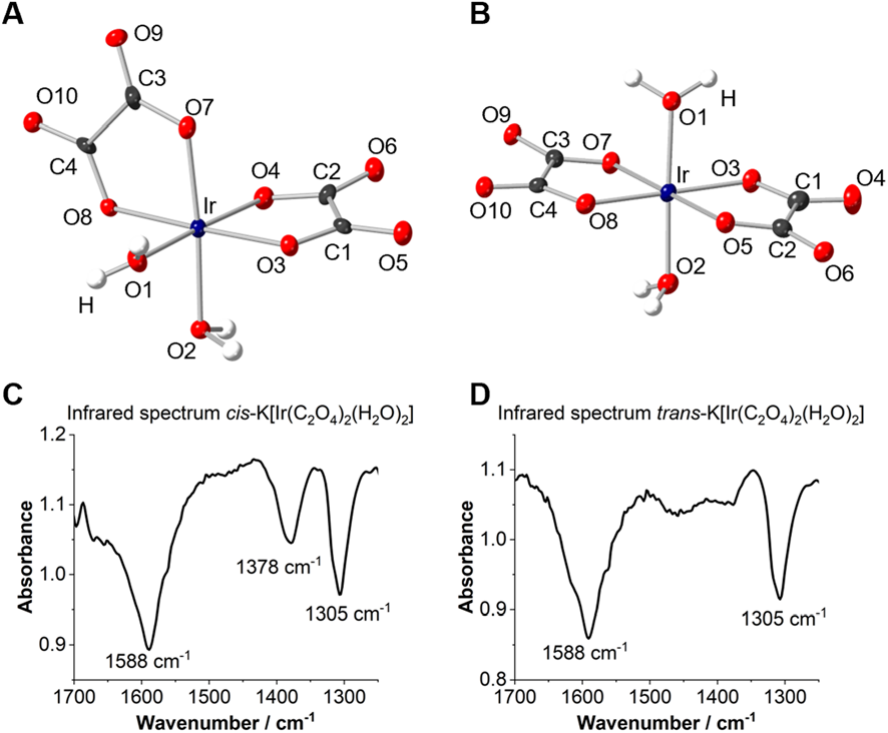
(A) Molecular structure of *cis*-K[Ir(C_2_O_4_)_2_(H_2_O)_2_] as determined by single-crystal XRD. (B) Molecular structure of *trans*-K[Ir(C_2_O_4_)_2_(H_2_O)_2_] as determined by single-crystal XRD. Displacement ellipsoids are shown at the 50% probability level. Counterion and lattice water molecules are excluded for clarity. Hydrogen atoms were geometrically placed and allowed to ride on their parent atoms. (C) *v*(CO) region of the IR spectrum of *cis*-K[Ir(C_2_O_4_)_2_(H_2_O)_2_]. (D) *v*(CO) region of the IR spectrum of higher symmetry *trans*-K[Ir(C_2_O_4_)_2_(H_2_O)_2_].

Single crystals of *cis*-K[Ir(C_2_O_4_)_2_(H_2_O)_2_]·2H_2_O were grown as yellow needles from the slow diffusion of a water: tetrahydrofuran layered solution under argon (Fig. 7A). *cis*-K[Ir(C_2_O_4_)_2_(H_2_O)_2_]·2H_2_O crystallizes in the *P*1̄ space group (*R*_1_ = 2.95%) with two solvent water molecules per iridium complex in the structure. The geometry around the iridium centre is a distorted octahedron: the bidentate oxalate ligands are bound with bite angles (°) of 81.41(17) and 82.17(18). One oxalate is bound with Ir–O bond lengths (Å) of Ir–O4 2.043(5) and Ir–O3 2.047(4), whilst the other exhibits a greater degree of asymmetry with Ir–O8 2.047(4) and Ir–O7 2.027(5). Intermolecular hydrogen bonding is observed from coordinated water to coordinated oxalate ligands on adjacent octahedra, with an O_D_(H)⋯O_A_ distance (Å) of 2.643(6) (Fig. S63).

Single crystals of *trans*-K[Ir(C_2_O_4_)_2_(H_2_O)_2_]·½H_2_O were grown as yellow needles from the slow diffusion of a water: isopropylalcohol layered solution under argon (Fig. 6B). The crystal structure of *trans*-K[Ir(C_2_O_4_)_2_(H_2_O)_2_]·½H_2_O was solved in the *P*1̄ space group (*R*_1_ = 2.27%), with an asymmetric unit comprising two octahedral iridium centers of [Ir(C_2_O_4_)_2_(H_2_O)]^−^, two potassium ions and one water molecule. The geometry around the iridium center is a distorted octahedron and the oxalate ligands are bound with bite angles (°) of 80.98(13) and 81.53(12). The average Ir–O (oxalate) bond length is 2.039(3) Å, and each coordinated oxalate has highly symmetric Ir–O bond lengths (*i.e.* to within 0.005 Å). A slightly longer average Ir–O distance of 2.060(3) Å is observed for coordinated water. Weak intermolecular hydrogen bonding between coordinated water and the terminal oxygen of coordinated oxalate ligands on adjacent octahedra is observed with an O_D_(H)⋯O_A_ distance (Å) of 2.648(5) (Fig. S61).

Confirmation that the single crystals are representative of the bulk material is provided by infrared spectroscopy (Fig. 7C/D). In the *v*(CO) region, *trans*-K[Ir(C_2_O_4_)_2_(H_2_O)_2_] exhibits two strong bands (1588s *v*_a_(C=O) and 1305s *v*_s_(C–O) cm^−1^) whilst *cis*-K[Ir(C_2_O_4_)_2_(H_2_O)_2_] has three (1588s *v*_a_(C=O), 1378 *v*_s_(C–O) and 1305s *v*_s_(C–O) cm^−1^). Furthermore, the diffraction patterns observed by PXRD of the bulk crystalline material generated from each set of recrystallisation conditions show no overlap (Fig. S17 and S19).

Both isomers of K[Ir(C_2_O_4_)_2_(H_2_O)_2_] are insoluble in common organic solvents, limiting solution-state characterisation to aqueous conditions. ^13^C{^1^H} NMR spectra recorded immediately post dissolution in D_2_O show a single resonance for *trans*-K[Ir(C_2_O_4_)_2_(H_2_O)_2_] at *δ* 171.2, and two resonances for *cis*-K[Ir(C_2_O_4_)_2_(H_2_O)_2_] at *δ* 171.5 and *δ* 171.1 (Fig. S18 and S20). By CV, UV-vis spectroscopy and ESI-MS, aqueous solutions of *cis*- and *trans*-K[Ir(C_2_O_4_)_2_(H_2_O)_2_] are indistinguishable.^75^

### The synthesis of a previously unknown complex from K_2_[Ir(OH)_6_]: a novel iridium citrate species, K_3_[Ir(C_6_H_5_O_7_)_2_]

Several base metal citrate complexes are known, including those of iron,^76,77^ manganese,^78^ and cobalt.^79,80^ In PGM chemistry, citrate is commonly used as a stabilizer and reducing agent for the controlled formation of nanoparticles.^81–84^ Though the anti-cancer properties of a rhodium citrate dimer have been studied,^85,86^ complexation to form a discrete, monomeric PGM-citrate complex has not been reported to date. As demonstrated by their relative redox potentials, [Ir(OH)_6_]^2−^ (*E*_1/2_ = −0.44 V *vs.* Ag/AgCl) is more difficult to reduce than [IrCl_6_]^2−^ (*E*_1/2_ = +0.74 V),^87^ enabling the exploration of coordination chemistry with more reducing reactants such as citric acid. K_3_[Ir(C_6_H_5_O_7_)_2_] is now reported as the first example of an isolated iridium citrate complex.

K_2_[Ir(OH)_6_] (30 mg, 0.081 mmol) and citric acid (2 equiv.) were combined in water and stirred under an argon atmosphere for 72 hours at 50 °C to form a cloudy yellow solution. The solvent was removed *in vacuo* to form a yellow oil. Trituration with methanol gave K_3_[Ir(C_6_H_5_O_7_)_2_] as a yellow-brown, analytically pure, amorphous powder (26 mg, 46%).

**Fig. 8:**
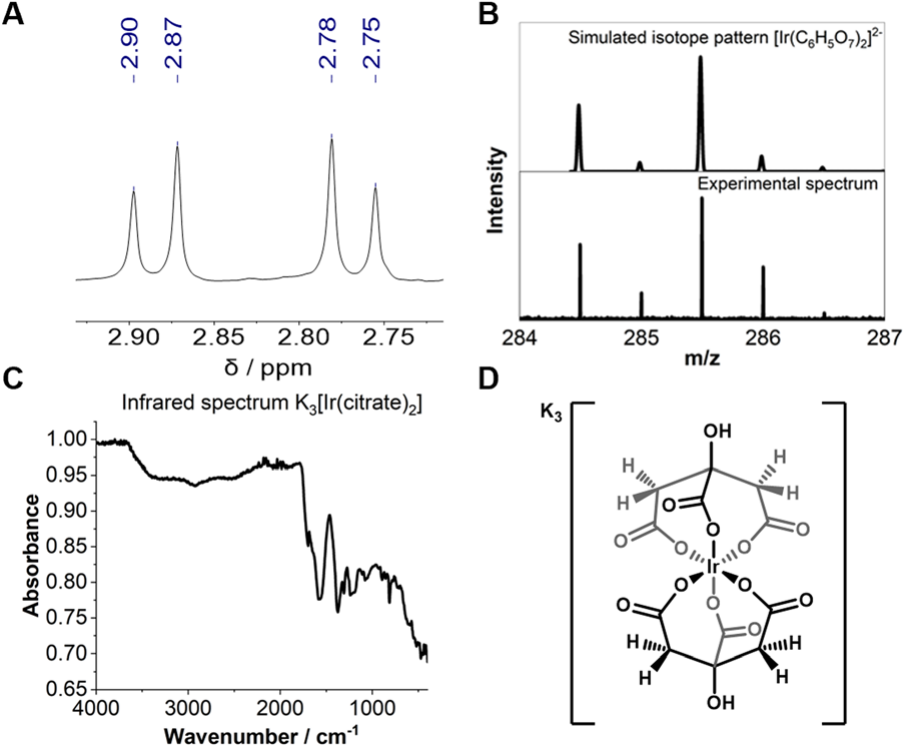
The characterisation of K_3_[Ir(C_6_H_5_O_7_)_2_]. (A) ^1^H NMR spectrum (600 MHz, D_2_O, 298 K). (B) Mass spectrum of K_3_[Ir(C_6_H_5_O_7_)_2_] in water showing match to the simulated isotope pattern for [Ir(C_6_H_5_O_7_)_2_]^2−^. (C) Infrared spectrum of K_3_[Ir(C_6_H_5_O_7_)_2_]. (D) Proposed structure of K_3_[Ir(C_6_H_5_O_7_)_2_].

K_3_[Ir(C_6_H_5_O_7_)_2_] has partial solubility in water. It exhibits a roofed doublet characteristic of citrate in the ^1^H NMR spectrum (doublets at *δ* 2.88 and 2.77), shifted upfield compared to free citric acid (doublets at *δ* 2.99 and 2.81). In the ^1^H NMR spectrum, sharp peaks are observed, suggesting that reduction to a diamagnetic iridium(iii) species has occurred (Fig. 8A). Negative mode ESI-MS confirms that the iridium centre is chelated by two citrate ligands (Fig. 8B). A doubly charged anion [Ir(C_6_H_5_O_7_)_2_]^2−^ is observed at *m*/*z* 285.49759 (calc. 285.48557*u*), matching the simulated isotope pattern. The double negative charge is attributed to ionization effects and the instability of triply charged anions in the gas phase. The infrared spectrum shows a broad absorbance band between 2500 and 3500 cm^−1^ associated with the *v*(O–H) band and asymmetric and symmetric *v*(COO^−^) bands observed at 1587 and 1368 cm^−1^ respectively.

## Conclusions

We show that K_2_[Ir(OH)_6_] can successfully fulfil the function of a soluble, halide-free, platform intermediate that sits between complex post-refinery chloroiridate mixtures and technologically useful late-stage Ir(iii) precursors such as Ir(acac)_3_. Rigorous speciation studies resulted in the identification of analytical markers for the synthesis of this simple homoleptic complex, allowing for the development of a repeatable synthetic protocol. Whilst the yields of this complex using chloroiridate refinery streams are currently moderate, halide-free K_2_[Ir(OH)_6_] offers several advantages over the conventional intermediate [NH_4_]_2_[IrCl_6_]: the presence of trace amounts of chloride can result in downstream catalyst poisoning, and subsequent synthetic routes from K_2_[Ir(OH)_6_] are reported with higher yields, improved selectivity, and access to new complexes. This methodology has the potential to offer a scalable and analytically testable route to the synthesis of simple halide-free Ir(iii) precursor complexes from complex aqueous refinery streams.

## Author contributions

R. M. W. performed all the synthetic and methodological experimental work and wrote all the major drafts of the manuscript. J. H. H. and K. M. A.: crystallographic analysis. S. K. B.: XAS and XAFS analysis. A. J. E.: single-crystal neutron diffraction. M. A. H.: squid analysis. B. F. and V. K. L.: HRTEM analysis. N. M. M. and D. T.: industrial supervision of the project. J. A. M., A. S. W. and C. E. W.: writing – review and editing, supervision and project conceptualisation.

## Conflicts of interest

There are no conflicts to declare.

## Supplementary Material

SC-OLF-D6SC05573G-s001

SC-OLF-D6SC05573G-s002

## Data Availability

CCDC 2554247–2554252 contain the supplementary crystallographic data for this paper.^88*a*–*f*^ Supplementary information (SI): the SI contains full experimental and characterisation data for the new complexes, including single crystal X-ray crystallographic collection and refinement details. See DOI: https://doi.org/10.1039/d6sc05573g.
