# Supplementary material for: K2[Ir(OH)6]: an isolable platform intermediate for the refining of complex chloroiridate mixtures to synthetically useful Ir(iii) coordination complexes
Source: Chem Sci. 2026 Jul 27;17(37):17990–8000. doi: 10.1039/d6sc05573g (PMC13450093; doi:10.1039/d6sc05573g)
Supplement: Supplementary file 1 [file SC-017-D6SC05573G-s001.pdf]

Supplementary Information (SI) for Chemical Science.
This journal is © The Royal Society of Chemistry 2026

Electronic Supporting Information for:

$K_2[Ir(OH)_6]$: An isolable platform intermediate in the refining of complex chloroiridate mixtures into synthetically useful Ir(III) coordination complexes

Rebecca M. Willans,[a] Jack H. Heaton,[a] Kristof M. Altus,[a] Simon K. Beaumont,[b] Alison J. Edwards,[c] Michael A. Hayward,[d] Ben Freeman,[e] Vlado K. Lazarov,[e] Nicoleta M. Muresan,[f] David Thompsett,[f] Jackie A. Mosely,[a,*] Andrew S. Weller,[a,*] Charlotte E. Willans.[a,*]

[a] Department of Chemistry, University of York, York, YO10 5DD, UK.

[b] Department of Chemistry, Durham University, South Road, Durham, DH1 3LE, UK.

[c] Australian Centre for Neutron Scattering, Australian Nuclear Science and Technology Organization, Lucas Heights, New South Wales 2234, Australia.

[d] Department of Chemistry, Inorganic Chemistry Laboratory, University of Oxford, South Parks Road, Oxford, OX1 3QR, UK.

[e] York JEOL Nanocentre, Helix House, York Science Park, University of York, Heslington, York, YO10 5BR, UK.

[f] Johnson Matthey Technology Centre, Blounts Court Road, Sonning Common, Reading, RG4 9NH, UK.

1.0. Contents

1.0. Contents 2

2.0. General considerations 4

3.0. Nano-electrospray ionisation mass spectrometry (nESI-MS) 6

3.1. nESI-MS experimental method 6

3.2. Analysis of post-refinery chloroiridate solution by nESI-MS at different timepoints post dilution 8

3.3. Analysis of post-refinery chloroiridate solution by nESI-MS at different pHs 11

4.0. Synthesis and characterisation 12

4.1. $K_2[Ir(OH)_6]$ 12

4.2. $Ir(acac)_3$ (acac = $C_5H_7O_2$) 13

4.3. $K[Ir(C_2O_4)_2(H_2O)_2]$ (oxalate = C_2O_4) 14

4.4. $K_3[Ir(citrate)_2]$ (citrate = $C_6H_5O_7$) 18

4.5. $[NBu_4]_2[Ir_2Cl_{10}]$ 21

4.6. $K_2[Ir(OH)_n(OD)_{6-n}]$ (n = 0 – 6) 25

5.0. Optimisation and reaction monitoring of the synthesis of $K_2[Ir(OH)_6]$ 29

5.1. Attempted synthesis of $K_2[Ir(OH)_6]$ following literature reported conditions 29

5.2. Adaptations of protocol 30

5.3. Supplementary reaction monitoring data during the synthesis of $K_2[Ir(OH)_6]$ from $Na_2[IrCl_6]$ 31

5.4. The synthesis of $K_2[Ir(OH)_6]$ from a crude post-refinery chloroiridate solution, $H_2[IrCl_6]$ in HCl 32

6.0. The temporal behaviour of $K_2[Ir(OH)_6]$ 33

6.1. Air-aged crystalline $K_2[Ir(OH)_6]$ 33

6.2. Air-aged aqueous solutions of $K_2[Ir(OH)_6]$ 35

6.3. Argon-aged aqueous solutions of $K_2[Ir(OH)_6]$ 37

7.0. Optimisation of the synthesis of $Ir(acac)_3$ from $K_2[Ir(OH)_6]$ 41

7.1. Tabulated conditions 41

7.2. NMR spectra of crude reaction solutions 42

7.3. The synthesis of $Ir(acac)_3$ from a 96-hour argon aged aqueous solution of $K_2[Ir(OH)_6]$ 45

8.0. The temporal solution-state behaviour of $K[Ir(C_2O_4)_2(H_2O)_2]$ 47

9.0. Single-crystal diffraction data 48
9.1. Experimental details 48
9.2. $K_2[Ir(OH)_6]$ 49
9.3. $Ir(acac)_3$ 52
9.4. *Cis-/trans*-$K[Ir(C_2O_4)_2(H_2O)_2]$ 54
10.0. References 57

2.0. General considerations

Where stated, manipulations were performed under an inert (argon or nitrogen, BOC, N4.8 purity) atmosphere using standard Schlenk line and glovebox (<0.1 ppm H_2O/O_2) techniques. Glassware was oven dried at 140 ^{o}C overnight. Solvents were degassed and stored over activated 3 Å molecular sieves under inert gas in resealable glass ampoules fitted with PTFE high vacuum stopcocks (J. Youngs or Rotaflo HP). Tetrahydrofuran (THF) and n-pentane were degassed by three successive freeze-pump-thaw cycles. Pentane was dried using a commercially available solvent drying system (MBraun) by passage through stainless steel columns packed with activated alumina. THF was dried over Na/ fluorophenone in a solvent still. Methanol and isopropylalcohol were sparged with argon and stored over activated 3 Å molecular sieves under inert gas. Deionised water was sparged with argon and stored under argon.

NMR spectra were obtained using a Bruker AVIIIHD 600 Widebore instrument 600 MHz [^{1}H], 151 MHz [^{13}C] or a Bruker Avance III NMR spectrometer 500 MHz [^{1}H], 126 MHz [^{13}C]. Chemical shifts (δ) are reported in parts per million (ppm) and coupling constants (J) are reported in Hz. ^{1}H and ^{13}C{^{1}H} NMR spectra are referenced to residual solvent signals unless otherwise stated. Spectral data was collected at 298 K. NMR spectra were processed using MestReNova software (version 14.3.2).

Mass spectrometry data was obtained using a Bruker SolariX XR FTMS 9.4 T hybrid Qh-FT-ICR instrument by electrospray ionisation (ESI), nano-electrospray ionisation (nESI) or laser desorption ionisation (LDI) as specified. Data were processed using Compass DataAnalysis and OriginPro 2023b software. Simulated isotope patterns were generated within the Compass DataAnalysis software.

IR spectroscopy was performed using a Bruker ALPHA II Compact FT-IR Spectrometer with a platinum-diamond ATR sampling module. Unless otherwise specified, measurements were conducted in air. Raman spectra were measured using a Renishaw inVia Raman spectrometer using a 532 nm laser source.

UV-Vis spectroscopy data were obtained using a JASCO V-560 UV-Vis spectrophotometer fitted with a Julabo F12 thermostatically controlled water bath and circulator. Measurements were recorded in air with 10 mm or 1 mm path length quartz cuvettes, unless otherwise specified. For measurements obtained under argon, Schlenk-adapted quartz cuvettes (10 mm or 1 mm path length) were subjected to three vacuum and refill cycles prior to use. Data was collected at 298 K.

Cyclic voltammograms were collected using either an Metrohm Autolab potentiostat or a Metrohm DropSens µStat. Two different set-ups were used as specified. Screen printed electrodes (Eluceda) with custom carbon ink working and counter electrodes and silver ink reference electrode printed onto a PVC substrate were connected to a potentiostat using PalmSens adaptor PS-CONN-4MM. Alternatively, cyclic voltammograms were measured using

a Schlenk-adapted Asynt ElectroReact with glassy carbon working electrode, platinum foil counter-electrode and a polyetheretherketone 1.6 mm diameter LF-1.6 Leak-Free Ag/AgCl Reference Electrode (Innovative Instruments, Inc.). Data were analysed using the Nova 2.1.6 software and OriginPro 2023b.

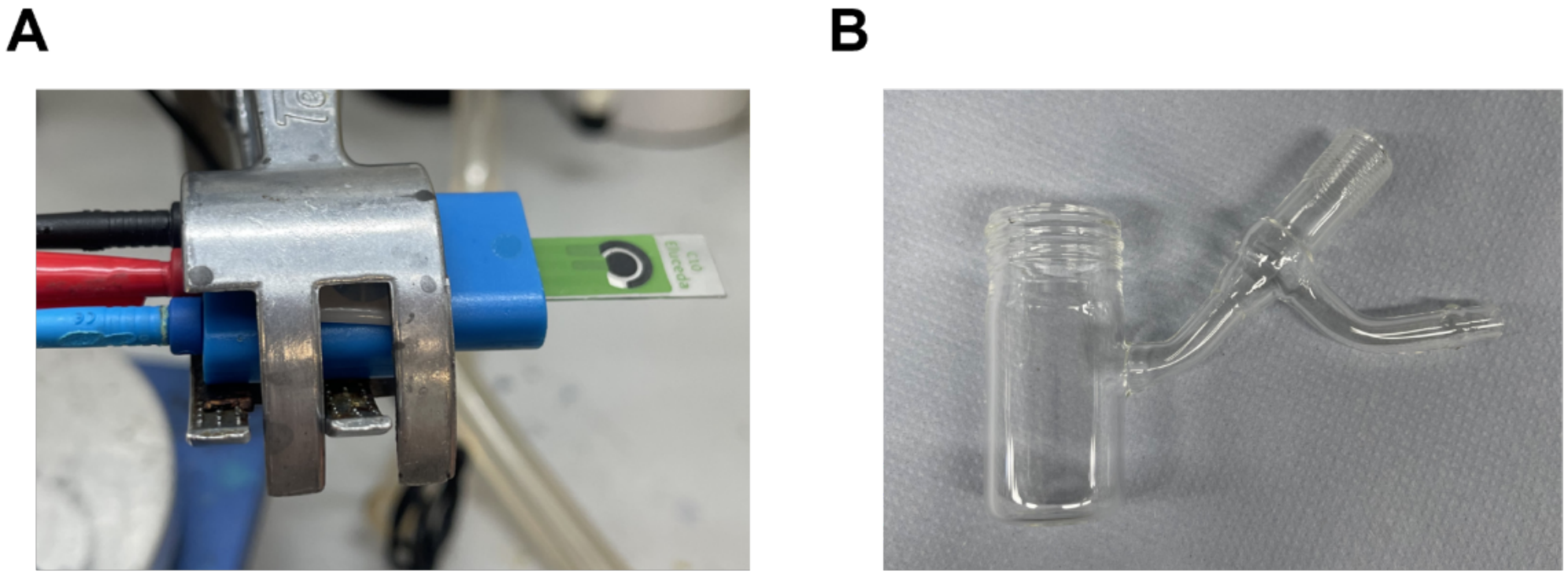

Figure S1. Equipment used for cyclic voltammetry set-ups: A screen printed electrode connected via PalmSens adaptor; B in-house Schlenk-adapted Asynt Electroreact.

Powder X-ray crystallography was performed on a Panalytical Aeris X-ray diffractometer equipped with a 600 W copper source and a PIXcel1D-Medipix3 detector. The instrument was operated in reflectance mode, with a scan range of $2\theta = 5–70^{o}$ and a scan rate of 0.026^{o} s^{-1} .

Magnetisation data were collected using a Quantum Design MPMS3 SQUID magnetometer, from a sample of $K_2[Ir(OH)_6]$ (25.8 mg) which had been sealed under vacuum in a spectrosil sample holder. Magnetisation data were collected as a function of temperature on warming from 5 K to 300K in an applied field of 10000 Oe, after the sample had been first cooled from 300K to 5K in an applied field of 0 Oe (zero-field cooled) and then an applied field of 1000 Oe (field cooled). Magnetisation data were fit to the formula: $M/H = C/(T- \theta) + K$ in the temperature range $70 < T/K < 300$, to yield values of C = 0.450(3) cm^3 K mol^{-1}, θ = -11.9(4) K and K = -4.11(6) x 10^{-5} cm^3 mol^{-1}. The inset to Figure 3, Panel H in the main text shows the linear fit to a plot of H/(M-K) against T (i.e. a plot of the inverse of the magnetic susceptibility against temperature, after the temperature independent diamagnetic contribution to the magnetisation (K) had been removed) confirming that the data could be fit by the Curie-Weiss law in the temperature range $70 < T/K < 300$.

3.0. Nano-electrospray ionisation mass spectrometry (nESI-MS)

Techniques to demonstrate the complexity of speciation in the post-refinery chloroiridate solution were sought. By conventional electrospray ionisation mass spectrometry (ESI-MS), high concentration acidic, metal containing solutions cannot be analysed without risking damage to the mass spectrometer. nESI-MS, through its lower flow-rate, enables samples of this type to be analysed.[3] Method development was conducted to enable analysis of the crude, post-refinery chloroiridate solution.

3.1. nESI-MS experimental method

A 9.4 T SolariX FT-ICR mass spectrometer was equipped with an offline nanospray source built in-house. Needles were pulled from borosilicate glass capillaries (World Precision Instruments, TW100-4) using a Sutter Instruments Co. flaming/ brown micropipette puller (model P-97) with the following programme parameters: P=500, heat=826, pull=10, vel=40, del=250. This protocol was optimised for most stable signal over the acquisition period. A solution of the analyte (1 mM) was injected into the glass needle using a microsyringe, and a platinum wire fed into the back of the needle to the point where the glass tapered. The other end of the wire was connected to the API source. The tip of the needle was positioned ~ 1 mm from the nano-electrospray source and an API voltage of 1000 V applied. Data was collected in serial acquisition mode over 15 minutes.

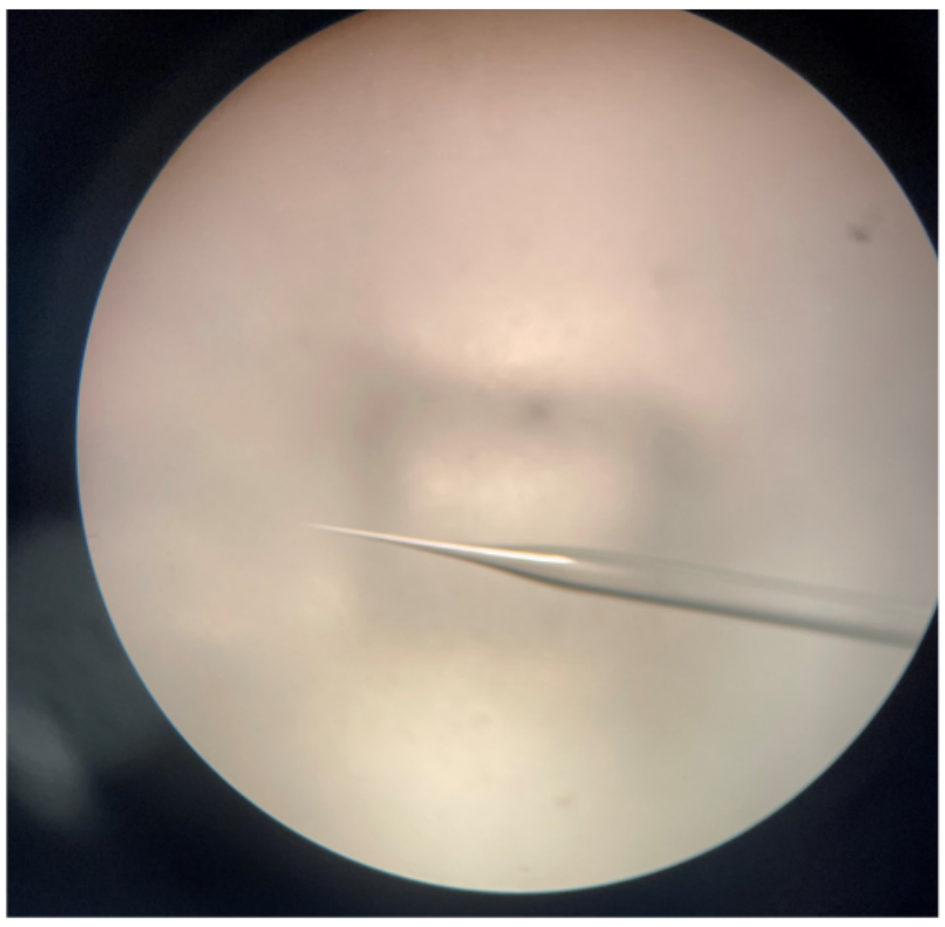

Figure S2. Needle pulled from glass capillary into which analyte solution injected. Tip shape optimised for the most stable signal over the 15-minute acquisition period.

3.1.1. Method development

Initially, speciation changes were observed upon application of API voltage to analyte solution. Reduction in API voltage to 1000 V resulted in no observable speciation changes.

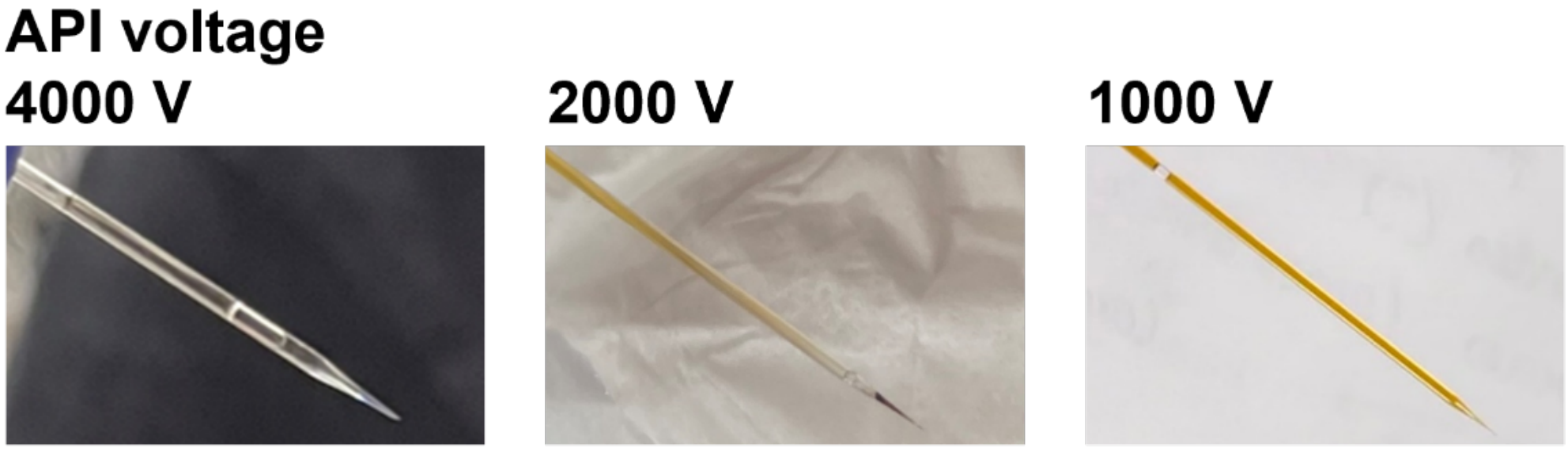

Figure S3. Selected example of the optimisation of nESI-MS: application of higher ionisation voltages resulted in speciation changes observed in the analyte solution (post-refinery chloroiridate solution, diluted to 1 mM) by colour change from brown to colourless and/or formation of a black precipitate. At 1000 V, no observable difference in analyte solution before and after application of voltage.

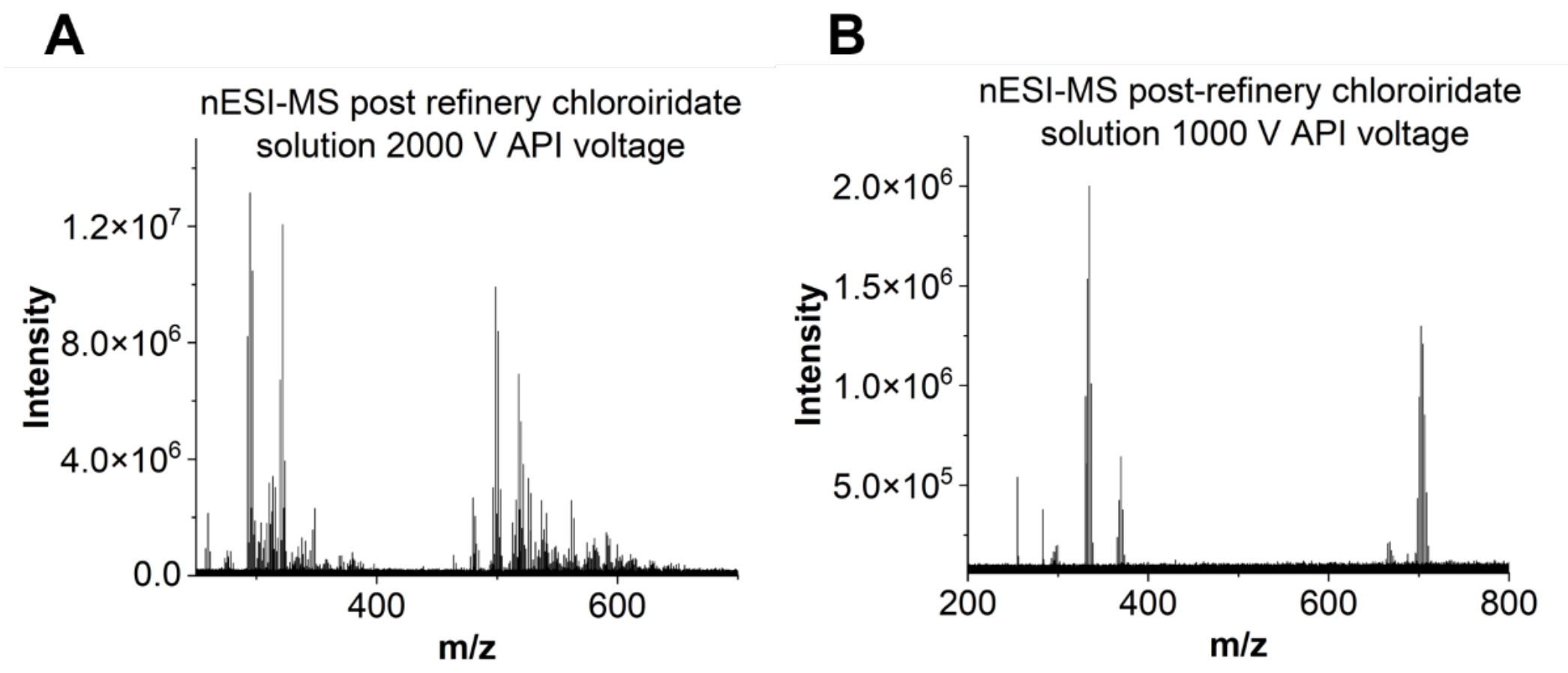

Figure S4. Mass spectra collected during the optimisation of the nano-electrospray ionisation mass spectrometry method through variation of API voltage to avoid speciation changes observed, described in Figure S3. **A** API voltage of 2000 V; **B** API voltage of 1000 V showing cleaner mass spectrum obtained.

3.2. Analysis of post-refinery chloroiridate solution by nESI-MS at different timepoints post dilution

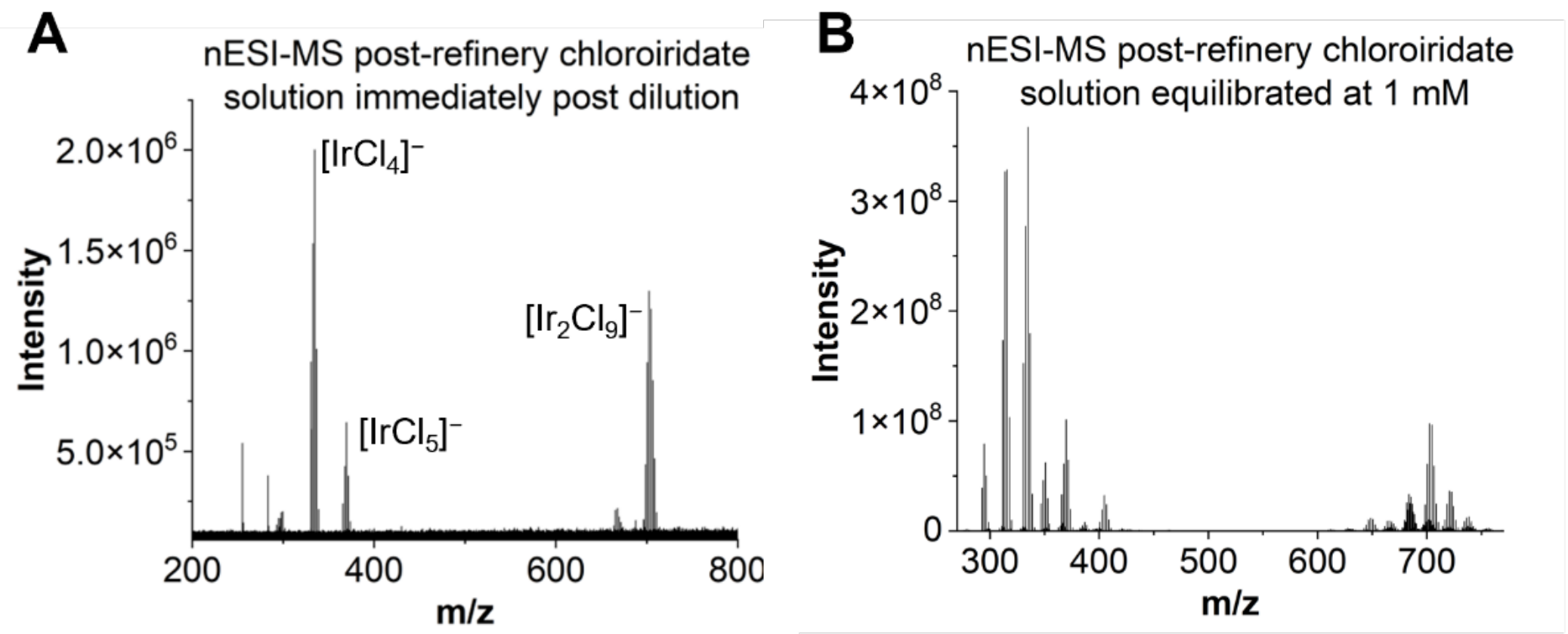

Figure S5. Mass spectra of crude post-refinery solution diluted to 1 mM and run by nESI-MS at an API voltage of 1000 V. Data collected in serial acquisition mode over 15 minutes. **A** Immediately post dilution (10 minutes) post-refinery chloroiridate solution from 21.85 % by weight to 1 mM [Ir]; **B** Post-refinery chloroiridate solution aged at 1 mM [Ir] for six months in air, sealed.

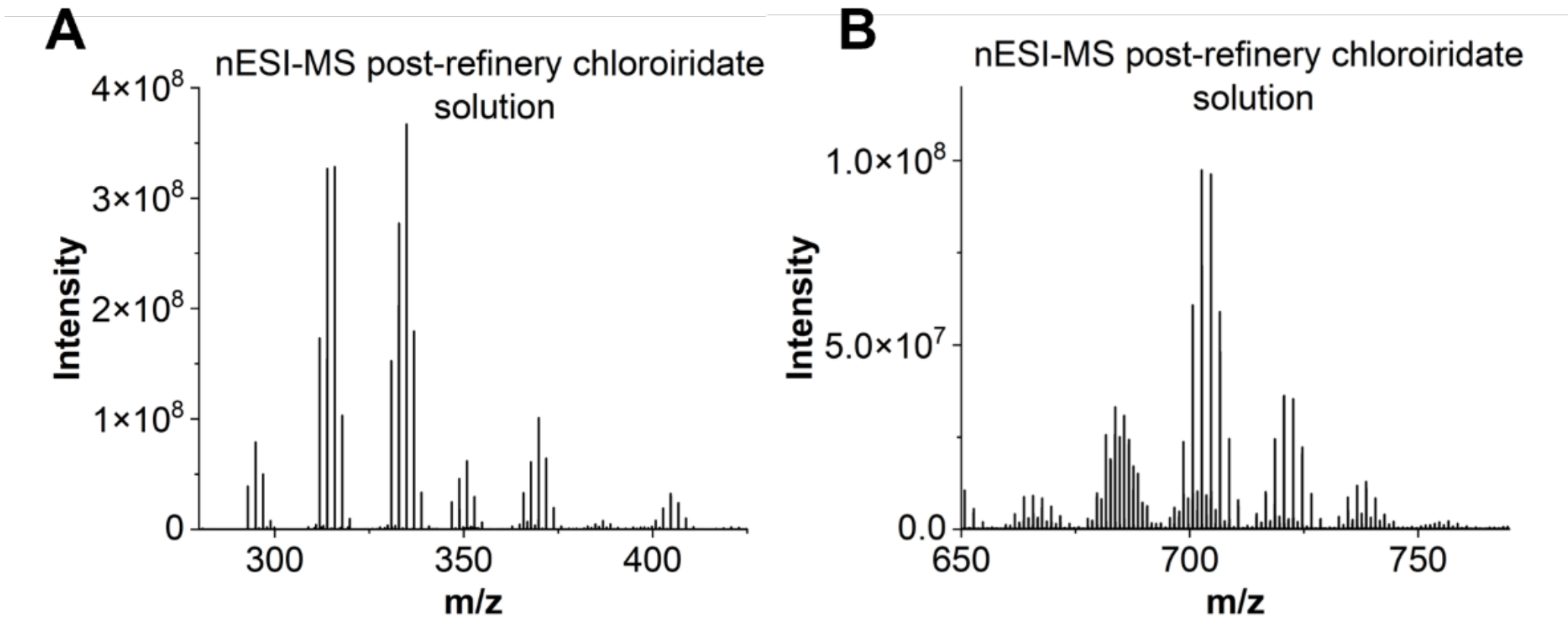

Figure S6. Mass spectra, collected by nESI-MS, of crude chloroiridate solution diluted to 1 mM and aged for six months in air. Zoomed in spectra to show isotope patterns.

Major monomeric species identified in six month aged 1 mM [Ir] sample: $[IrCl_2O_2]^-$, $IrCl_3(OH_2)]^-$, $[IrCl_4]^-$, $[IrCl_4O]^-$, $[IrCl_5]^-$, $[IrCl_5(OH_2)]^-$, $[IrCl_6]^-$.

Major dimeric species identified in six month aged 1 mM [Ir] sample (note – may be forming during mass spectrometry as a result of in-source dimerization): $[Ir_2Cl_{10}]^-$, $[Ir_2Cl_9]^-$, $[Ir_2Cl_9(OH_2)]^-$, $[Ir_2Cl_8O]^-$, $[Ir_2Cl_7(OH_2)]^-$, $[Ir_2Cl_7O]^-$.

3.2.1. Simulated and experimental isotope patterns of species observed in the six-month aged 1 mM [Ir] post-refinery chloroiridate solution

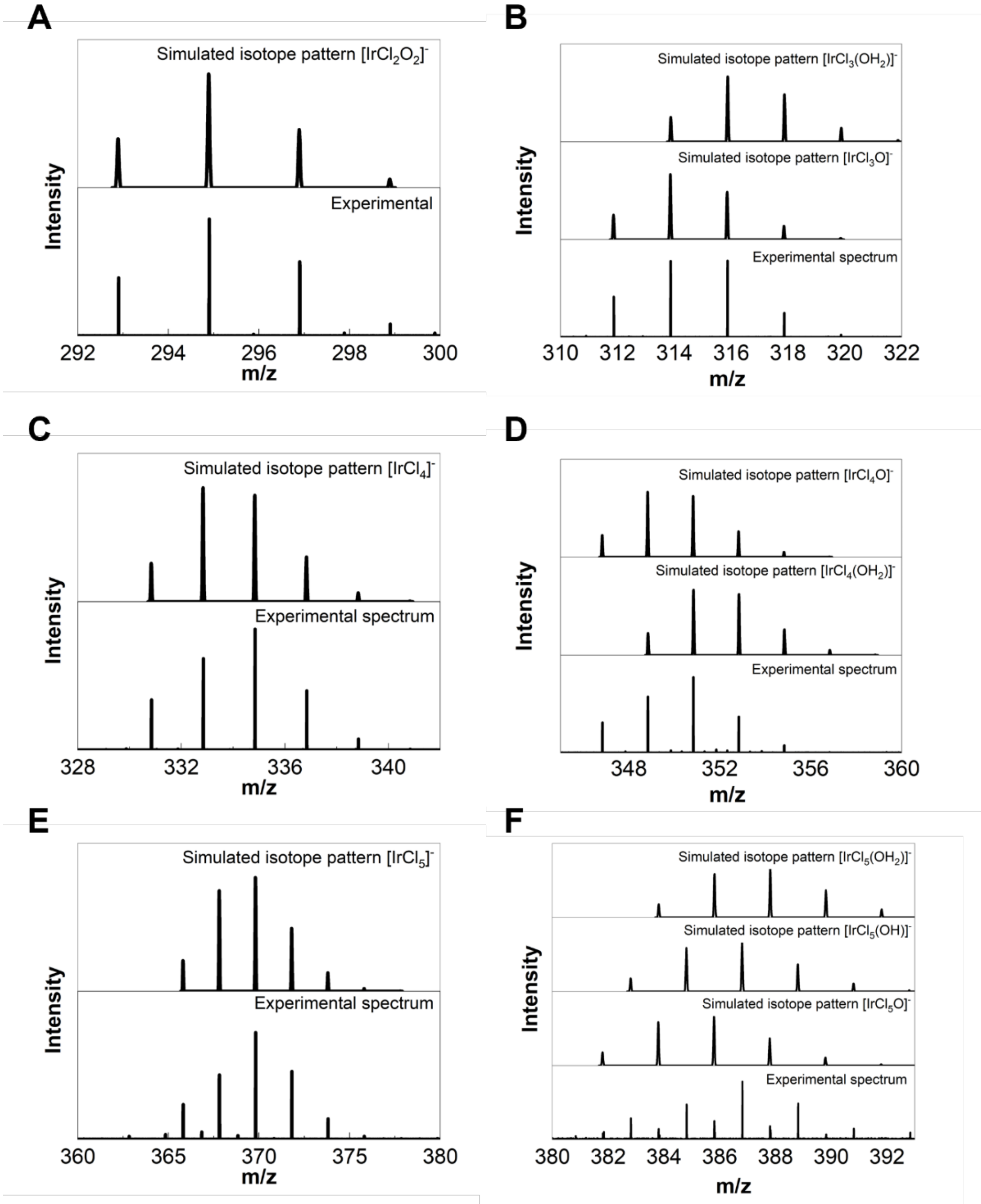

Figure S7. Comparison of experimentally observed isotope patterns with patterns simulated in the Bruker DataAnalysis software for monomeric species observed in mass spectrum of the post-refinery chloroiridate solution equilibrated for six months at 1 mM [Ir].

3.2.2. Tandem mass spectrometry (MS^2)

$[IrCl_4]^-$ and $[IrCl_5]^-$ are observed in the mass spectrum acquired immediately post dilution of the crude post-refinery chloroiridate solution. Tandem mass spectrometry experiments show that $[IrCl_5]^-$ is a fragment of $[IrCl_6]^{2-}$ forming during ion transfer after quadrupolar isolation of $[IrCl_6]^{2-}$. MS^2 of $[IrCl_5]^-$ shows fragmentation into $[IrCl_4]^-$. Therefore, both $[IrCl_5]^-$ and $[IrCl_4]^-$ can form from $[IrCl_6]^{2-}$ in the gas phase.

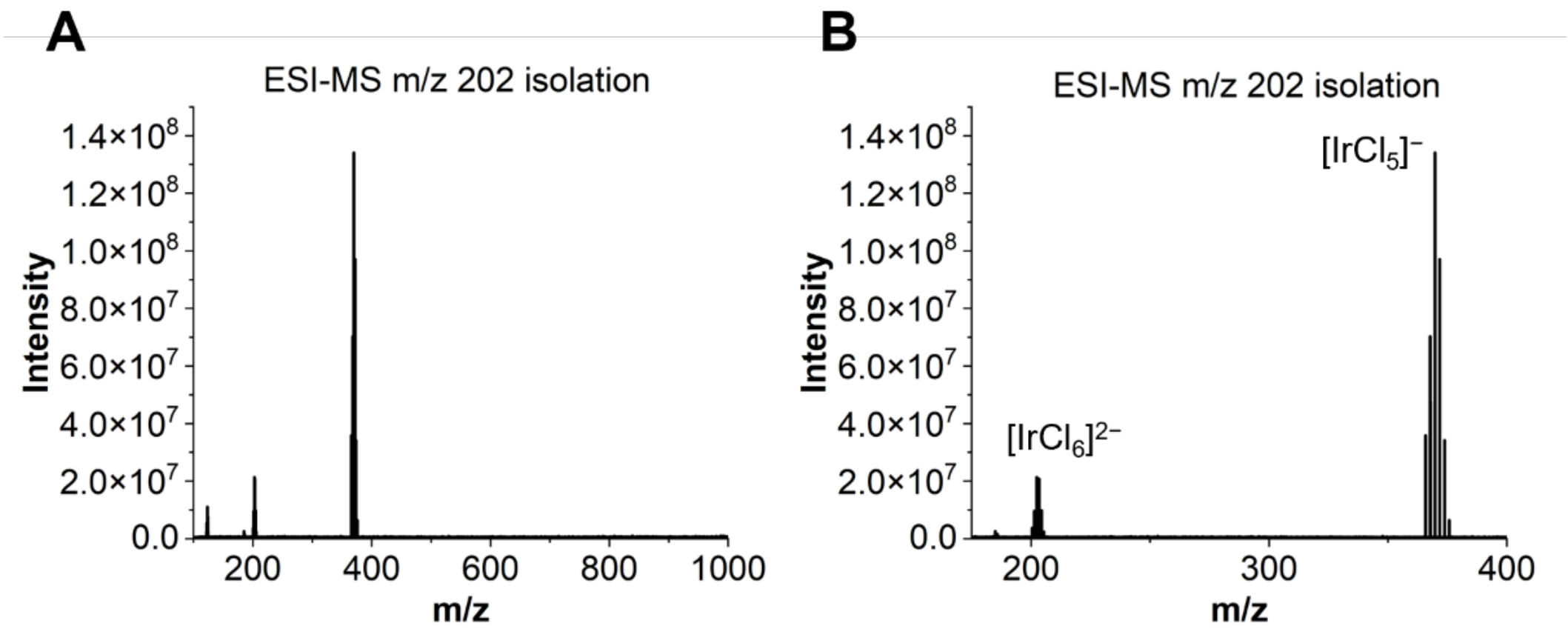

Figure S8. Quadrupolar isolation of *m/z* 202 $[IrCl_6]^{2-}$ peak resulting in fragmentation to *m/z* 369 $[IrCl_5]^-$. No collision voltage was applied – the instability of the doubly charged anion in the gas phase means that fragmentation occurred during ion transfer. **A** Full mass spectrum (m/z range 180 – 1000) acquired post isolation of *m/z* 202; **B** mass spectrum (*m/z* range 180 – 400) showing isotope patterns of $[IrCl_6]^{2-}$ and $[IrCl_5]^-$.

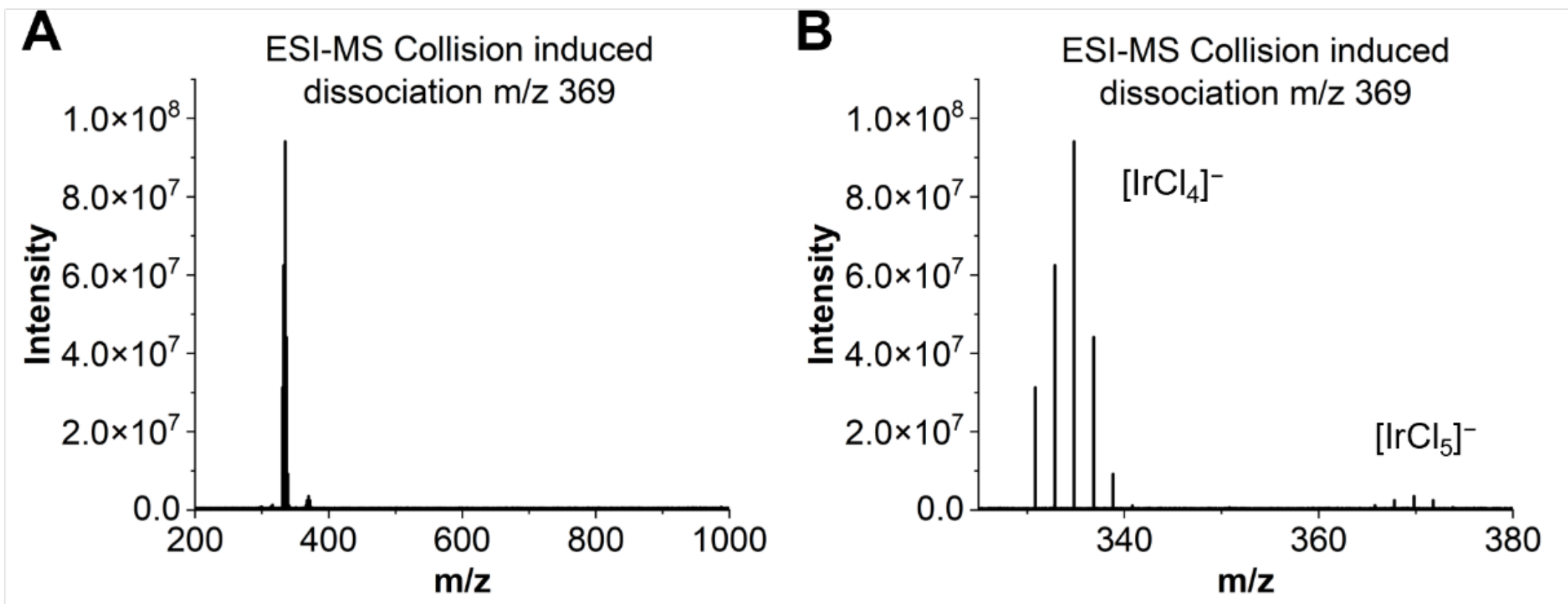

Figure S9. Mass spectrum acquired after the quadrupolar isolation of m/z 369 $[IrCl_5]^-$ followed by application of a 10 V collision energy resulting in fragmentation into *m/z* 334 $[IrCl_4]^-$. **A** *m/z* range 200-1000; **B** *m/z* range 325-280 showing isotope patterns of $[IrCl_5]^-$ and $[IrCl_4]^-$.

3.3. Analysis of post-refinery chloroiridate solution by nESI-MS at different pHs

3.3.1. Experimental protocol

The post-refinery chloroiridate solution was diluted to 1 mM [Ir] (12 µL in 12 mL H_2O) and basified with an aqueous solution of ammonia (35 %) to pH 8. The resulting solution was analysed after one hour by nano-electrospray ionisation mass spectrometry.

3.3.2. nESI-MS spectrum pH 8 t= 1 hour

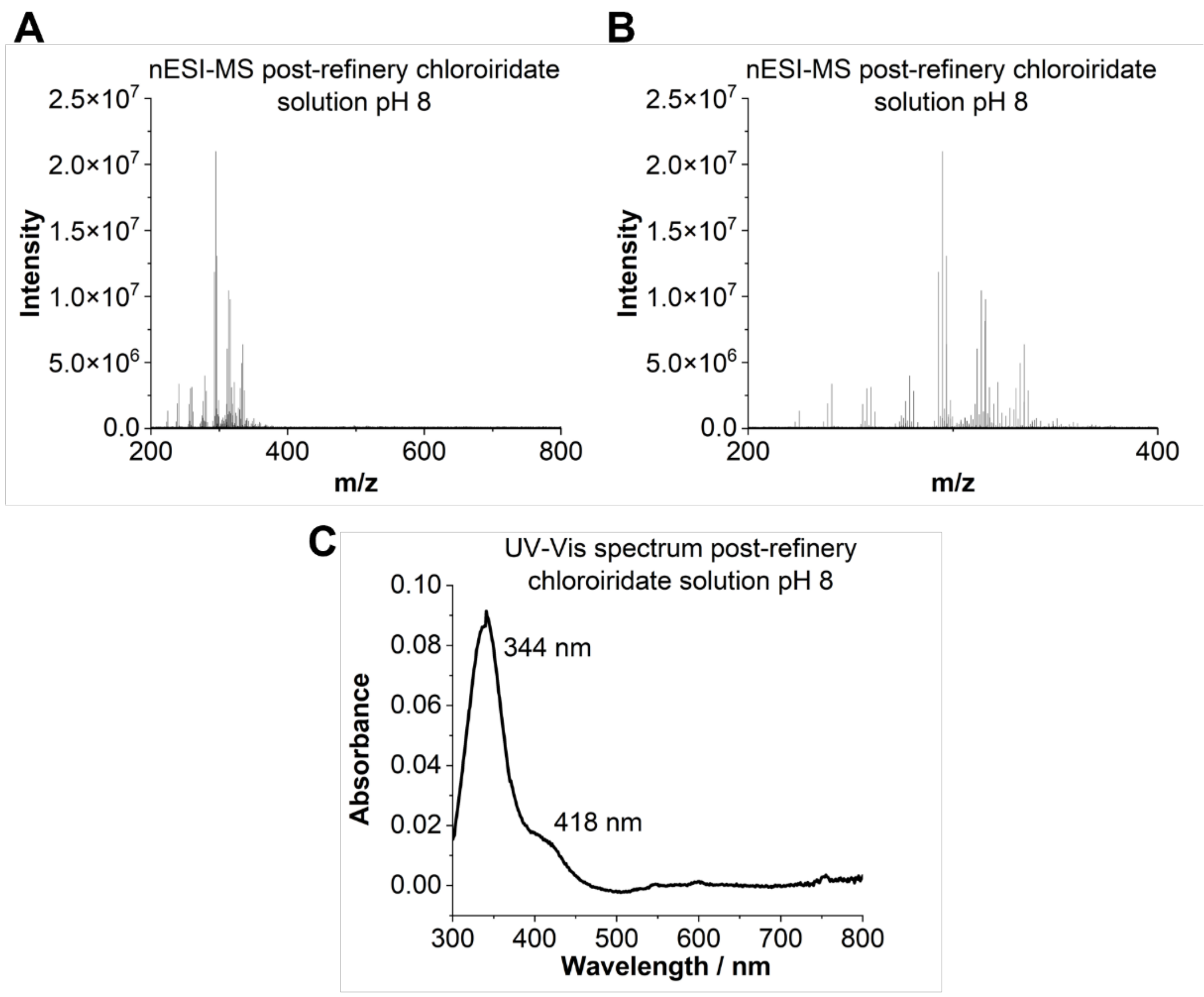

Figure S10. Mass spectra obtained by nESI-MS of crude refinery solution adjusted to pH 8 using aqueous ammonia. **A** m/z range 200-800. **B** m/z range 200-400 showing isotope patterns. **C** UV-Vis spectrum showing no remaining trace associated with $[IrCl_6]^{2-}$.

Major species identified in mass spectrum: $[Ir(OH)_5(OH_2) + K]^-$, $[Ir(OH)_6]^-$, $[Ir(OH)_4(OH_2)]^-$.

4.0. Synthesis and characterisation

4.1. $K_2[Ir(OH)_6]$

$Na_2[IrCl_6]$ (0.50 g, 1.1 mmol) was heated to 130 °C in KOH solution (2 M, 50 mL) with H_2O_2 (35 %, 0.5 mL) for 15 minutes. The resulting solution was left to sit at room temperature for three hours. Subsequently, this solution was heated to 110 °C until its volume halved, then left to slowly evaporate at room temperature over 36 hours resulting in red prismatic crystals of $K_2[Ir(OH)_6]$ (0.25 g, 60 %).

IR (ATR, neat) *v*/ cm^{-1} 3322 *v*(O–H), 1011 δ(O–H), 473 *v*(Ir–O)

λ_{max} (2 M KOH) 313 nm (ε = 2500 M^{-1} cm^{-1})

$E_{1/2}$ (2 M KOH) = − 0.44 V vs Ag/AgCl

ESI-MS (water): $[M + K]^-$ *m/z* 333.94355 (calc. $[Ir(OH)_6 + K]^-$ 333.94346)

Raman shift (neat) / cm^{-1} 284, 572, 3342

μ_{eff} (neat) 1.90 μ_B

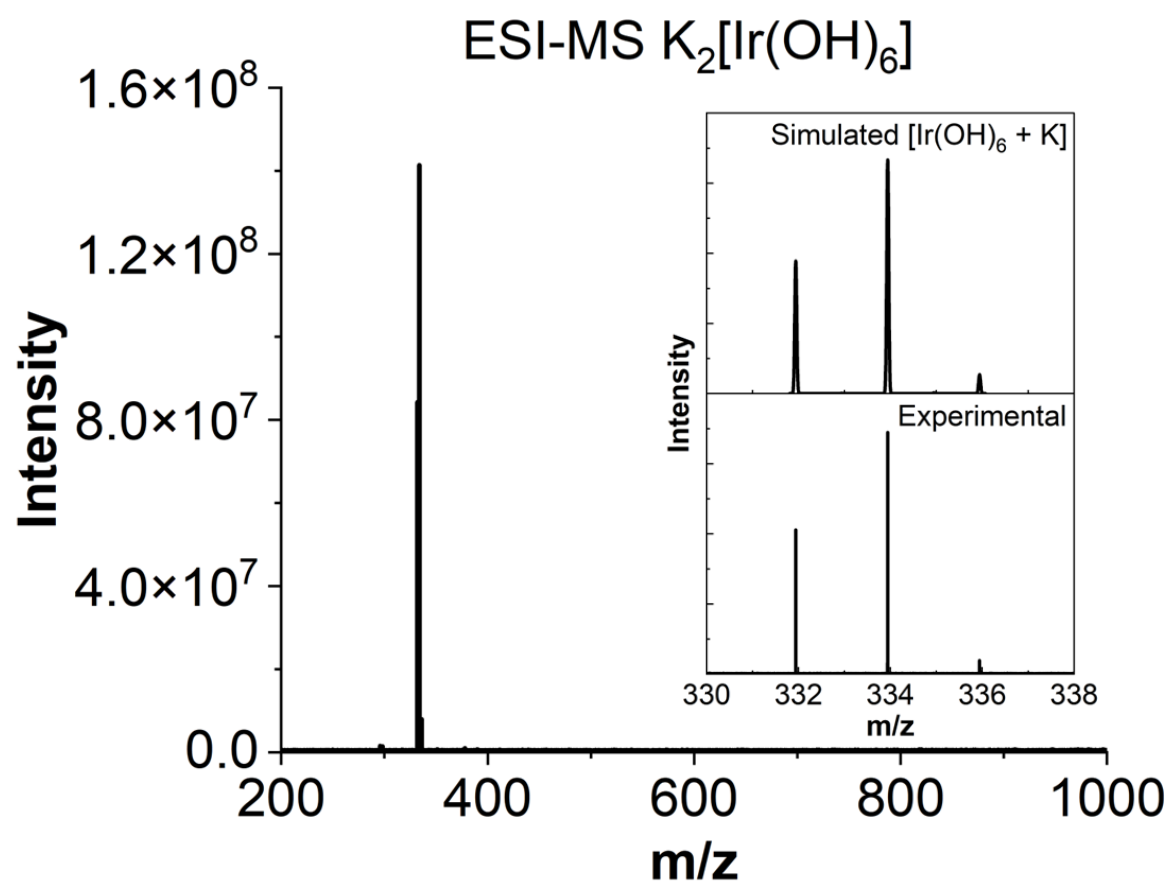

Figure S11. Negative mode ESI-MS spectrum of $K_2[Ir(OH)_6]$ (1 mg mL^{-1}) showing a single peak with m/z 333.94355 (calc. $[Ir(OH)_6 + K]^-$ 333.94346), matching simulated isotope pattern.

4.2. Ir(acac)$_3$ (acac = $C_5H_7O_2$)

$K_2[Ir(OH)_6]$ (52 mg, 0.14 mmol) was dissolved in water (3.5 mL) with acetylacetone (4 equiv., 0.54 mmol, 55 µL) under argon and heated to 50 °C for 10 days. The resulting water- insoluble yellow precipitate was separated from the crude reaction mixture by cannula filtration and dried under vacuum to give Ir(acac)$_3$ as a yellow solid (50 mg, 73 %).

^{1}H NMR (500 MHz, C_6D_6, 298 K) δ/ ppm 1.70 (s, 6H), 5.22 (s, 1H)

^{13}C NMR (150.91 MHz, C_6D_6, 298 K) δ/ ppm 26.1, 102.0, 185.9

λ_{max} / nm (acetonitrile) 261 (ε = 1900 M^{-1} cm^{-1}), 312 (ε = 782 M^{-1} cm^{-1}), 425 (ε = 63 M^{-1} cm^{-1})

IR (ATR, neat) *v* / cm^{-1} 1548, 1517s, 1431sh, 1382, 1366, 1273, 1201, 1016, 932, 779, 650

ESI-MS (+) (EtOH): $[M + K]^+$ m/z 529.07068 (calc. $[Ir(C_5H_7O_2)_3 + K]^+$ 529.060995)

Elemental analysis % C 36.5 (calc. 36.8) % H 4.3 (calc. 4.3)

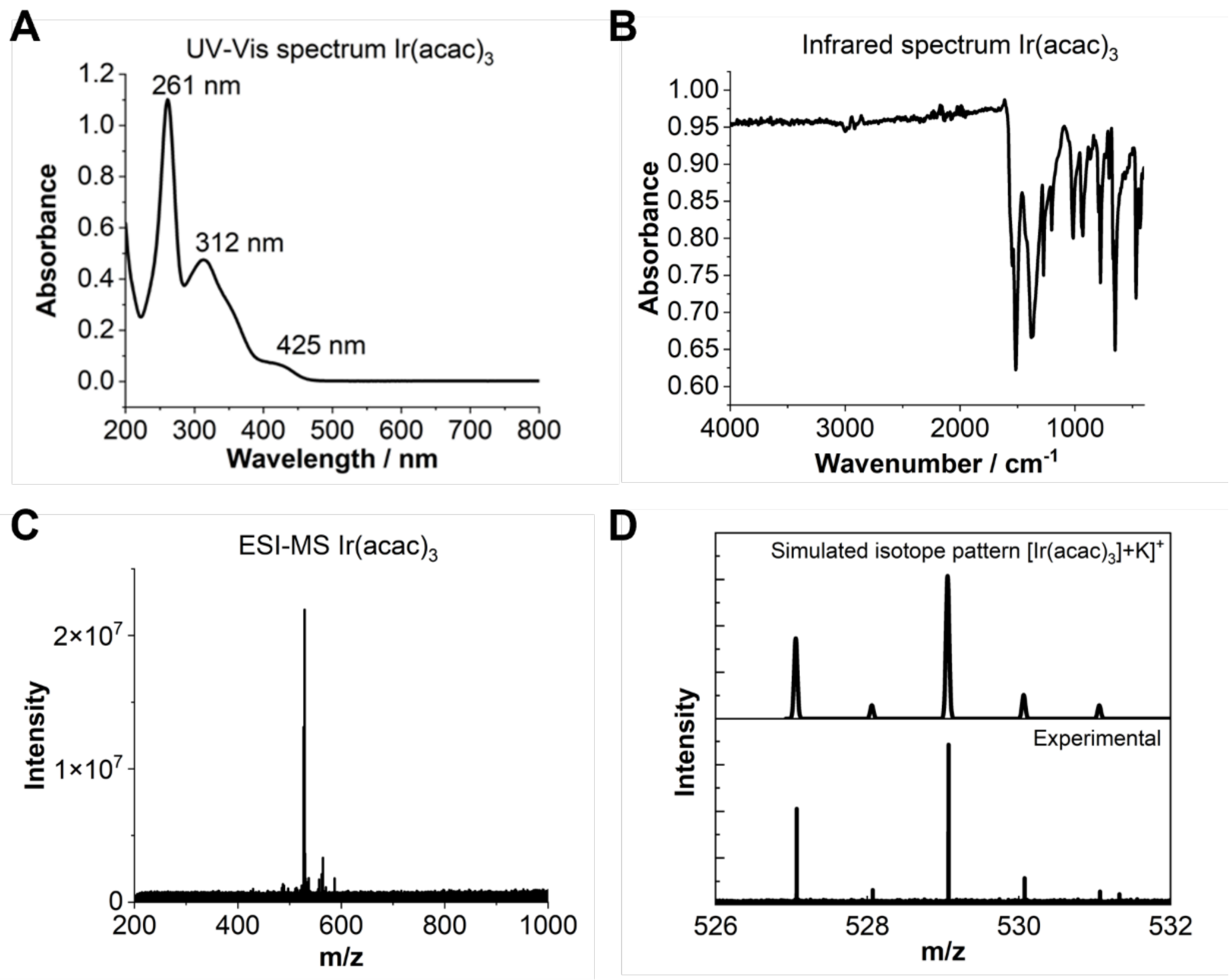

Figure S12. Characterisation data Ir(acac)$_3$ **A** UV-Vis spectrum Ir(acac)$_3$ in MeCN: λ_{max} / nm (acetonitrile) 261 (ε = 1900 M^{-1} cm^{-1}), 312 (ε = 782 M^{-1} cm^{-1}), 425 (ε = 63 M^{-1} cm^{-1}) matching literature reported values[4]; **B** Infrared spectrum (neat) Ir(acac)$_3$ matching literature values[5, 6]; **C** Mass spectrum (ESI +) of Ir(acac)$_3$ in ethanol (100 µg mL^{-1}) showing major species $[Ir(acac)_3 + K]^+$ m/z 529.07069 (calc. 529.06087); **D** Simulated isotope pattern $[Ir(acac)_3+K]^+$ matching experimental data.

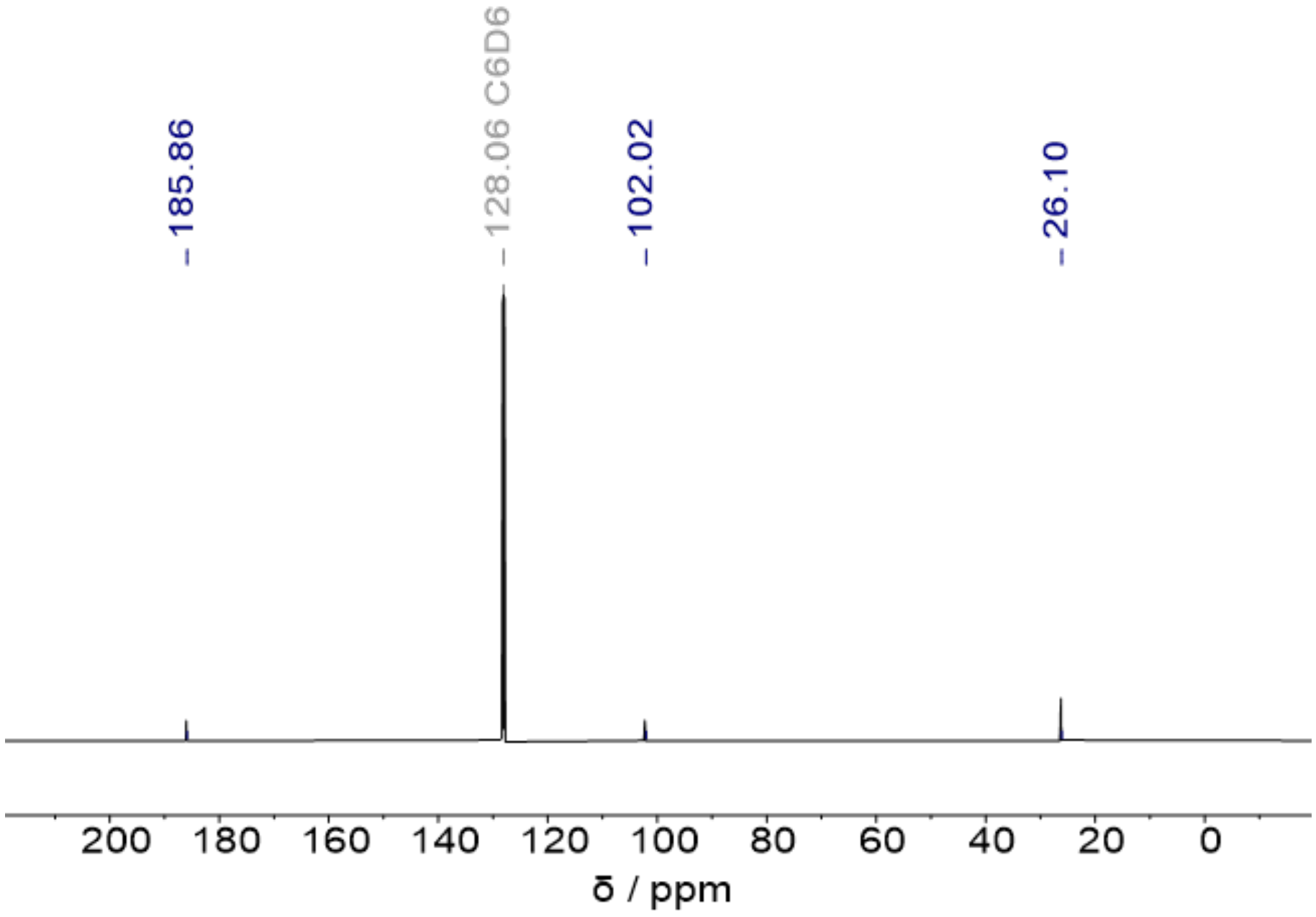

Figure S13. ^{13}C NMR spectrum (150.91 MHz, C_6D_6, 298 K, Ar) Ir(acac)$_3$, in agreement with expected resonances for an M(acac)$_3$ complex.[7] Note: solubility limit of Ir(acac)$_3$ in benzene-d_6 reached.

4.3. $K[Ir(C_2O_4)_2(H_2O)_2]$ (oxalate = C_2O_4)

$K_2[Ir(OH)_6]$ (30 mg, 0.081 mmol) and oxalic acid (21 equiv., 1.75 mmol, 158 mg) were heated to 50 °C for five days in water under argon. A colour change from purple to yellow, via green, was observed. Tetra-*n*-butylammonium hydrogen sulfate (100 mg, 0.295 mmol) was added to the crude reaction mixture, and the product extracted with nitromethane (3 x 5 mL). The solvent was removed *in vacuo* prior to the addition of potassium acetate (52 mg, 0.52 mmol) in ethanol (10 mL). A fine yellow precipitate was separated by filtration, washed with acetonitrile and dried under vacuum to yield $K[Ir(C_2O_4)_2(H_2O)_2]$ as a pale-yellow powder (21 mg, 62 %).

IR (ATR, neat) v / cm^{-1} 3322 br, 1588 s, 1382, 1305 s, 1113 s, 813, 768, 719, 617, 526.

λ_{max} (water) 286 nm

$E_{1/2}$ (water) + 0.92 V vs Ag/AgCl

ESI-MS (water): $[M – 2H_2O]^-$ m/z 368.90378 (calc. $[Ir(C_2O_4)_2]^-$ m/z 368.92279)

^{13}C NMR (125.8 MHz, D_2O, 298 K) δ/ ppm 171.3

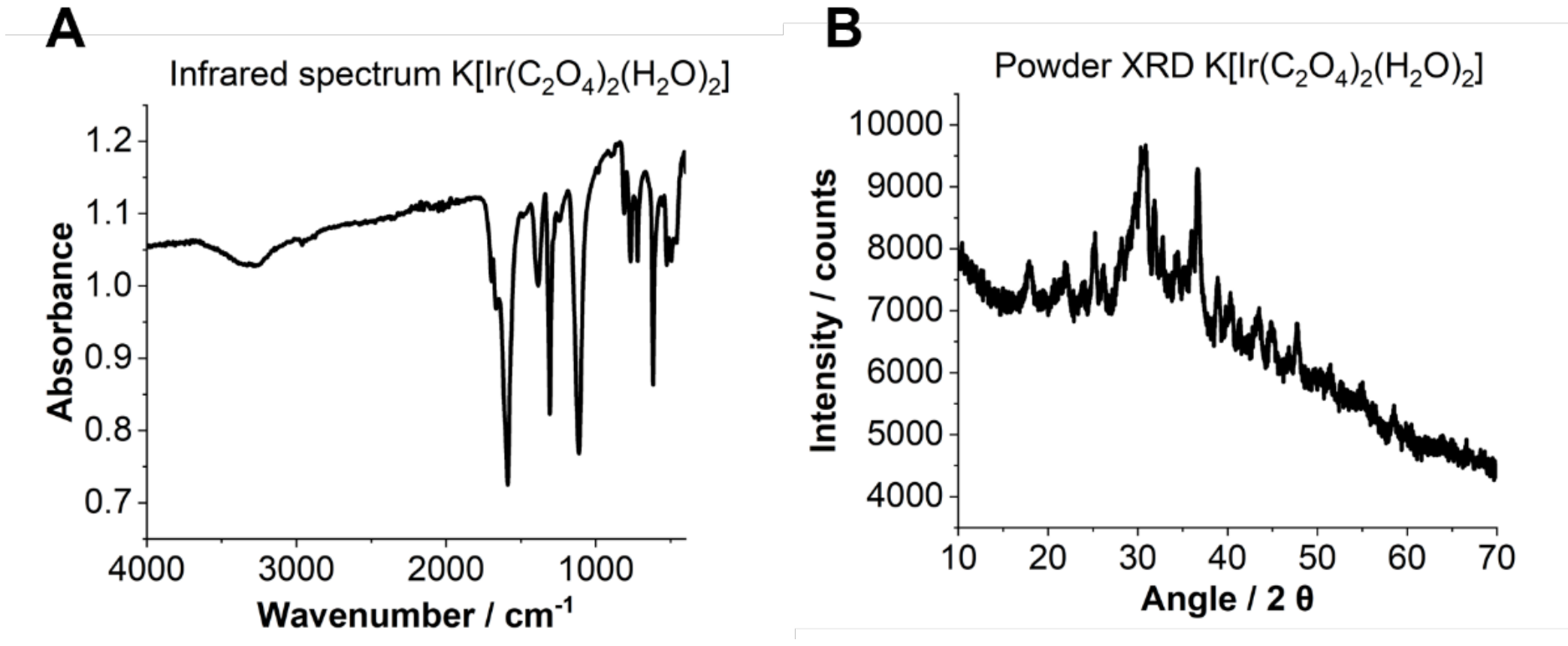

Figure S14. Solid-state characterisation $K[Ir(C_2O_4)_2(H_2O)_2]$ prior to recrystallisation. **A** Infrared spectrum (neat) $K[Ir(C_2O_4)_2(H_2O)_2]$; **B** Powder XRD showing low degree of crystallinity of the sample.

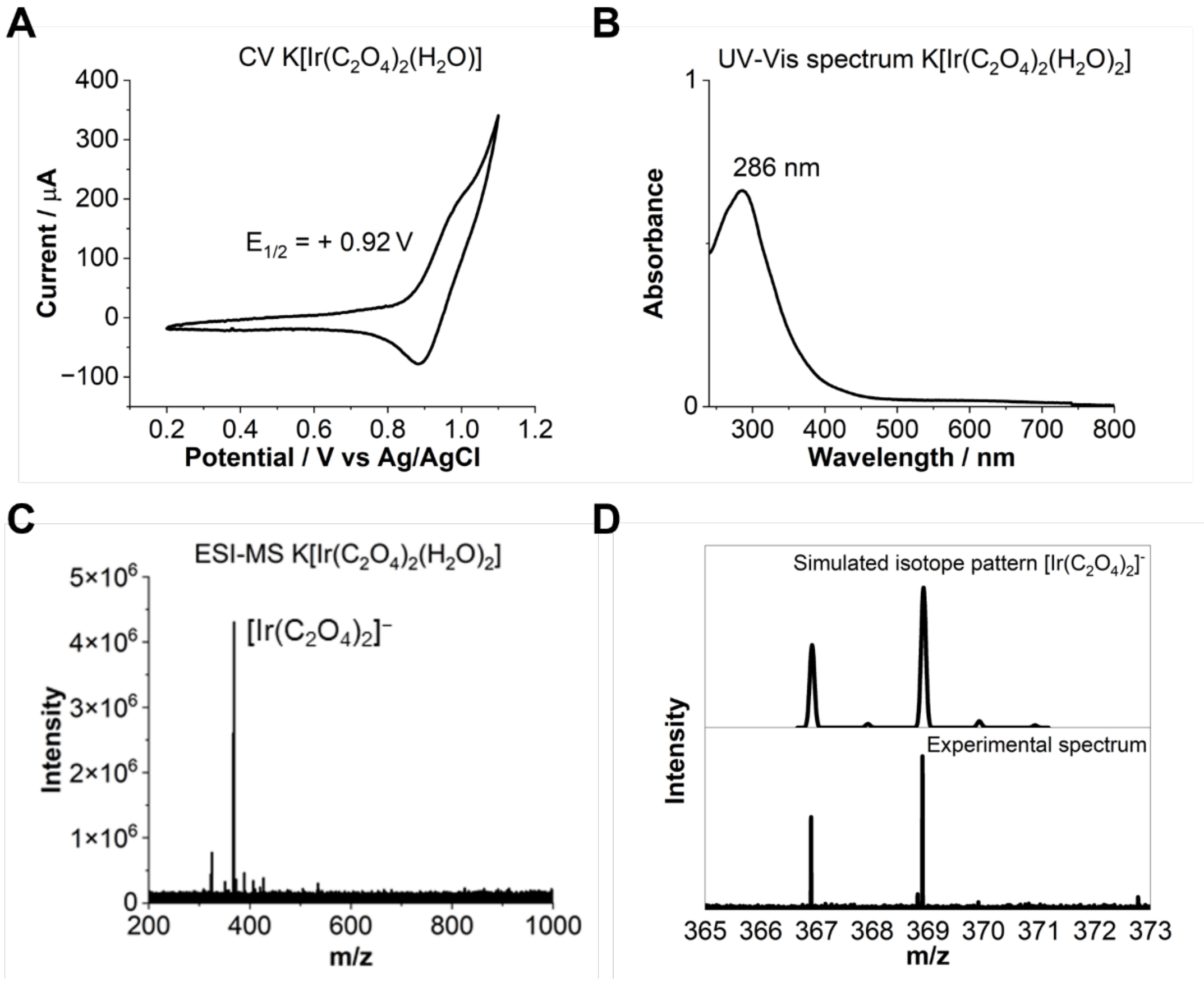

Figure S15. Solution-state characterisation $K[Ir(C_2O_4)_2(H_2O)_2]$. **A** Cyclic voltammetry in water. **B** UV-Vis spectroscopy in water. **C** ESI-MS spectrum in water, measured immediately post dissolution. **D** Simulated isotope pattern $[Ir(C_2O_4)_2]^-$ matching experimental spectrum.

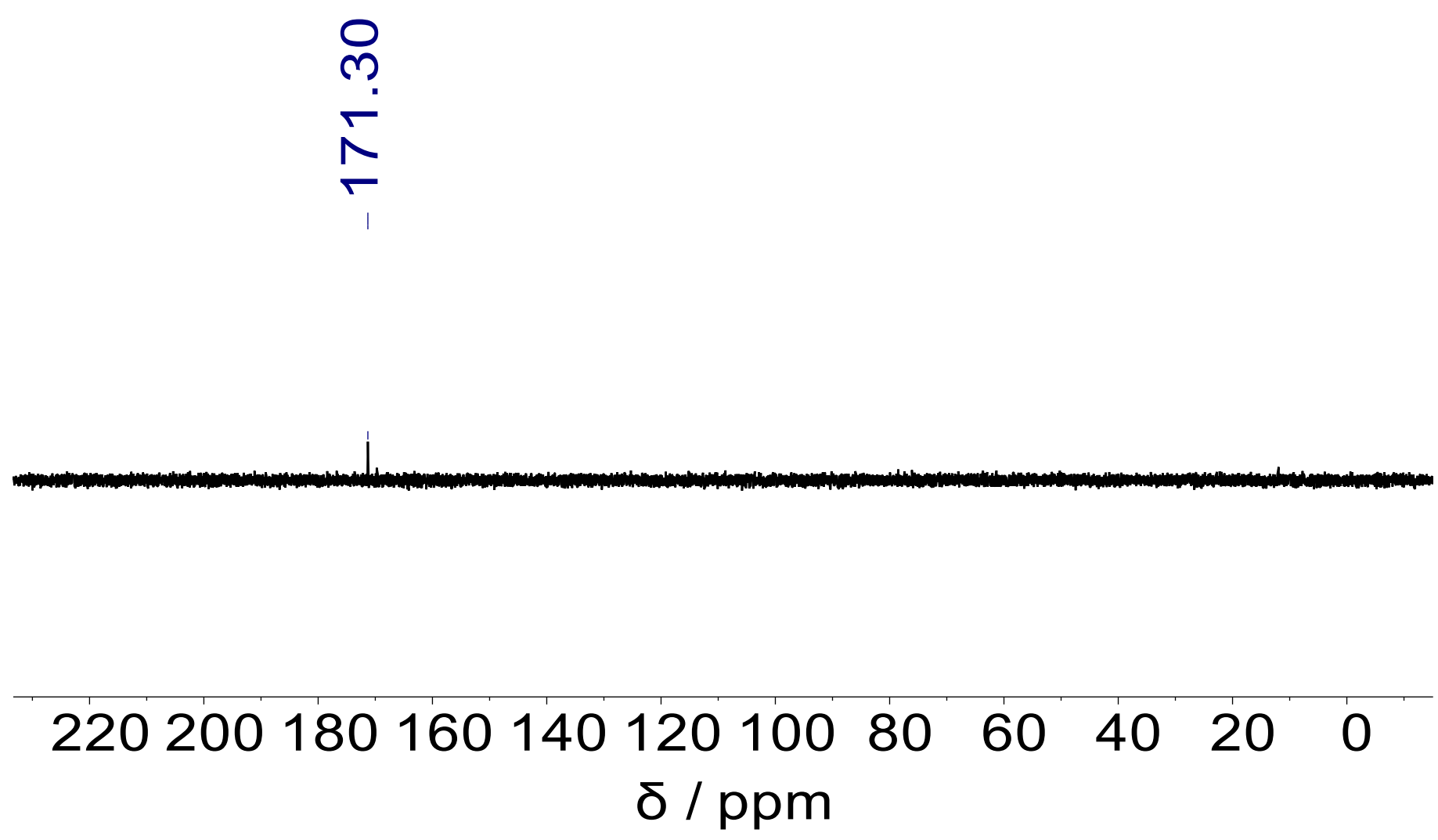

Figure S16. ^{13}C NMR spectrum (125.8 MHz, D_2O, 298 K) crude product $K[Ir(C_2O_4)_2(H_2O)_2]$.

Through selective recrystallisation using different antisolvents, *cis*- and *trans*-$K[Ir(C_2O_4)_2(H_2O)_2]$ can be isolated.

4.3.1. *cis*-K[Ir(oxalate)$_2$(H_2O)$_2$]

The slow evaporation under argon of a layered solution of crude $K[Ir(C_2O_4)_2(H_2O)_2]$ in water with tetrahydrofuran results in the formation of yellow crystals suitable for analysis by single-crystal XRD.

IR (ATR, neat) *v* / cm^{-1} 3299 br *v*(O–H), 1588 s v_a(C=O), 1378 v_s(C–O) (trans to –OH$_2$), 1305 s v_s(C–O) (trans to –O–C=O), 1113 δ(Ir–OH$_2$), 813 δ(O–C=O) (trans to –OH$_2$), 768 δ(O–C=O) (trans to –O–C=O), 617, 522 *v*(Ir–OH$_2$), 469 *v*(Ir–O–C=O).

^{13}C NMR (150.91 MHz, D_2O, 298 K) δ/ ppm 171.5, 171.1

λ_{max} (water) 286 nm (ε = 2300 M^{-1} cm^{-1})

$E_{1/2}$ (water) + 0.92 V vs Ag/AgCl

ESI-MS (water) [M – 2H$_2$O]$^-$ m/z 368.90377 (calc. $[Ir(C_2O_4)_2]^-$ m/z 368.92279)

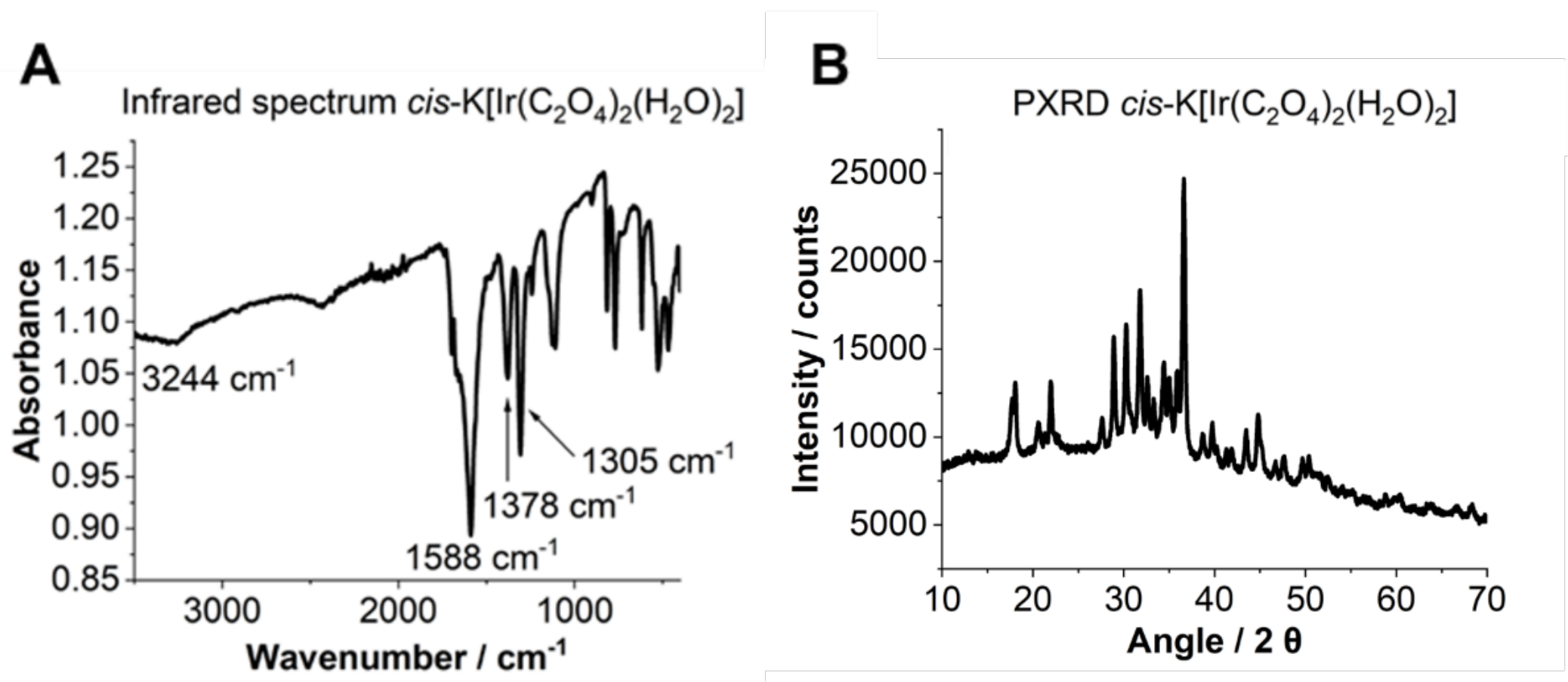

Figure S17. Solid-state characterisation *cis*-K[Ir(C_2O_4)$_2$(H_2O)$_2$]. **A** Infrared spectrum; **B** Powder XRD.

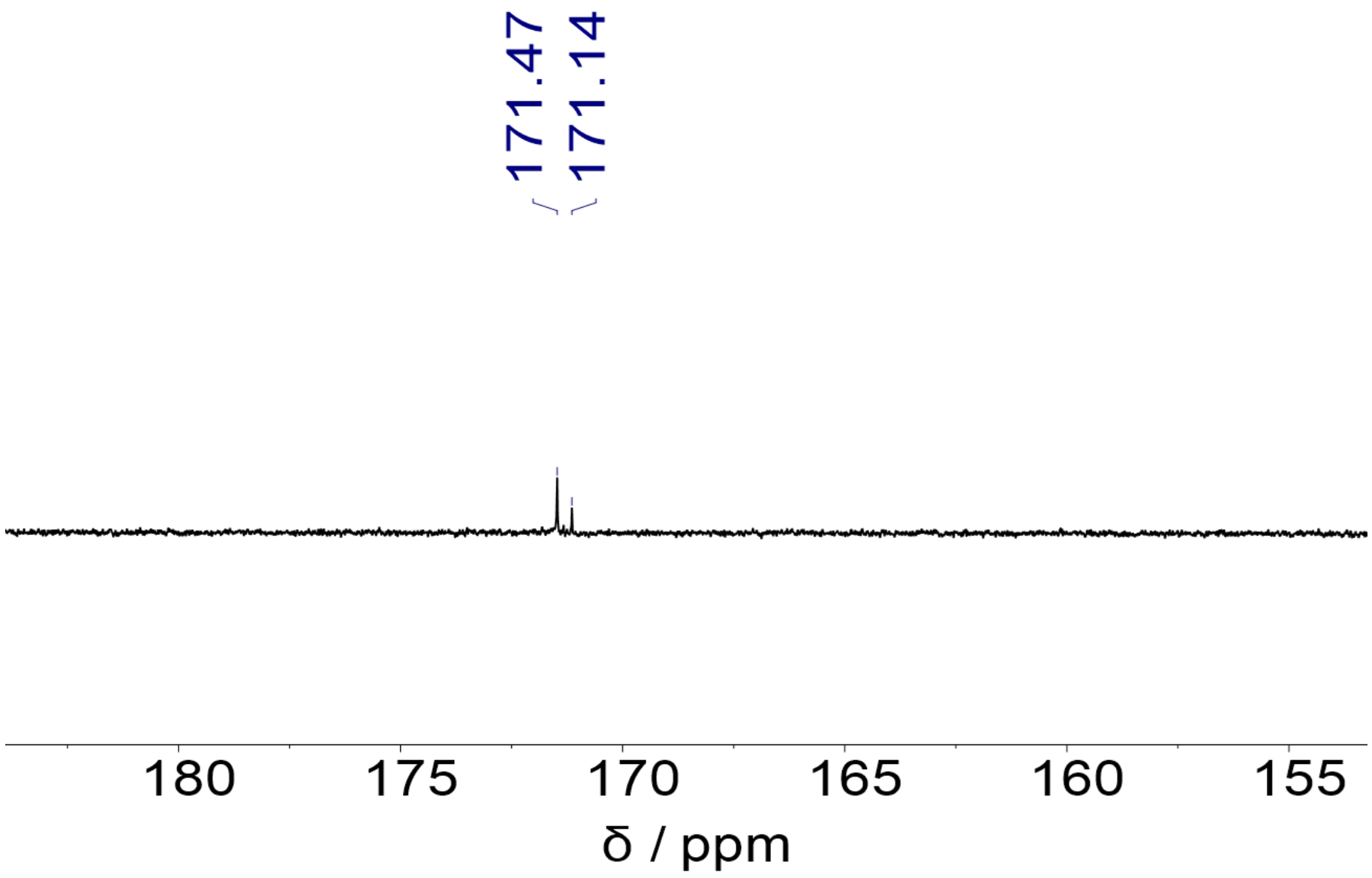

Figure S18. ^{13}C NMR spectrum (125.8 MHz, D_2O, 298 K) *cis*-K[Ir(C_2O_4)$_2$(H_2O)$_2$].

4.3.2. trans-K[Ir(C_2O_4)$_2$(H_2O)$_2$]

The slow evaporation under argon of a layered solution of K[Ir(C_2O_4)$_2$(H_2O)$_2$] in water with isopropylalcohol results in the formation of yellow crystals suitable for analysis by single-crystal XRD.

IR (ATR, neat) *v* / cm^{-1} 3334 br *v*(O–H), 1588 s v_a(C=O), 1305 s v_s(C–O) (trans to –O–C=O), 1113 δ(Ir–OH_2), 768 δ(O–C=O) (trans to –O–C=O), 522 *v*(Ir–OH_2), 469 *v*(Ir–O–C=O)

^{13}C NMR (150.91 MHz, D_2O, 298 K) δ/ ppm 171.2

λ_{max} (water) 286 nm (ε = 2300 M^{-1} cm^{-1})

$E_{1/2}$ (water) + 0.92 V vs Ag/AgCl

ESI-MS (water): [M – 2H_2O]$^-$ m/z 368.90378 (calc. [Ir(C_2O_4)$_2$]$^-$ m/z 368.92279)

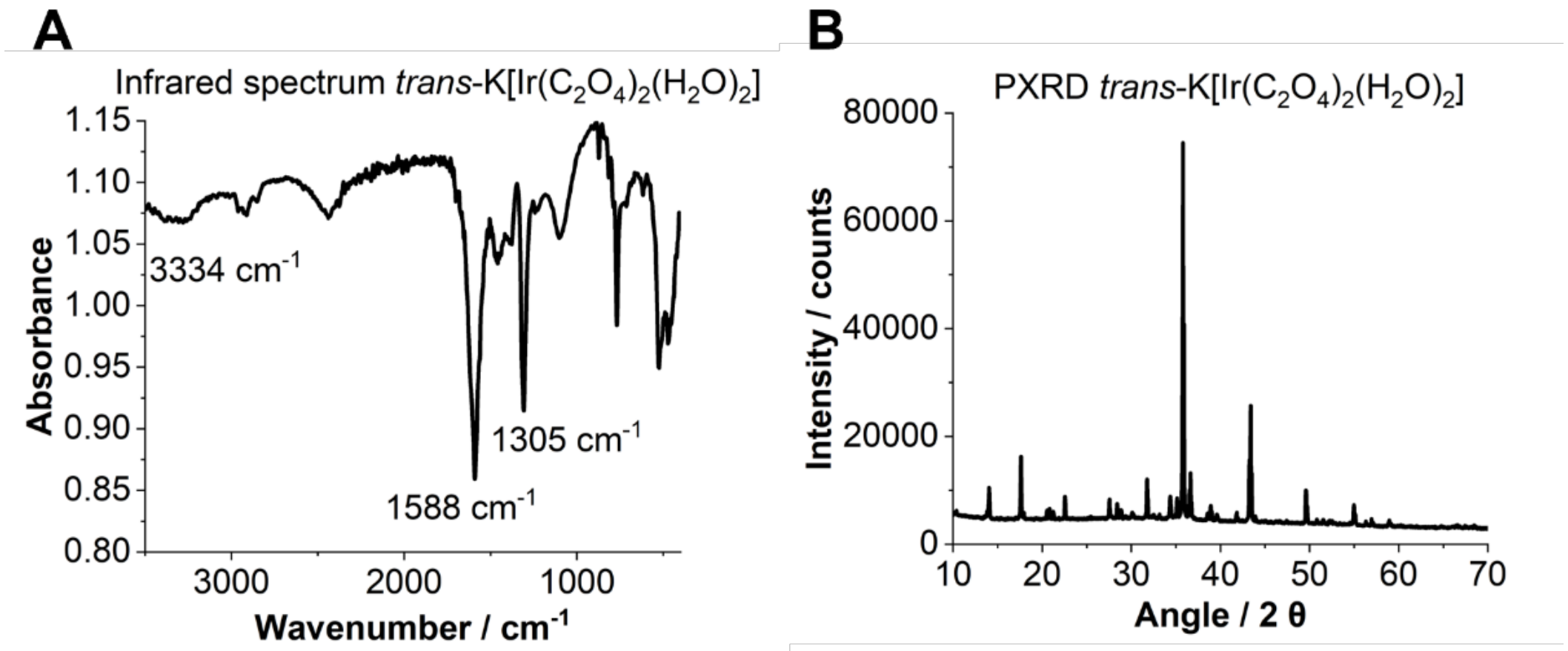

Figure S19. Solid-state characterisation *trans*-K[Ir(C_2O_4)$_2$(H_2O)$_2$]. **A** Infrared spectrum; **B** Powder XRD.

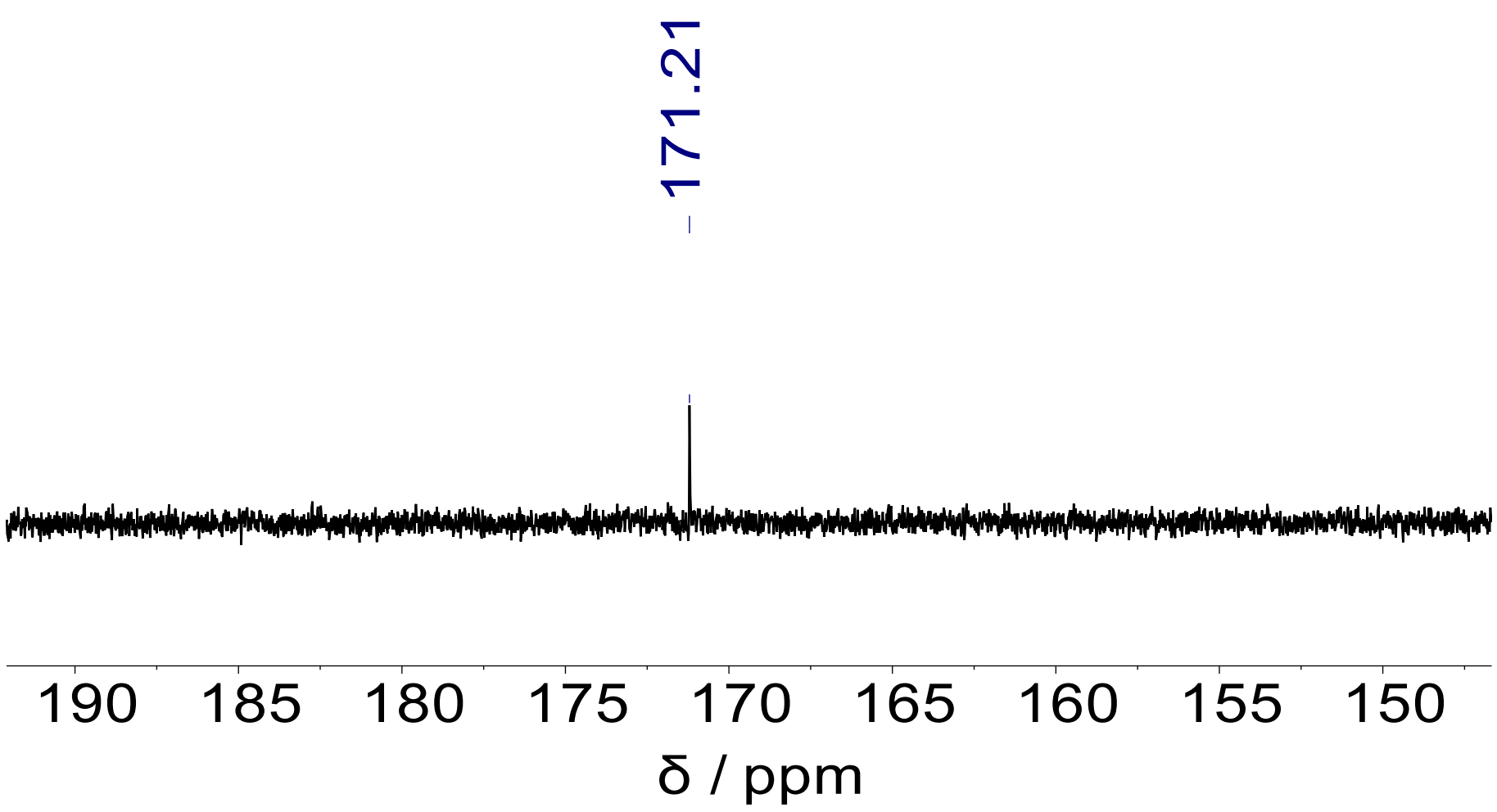

Figure S20. ^{13}C NMR spectrum (125.8 MHz, *D*$_2$O, 298 K) *trans*-K[Ir(C_2O_4)$_2$(H_2O)$_2$].

4.4. K_3[Ir(citrate)$_2$] (citrate = $C_6H_5O_7$)

K_2[Ir(OH)$_6$] (30 mg, 0.081 mmol) and citric acid (2 equiv., 0.162 mmol, 36 mg) were stirred in water (3.5 mL) under argon for 72 hours at 50 °C. A cloudy yellow solution formed. The solvent was removed *in vacuo* and the resulting yellow oil washed with methanol under argon. The resulting yellow solid was dried under vacuum to yield K_3[Ir($C_6H_5O_7$)$_2$] as a yellow-brown powder (26 mg, 46 %).

^{1}H NMR (600 MHz, *D*$_2$O, 298 K) δ/ ppm 2.88 (d, 1H, ***J*** = 15.4 Hz), 2.77 (d, 1H, ***J*** = 15.5 Hz)

^{13}C NMR (150.91 MHz, *D*$_2$O, 298 K) δ/ ppm 178.9, 175.1, 74.1, 43.9

IR (ATR, neat) v / cm^{-1} 2906 br (O–H), 1696 sh, 1587 s (C=O stretch), 1368 (C–O stretch), 1305, 1239, 1191

$E_{1/2}$ (H_2O) + 0.35 V vs Ag/AgCl

ESI-MS (−) (H_2O): [M]$^{2-}$ *m/z* 285.49759 (calc. [Ir($C_6H_5O_7$)$_2$]$^{2-}$ 285.48557 u).

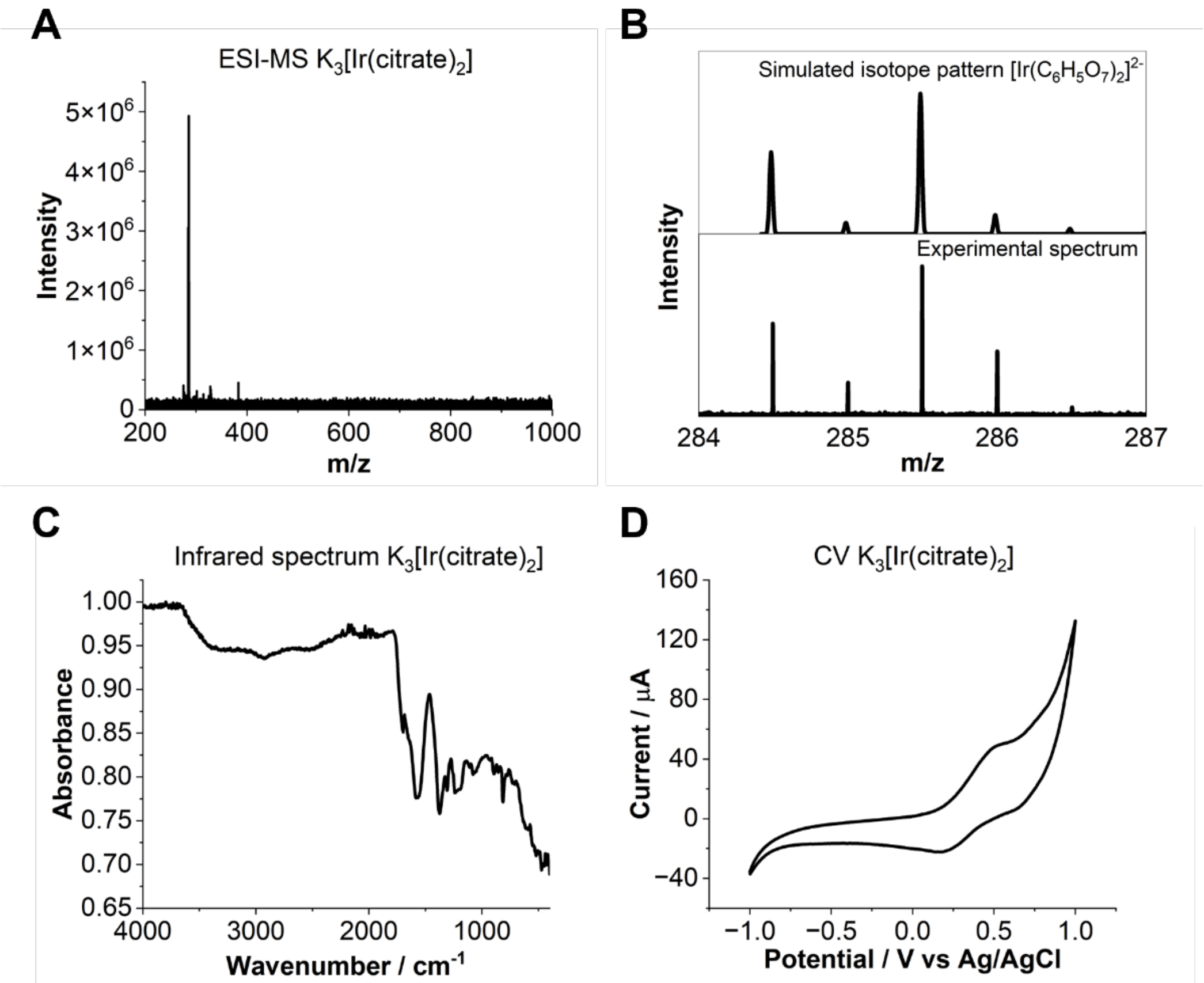

Figure S21. The characterisation of $K_3[Ir(C_6H_5O_7)_2]$: **A** ESI-MS showing major species $[Ir(C_6H_5O_7)_2]^{2-}$; **B** Simulated isotope pattern $[Ir(C_6H_5O_7)_2]^{2-}$ matching experimental spectrum; **C** IR spectrum (ATR, neat) showing two absorbances in $\nu(COO^-)$ region; **D** SPE-CV of $K_3[Ir(C_6H_5O_7)_2]$ in water with $E_{1/2}$ = + 0.35 V vs Ag/AgCl.

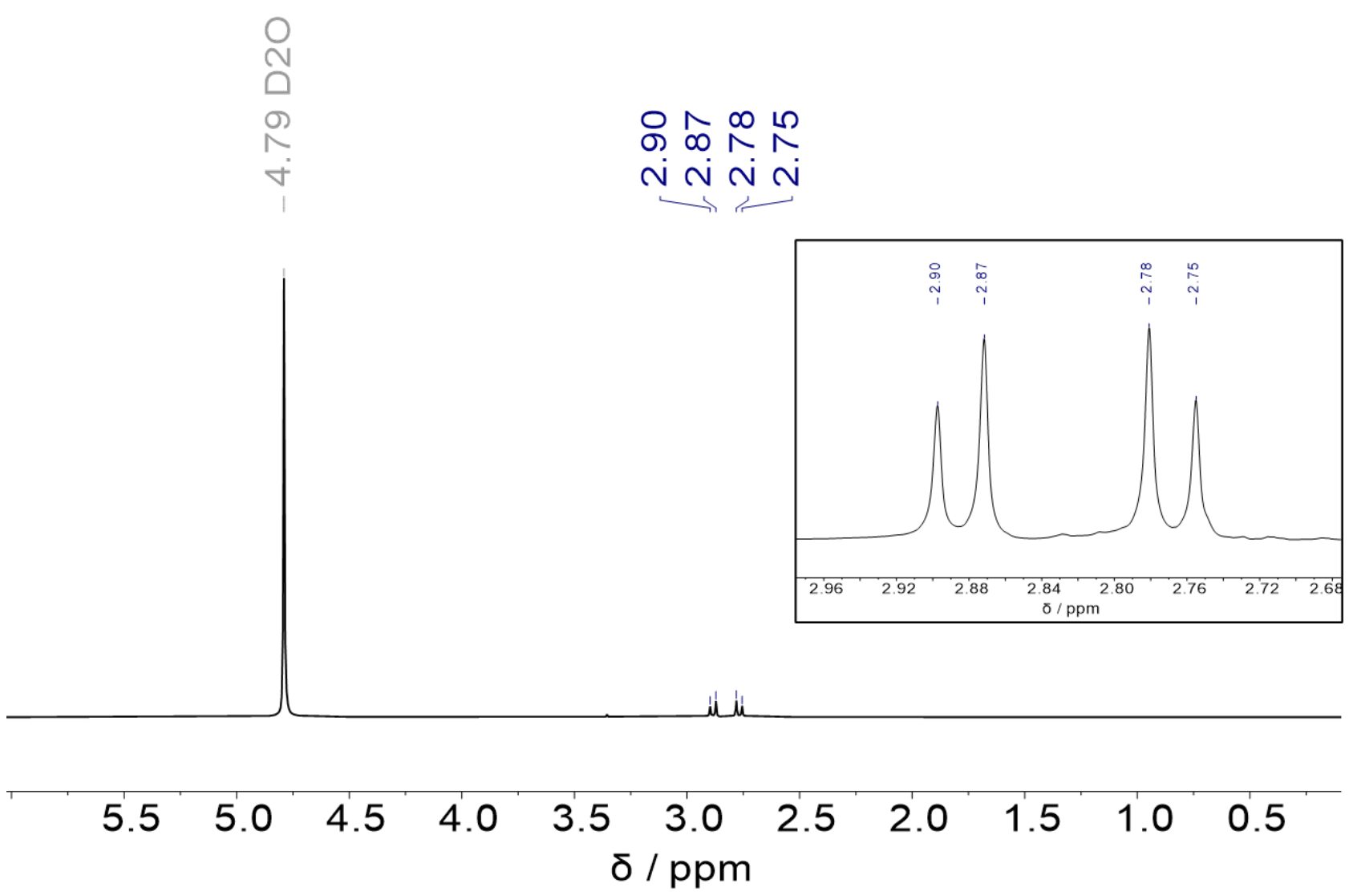

Figure S22. ^1H NMR spectrum (600 MHz, D_2O, Ar, 298 K) $K_3[Ir(C_6H_5O_7)_2]$ showing characteristic roofing doublets of citric acid, shifted upfield from free citric acid by 0.1 ppm). Note partial solubility in water.

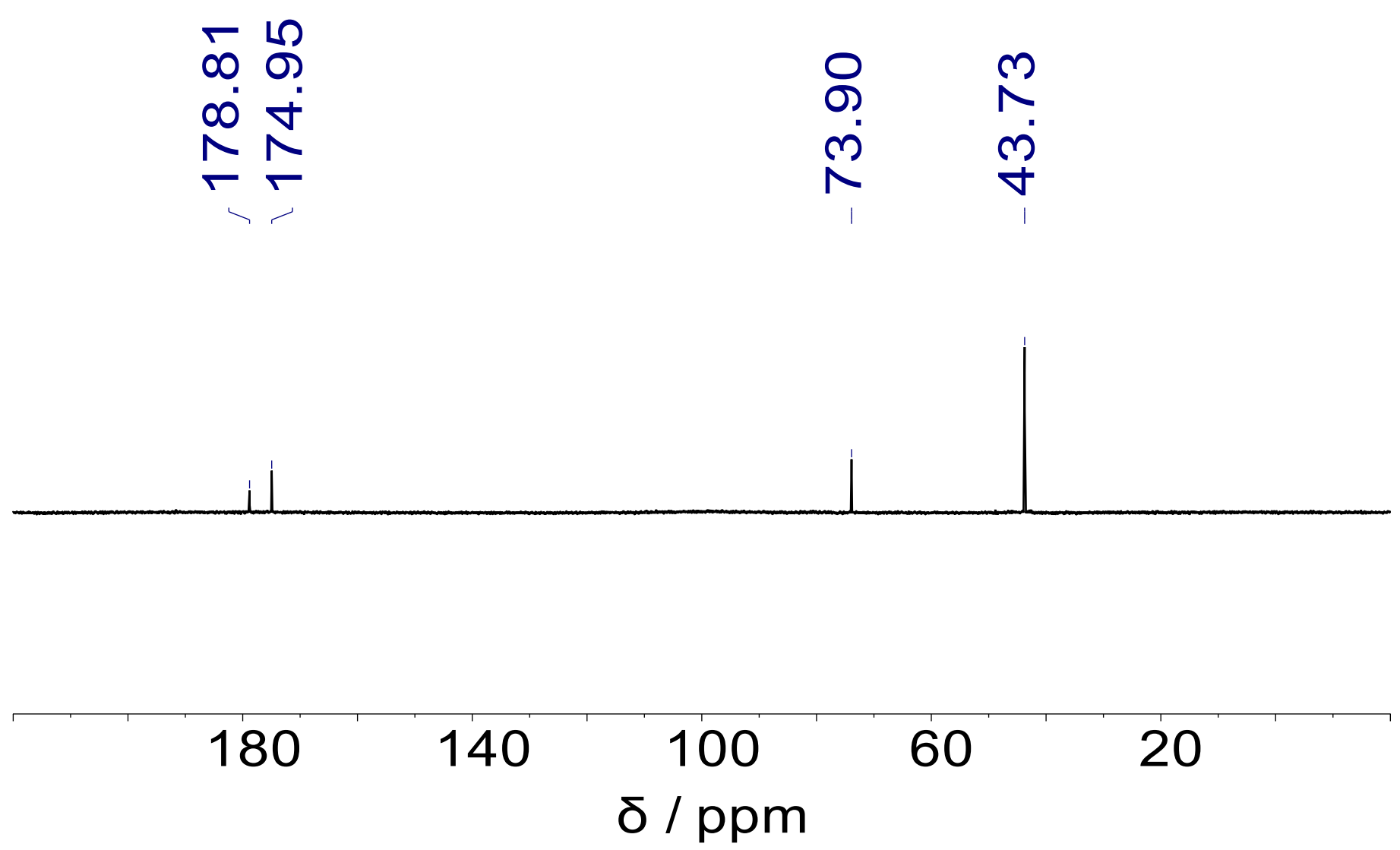

Figure S23. ^{13}C NMR spectrum (150.91 MHz, *D*$_2$O, 298 K) $K_3[Ir(C_6H_5O_7)_2]$. Downfield shift observed relative to free citric acid by approximately 2 ppm. Complex has partial solubility in water.

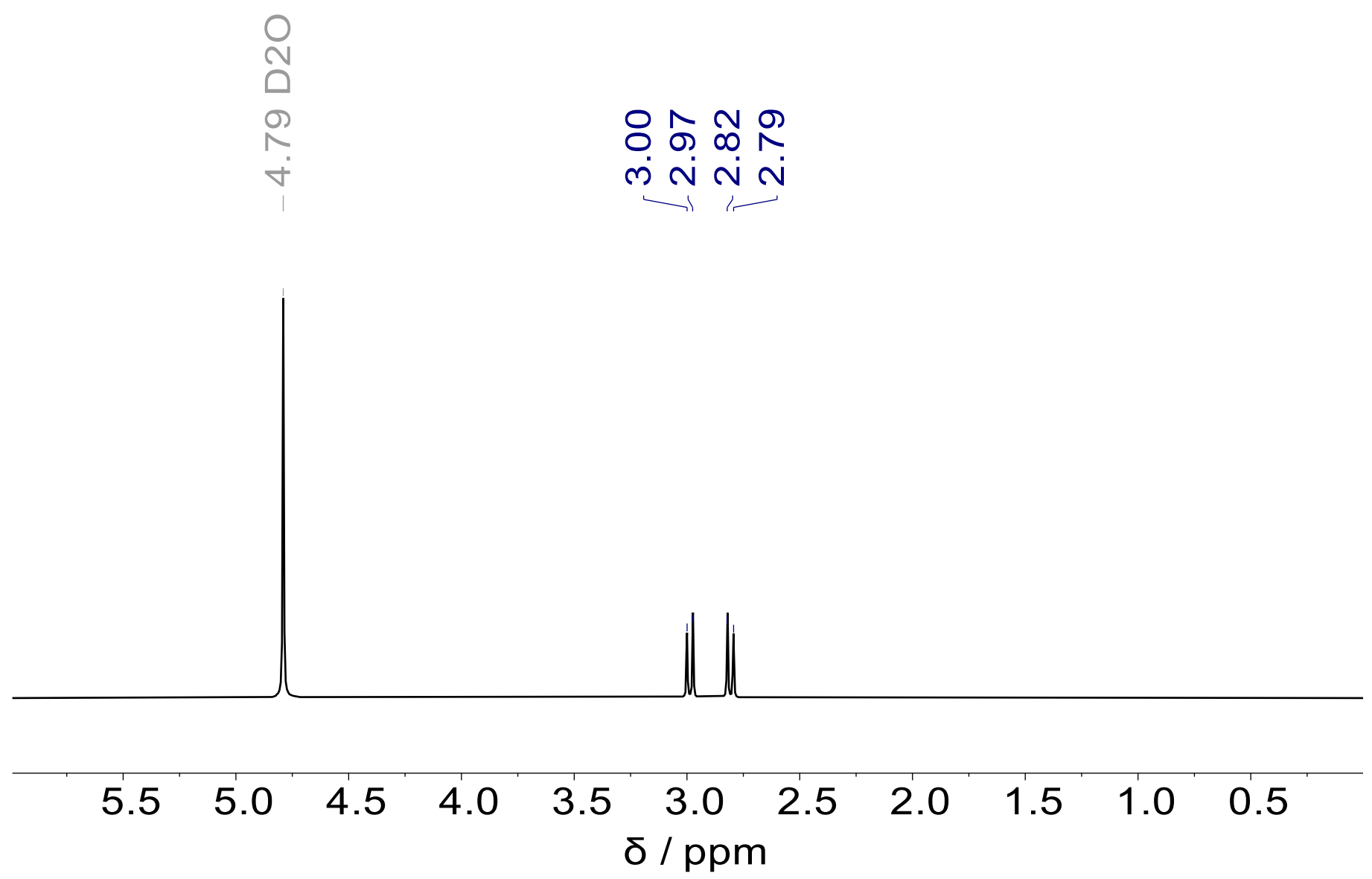

Figure S24. ^{1}H NMR spectrum (600 MHz, *D*$_2$O, Ar, 298 K) citric acid showing characteristic roofing doublet.

4.5. [NBu_4]$_2$[Ir_2Cl_{10}]

4.5.1. [NBu_4]$_2$[$IrCl_6$]

[NBu_4]$_2$[$IrCl_6$] was synthesised by a previously reported procedure.[8] NBu_4Br (600 mg, 1.86 mmol) was dissolved in a HCl solution (5 mL, 1 M) and slowly added to a solution of Na_2[$IrCl_6$] (200 mg) in HCl (15 mL, 1 M). The resulting mixture was stirred in air at 25 ^{o}C for 10 minutes. A brown precipitate was separated by filtration, washed with cold diethyl ether and dried under vacuum to yield [NBu_4]$_2$[$IrCl_6$] a dark brown powder (308 mg, 78 %).

IR ν_{max}/ cm^{-1} (neat) 2959 s, 2932, 2871, 1476 s, 1380, 1152, 1029, 883, 740

Note: Ir(IV)–Cl stretches are typically observed in the 300-350 nm range,[9] below the range of the IR spectrometer utilised (400 – 4000 cm^{-1}). Typical peaks associated with [NBu_4]$^+$ observed (cm^{-1}): 2959 s, 2932, 2871, 1476, 1379, 1152, 1029, 885 and 746, matching literature values.[8]

$E_{1/2}$ + 0.72 V vs Ag/AgCl (MeCN, 0.1 M NBu_4PF_6)

λ_{max} / nm (MeOH) 305, 417, 435, 490, 589

LDI-MS (−) [M−2Cl]$^-$ *m/z* 332.81655 (calc. [$IrCl_4$]$^-$ *m/z* 332.83660 u), [M−2Cl + 2H]$^-$ *m/z* 334.81157 (calc [[$IrCl_4$] + 2 H]$^-$ *m/z* 334.85255 u)

LDI-MS (+) [M]$^+$ *m/z* 242.26575 (calc. [NBu_4]$^+$ 242.28532 u)

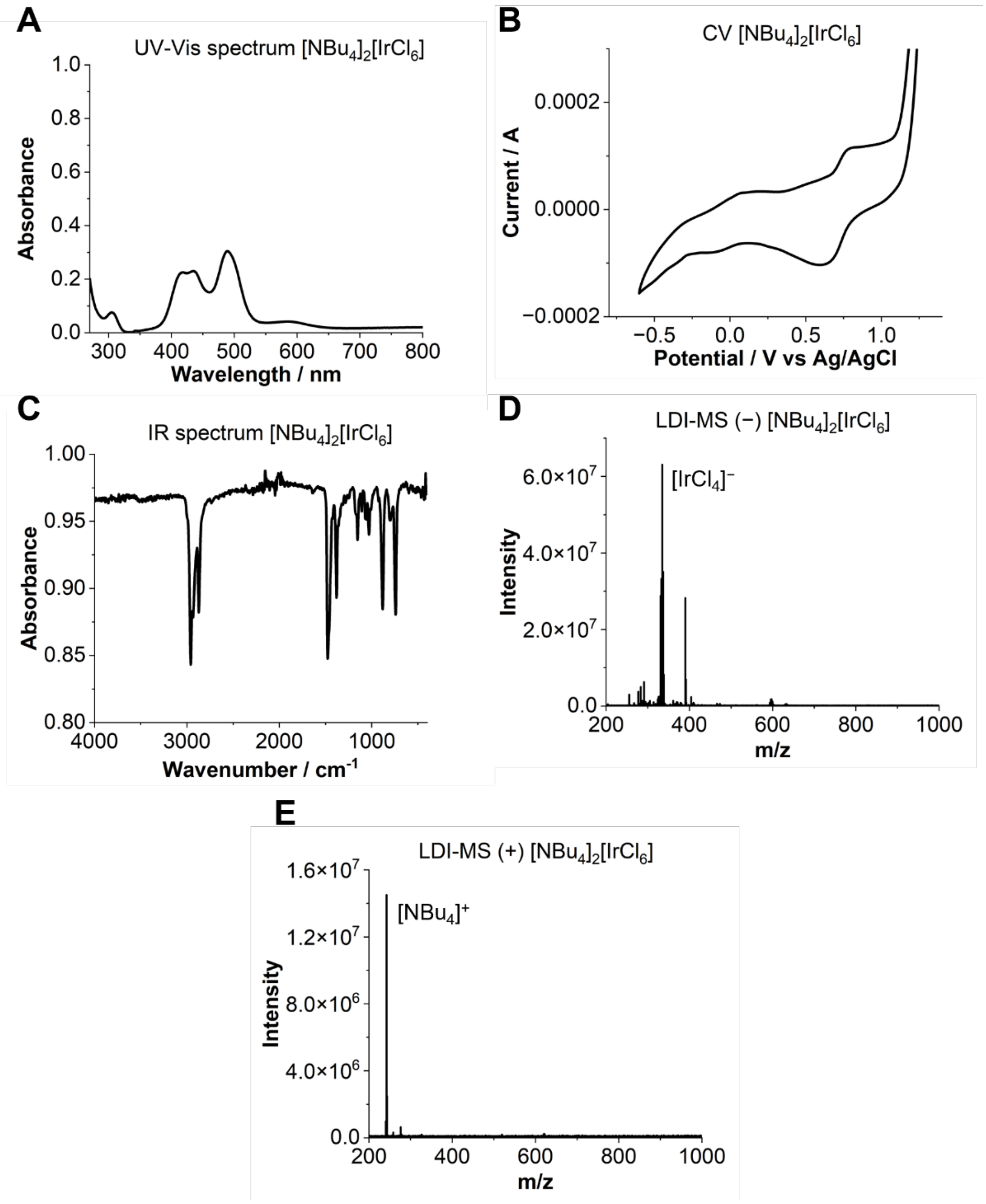

Figure S25. Characterisation data $[NBu_4]_2[IrCl_6]$. **A** UV-Vis spectrum (MeOH) showing characteristic trace of $[IrCl_6]^{2-}$; **B** CV (MeCN, 0.1 M NBu_4PF_6) with $E_{1/2}$ = + 0.72 V vs Ag/AgCl; **C** IR spectrum (neat) $[NBu_4]_2[IrCl_6]$; D LDI-MS (−) $[NBu_4]_2[IrCl_6]$ showing $[IrCl_4]^-$ major species; **E** LDI-MS (+) $[NBu_4]_2[IrCl_6]$ showing $[NBu_4]^+$ major species.

4.5.2. $[NBu_4]_2[Ir_2Cl_{10}]$

$[Ir_2Cl_{10}][NBu_4]_2$ was synthesised by an adaptation of literature procedure.[10] $[NBu_4]_2[IrCl_6]$ (100 mg, 0.15 mmol) was stirred in trifluoroacetic acid (5 mL) under argon at 65 ^{o}C for 18 hours. A fine precipitate was separated by cannula filtration and dried under vacuum, resulting in a purple powder (8 mg, 12 %).

$\boldsymbol{\lambda_{max}}$/ nm (0.5 M HCl) 410, 491

$\mathbf{E_{red}}$ (0.5 M HCl) + 0.45 V vs Ag/AgCl

IR ν_{max}/ cm^{-1} (neat) 3218, 2961, 2873, 1776, 1630, 1468, 1380, 1165, 1058, 983, 883, 736

LDI-MS (−) $[M{-}Cl]^-$ *m/z* 702.59343 (calc. $[Ir_2Cl_9]^-$ *m/z* 702.63907 u), $[M{-}2Cl]^-$ *m/z* 632.65443 (calc. $[Ir_2Cl_7]^-$ *m/z* 632.70200 u) $[M{-}3Cl]^-$ *m/z* 597.68959 (calc. $[Ir_2Cl_6]^-$ *m/z* 597.73353 u)

Note: product soluble in organic solvents such as methanol and acetonitrile but decomposition to $[IrCl_6]^{2-}$ observed rapidly. Colour change from purple to brown observed and UV-Vis spectra taken within five minutes post dissolution show the typical absorbances of $[IrCl_6]^{2-}$. Stability observed in 0.5 M HCl solutions sufficient for characterisation.

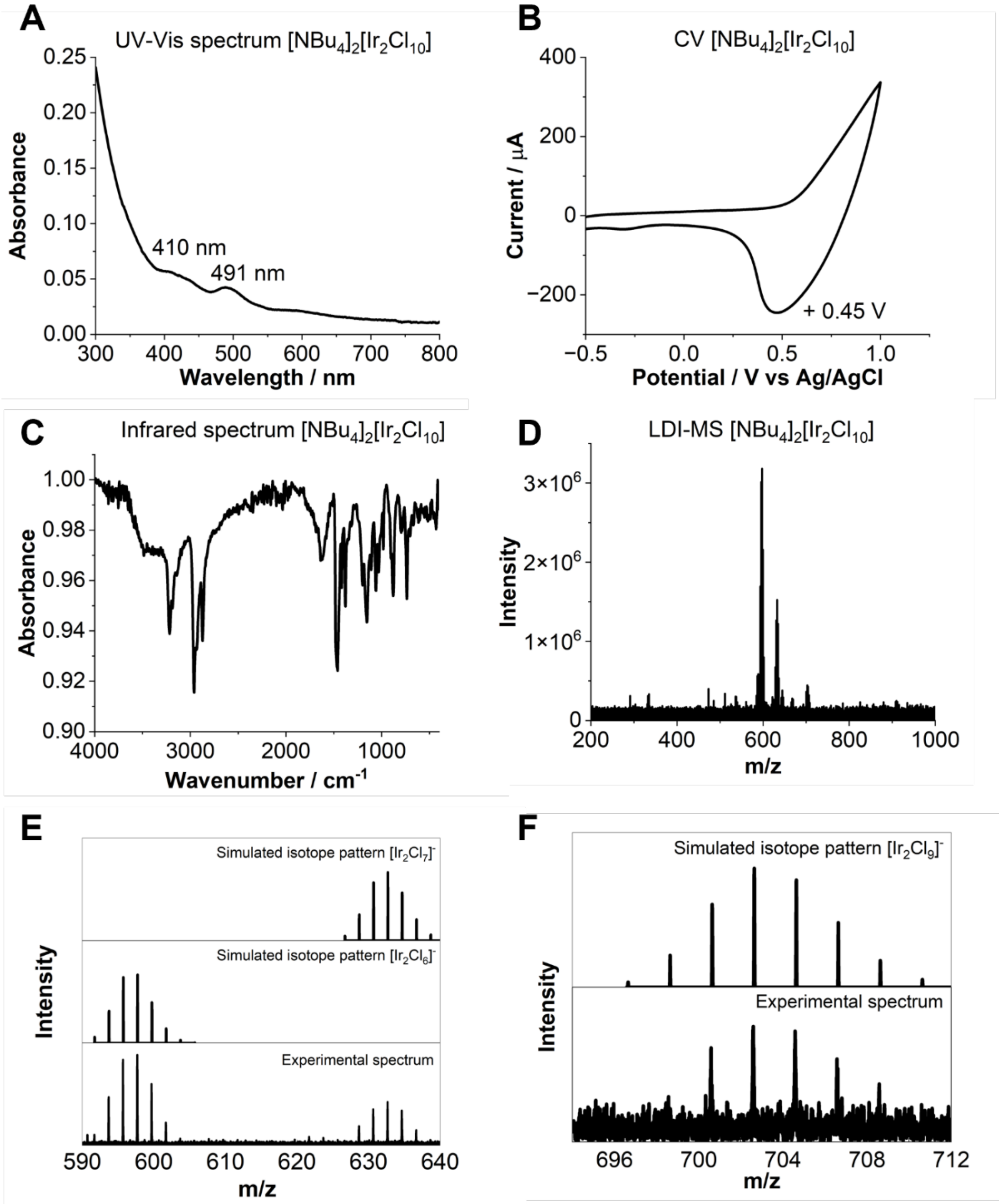

Figure S26. Characterisation of $[NBu_4]_2[Ir_2Cl_{10}]$. **A** UV-Vis spectrum in 0.5 M HCl measured immediately post dissolution in 0.1 cm path length cuvettes; **B** Cyclic voltammogram measured on a screen printed electrode of $[NBu_4]_2[Ir_2Cl_{10}]$ in 0.5 M HCl, showing broad irreversible reduction at + 0.45 V vs Ag/AgCl; **C** IR spectrum (ATR, neat) $[NBu_4]_2[Ir_2Cl_{10}]$ showing absorbances associated with $[NBu_4]^+$ at 2961, 2873, 1468, 1380, 1165, 1058, 883, 736 cm^{-1}; **D** Mass spectrum of $[NBu_4]_2[Ir_2Cl_{10}]$ collected using a matrix-free Laser-Desorption Ionisation (LDI) technique; **E** Simulated isotope patterns of $[Ir_2Cl_6]^-$ and $[Ir_2Cl_7]^-$ matching experimental spectrum; **F** Simulated isotope patterns of $[Ir_2Cl_9]^-$ matching experimental spectrum.

4.6. $K_2[Ir(OH)_n(OD)_{6-n}]$ (n = 0 – 6)

$Na_2[IrCl_6]$ (0.1 g, 0.22 mmol) was dissolved in a solution of KOD in D_2O (2 M, 10 mL) and heated to 130 °C for 15 minutes, resulting in the formation of a purple solution and a black precipitate. The precipitate and solution were separated and the solution was left to sit at room temperature for three hours. Subsequently, this solution was heated to 110 °C until its volume halved, then left to slowly evaporate at room temperature over 36 hours resulting in dark-red prismatic crystals (10 mg, 12 %).

ESI-MS (-) (D_2O): $[[Ir(OD)_6] + Na]^-$ m/z 324.00697 (calc. 324.00736), $[[Ir(OD)_5(OH)] + Na]^-$ m/z 323.00107 (calc. 323.00108), $[[Ir(OD)_4(OH)_2] + Na]^-$ m/z 321.99455 (calc. 321.99480), $[[Ir(OD)_3(OH)_3] + Na]^-$ m/z 320.98844 (calc. 320.98853).

IR (argon) ν_{max}/ cm^{-1} (neat, argon) 3334, 2481, 1668, 1390, 1015, 878, 838, 805, 726, 685, 485

λ_{max} (water, 1 mg mL^{-1}) 314 nm

$E_{1/2}$ (2 M KOH) − 0.45 V vs Ag/AgCl

μ_{eff} (D_2O) 1.97 μ_B

Note: A reduced yield was achieved compared to the synthesis of $K_2[Ir(OH)_6]$. This is attributed to the exclusion of hydrogen peroxide from the synthetic protocol (typically used to reduce the formation of a black precipitate previously described as hydrated iridium dioxide).[1] In this instance, a reduced yield was considered favourable over the introduction of *proteo*-H_2O_2 into the reaction mixture.

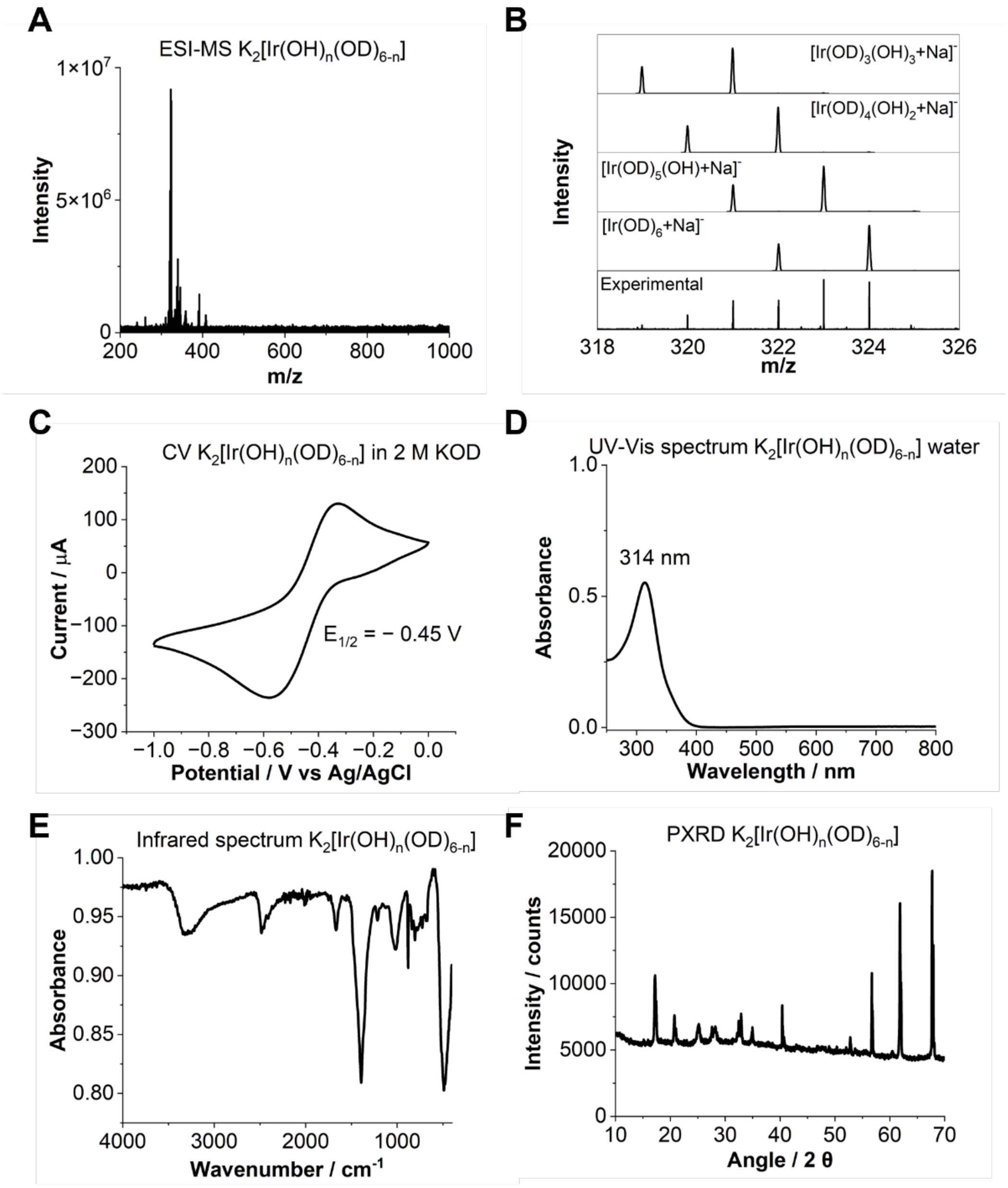

Figure S27. A Mass spectrum (ESI-MS, negative mode) of $K_2[Ir(OH)_n(OD)_{6-n}]$ in D_2O showing major species $[Ir(OD)_6]$ + Na]⁻ *m/z* 323.00107 (calc. 322.99998) and $[[Ir(OD)_5(OH)]$ + Na]⁻ *m/z* 324.00598 (calc. 324.00626 u). **B** Magnification of the mass spectrum showing match between experimental and simulated isotope patterns for $[[Ir(OD)_6]$ + Na]⁻, $[[Ir(OD)_5(OH)]$ + Na]⁻, $[[Ir(OD)_4(OH)_2]$ + Na]⁻ and $[[Ir(OD)_3(OH)_3]$ + Na]⁻. Sodium adducts are frequently observed by ESI-MS thus the presence of the sodium adduct is not taken to be indicative of a sodium counterion in the synthesised complex. **C** Cyclic voltammogram $K_2[Ir(OH)_n(OD)_{6-n}]$ in 2 M KOD showing $E_{1/2} = -0.45$ V vs Ag/AgCl; **D** UV-Vis spectrum $K_2[Ir(OH)_n(OD)_{6-n}]$ in water (0.5 mg mL^{-1}). **E** Infrared spectrum of $K_2[Ir(OH)_n(OD)_{6-n}]$ conducted in an argon-filled glovebox; **F** Powder XRD of crystals $K_2[Ir(OH)_n(OD)_{6-n}]$.

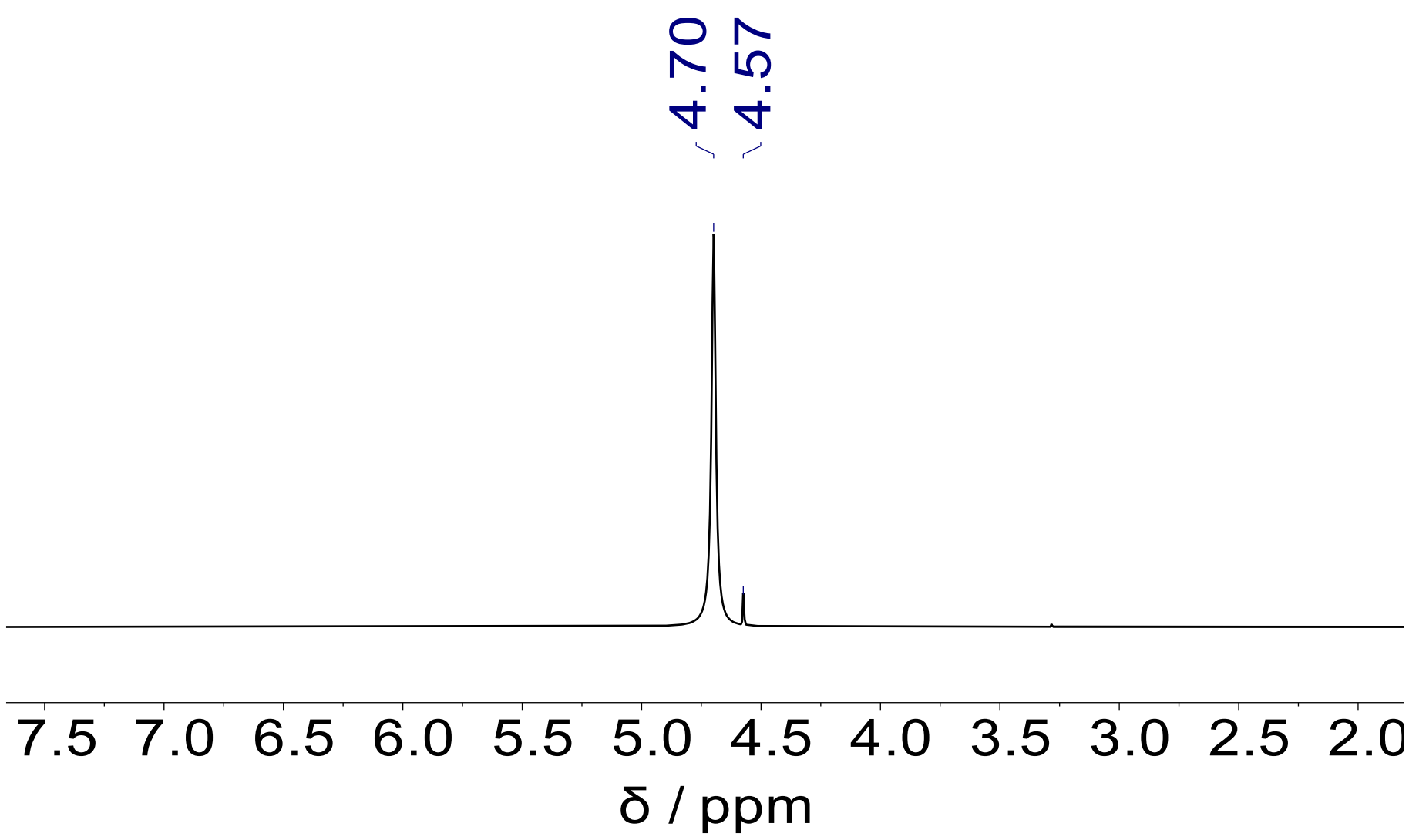

Figure S28. ^1H NMR spectrum (500 MHz, D_2O, 298 K) of $K_2[Ir(OH)_n(OD)_{6-n}]$ in D_2O under argon immediately post dissolution with an internal capillary of D_2O. Paramagnetic shift of solvent residual peak observed, with a Δf of 65 Hz, corresponding to an effective magnetic moment of 1.97 μ_B.

Table S1. Key data for the calculation of the effective magnetic moment for $K_2[Ir(OD)_6]$.

Volume D_2O / mL	0.45
$K_2[Ir(OH)_6]$ / mg	6.4
$K_2[Ir(OH)_6]$ / mmol	0.0172
Spectrometer frequency / Hz	500
Δf / Hz	65
Temperature / K	298
Molar magnetic susceptibility / $m^3\ mol^{-1}$	2.04×10^{-8}
Effective magnetic moment / μ_B	1.969

Note: $K_2[Ir(OH)_n(OD)_{6-n}]$ exhibits increased air sensitivity compared to $K_2[Ir(OH)_6]$. On the timeframe of working up the reaction solution and running IR spectroscopy in air (approximately twenty minutes), decomposition was observed by the presence of peaks associated with iridium oxide, e.g. 1445 cm^{-1}.

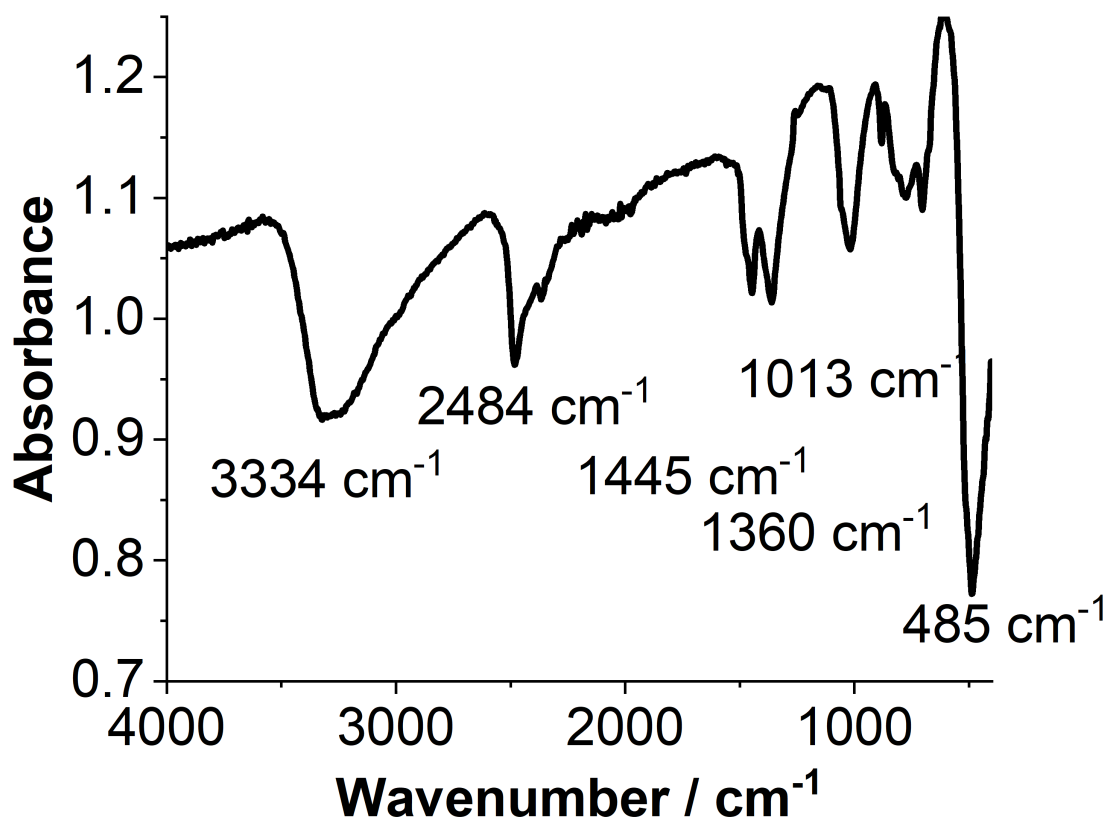

Figure S29. Infrared spectrum $K_2[Ir(OH)_n(OD)_{6-n}]$ measured in air showing absorbance at 1445 cm^{-1} associated with Ir=O multiple bonds.[1, 2]

A reduction in the frequency of the IR bands by close to a factor of the square root of two are observed for the v(O–H) and δ(O–H) as expected upon deuteration. These data are in agreement with the shifts in the IR spectrum resulting from deuteration of $K_2[Pt(OH)_6]$ previously reported.[11]

Table S2. Shifts of key bands in the IR spectrum upon deuteration of $K_2[Ir(OH)_6]$.

$K_2[Ir(OH)_6]$ / cm^{-1}	$K_2[Ir(OH)_n(OD)_{6-n}]$ / cm^{-1}	Assignment
3322	2481	O–H stretch
1011	878	O–H bend
479	485	Ir–O bend

5.0. Optimisation and reaction monitoring of the synthesis of $K_2[Ir(OH)_6]$

5.1. Attempted synthesis of $K_2[Ir(OH)_6]$ following literature reported conditions

5.1.1. Experimental protocol

The literature reported procedure was followed.[1] $Na_2[IrCl_6]$ (0.1 g, 0.22 mmol) was heated to 130 °C in KOH solution (2 M, 10 mL) with H_2O_2 (35 % solution, 0.1 mL) for 50 minutes (*Heating Step 1*). The resulting solution was left to sit at room temperature for three hours (*Three hour sit*). Subsequently, this solution was heated to 130 °C until its volume halved (*Heating Step 2*), then left to slowly evaporate at room temperature over 36 hours resulting in a dark blue solution with a black precipitate. No crystallisation of desired product $K_2[Ir(OH)_6]$ was observed.

5.1.2. Analysis of the crude reaction mixture

The crude reaction mixture post *Heating Step 2* was analysed by cyclic voltammetry and UV-Vis spectroscopy. UV-Vis spectroscopy is unable to distinguish iridium species in basic solutions of Ir–OH containing species, as discussed in the main text. A broad Ligand-to-Metal Charge Transfer (LMCT) band is observed at 313 nm. There is no evidence by CV of the formation of $[Ir(OH)_6]^{2-}$ in solution ($E_{1/2}$ = − 0.44 V).

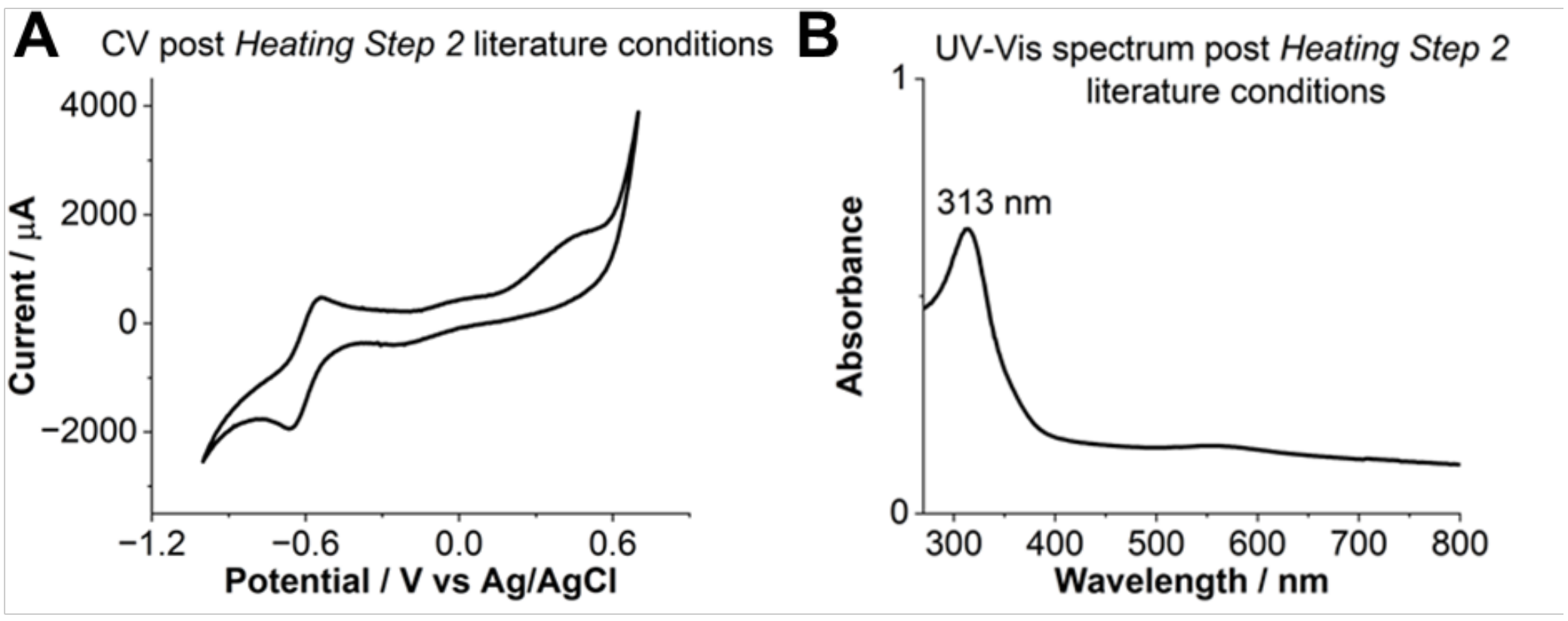

Figure S30. Cyclic voltammogram of crude reaction solution following literature reported conditions for the synthesis of $K_2[Ir(OH)_6]$. **A** SPE-CV post *Heating Step 2* showing absence of redox event associated with $[Ir(OH)_6]^{2-}$ (− 0.44 V vs Ag/AgCl); **B** UV-Vis spectrum of crude reaction solution post *Heating Step 2* showing characteristic broad Ir–O LMCT absorbance at 313 nm.

5.2. Adaptations of protocol

Table S3. Conditions (reaction times, temperatures) tested for the synthesis of $K_2[Ir(OH)_6]$ from $Na_2[IrCl_6]$ with resulting observations and yields of isolated product.

Heating Step 1 / minutes	Heating Step 1 temperature / °C	Observation post Heating Step 1	Heating Step 2 temperature / °C	Yield / %
50	100	Pink solution	120	-
50	130	Blue solution, black precipitate	120	-
15	110	Red-purple	120	-
50	130	Blue solution, black precipitate	110	-
15	130	Red-purple	110	60

Table S4. Adaptations of the described protocol for the synthesis of $K_2[Ir(OH)_6]$ from $Na_2[IrCl_6]$ with resulting observations and yields of isolated product.

Adaptation of procedure	Observation	Isolated product yield / %
Heating step 2 with condenser		24
Workup via basification of crude reaction mixture post *Three hour sit* (instead of conducting *Heating Step 2*)	Cyclic voltammetry shows desired $[Ir(OH)_6]^{2-}$ species forming in solution, but no crystallisation of product occurred.	-
Base – 2 M NaOH	Dark purple solution, black precipitate	-
Base – 2 M LiOH	Purple solution, black precipitate	-
Peroxide added after reduction to Ir(III) occurred (colour change of solution from red-brown to green)	Dark blue/ black solution generated post *Heating Step 1*	-
1 mL 7 % H_2O_2 (scaled to a 0.1 g scale of $Na_2[IrCl_6]$ starting material)	Vigorous bubbling	31
1 mL 1.75 % H_2O_2 (scaled to a 0.1 g scale of $Na_2[IrCl_6]$ starting material)		28

5.3. Supplementary reaction monitoring data during the synthesis of $K_2[Ir(OH)_6]$ from $Na_2[IrCl_6]$

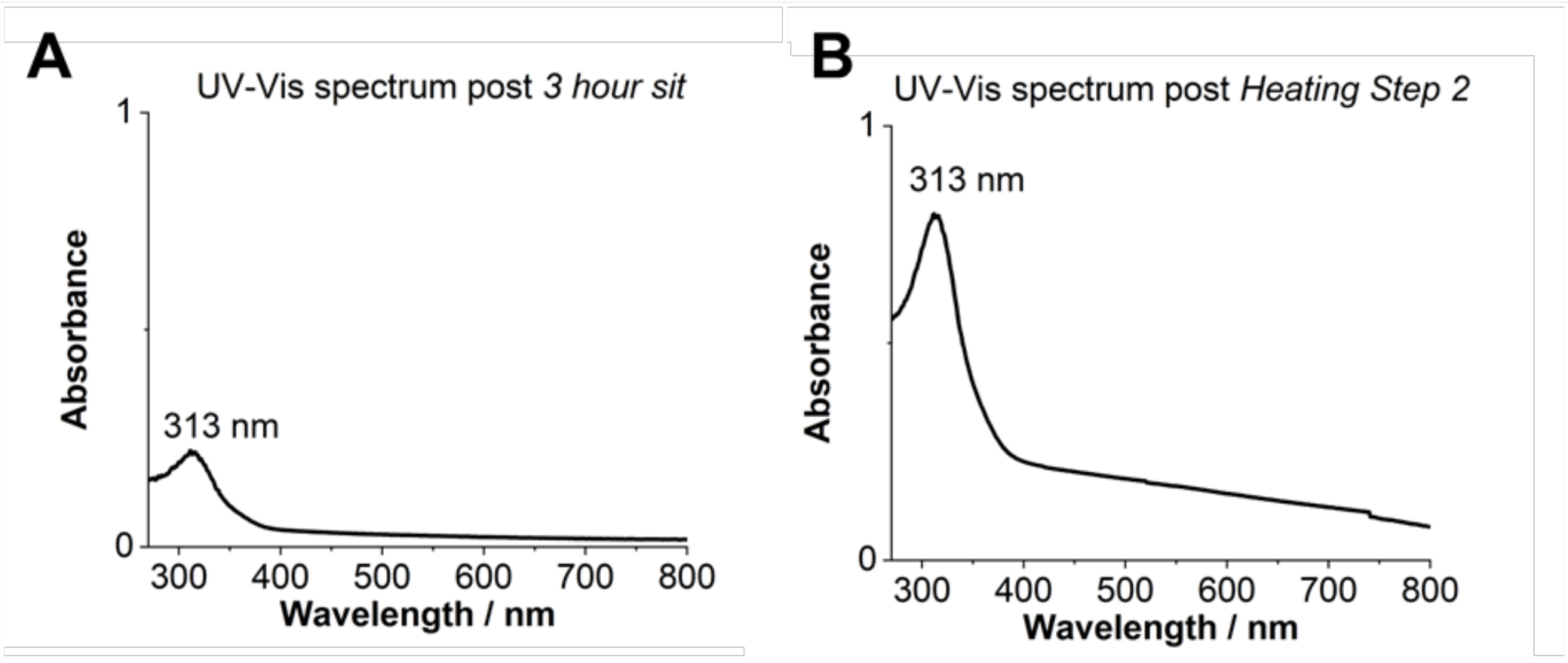

Figure S31. UV-Vis spectra of crude reaction solutions post each key reaction stage, each showing broad absorbance at 313 nm attributed to Ir–O LMCT absorbance. 10 µL aliquots taken of crude solutions and diluted with 290 µL 2 M KOH. Spectra measured in a 0.1 cm path length cuvette in air. **A** UV-Vis spectrum post *three hour sit*; **B** UV-Vis spectrum post *Heating Step 2.* Note the evaporation of approximately half the volume of the reaction solvent during *Heating Step 2* (hence higher concentration sample and stronger absorbance). UV-Vis spectrum of crude reaction solution post *Heating Step 1* shown in main text.

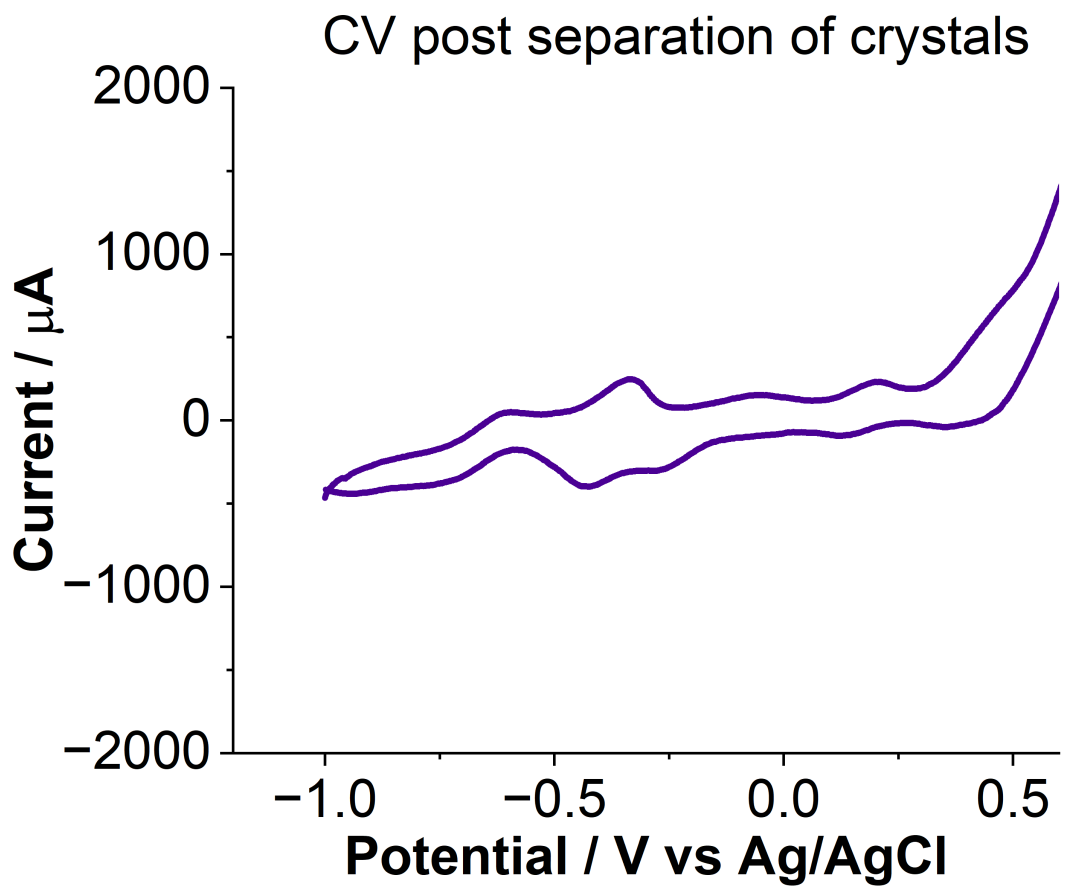

Figure S32. Cyclic voltammogram of crude reaction mixture post separation of crystalline $K_2[Ir(OH)_6]$ after slow evaporation in air over 36 hours from crude reaction mixture post *Heating Step 2*. Mixture of species observed in solution.

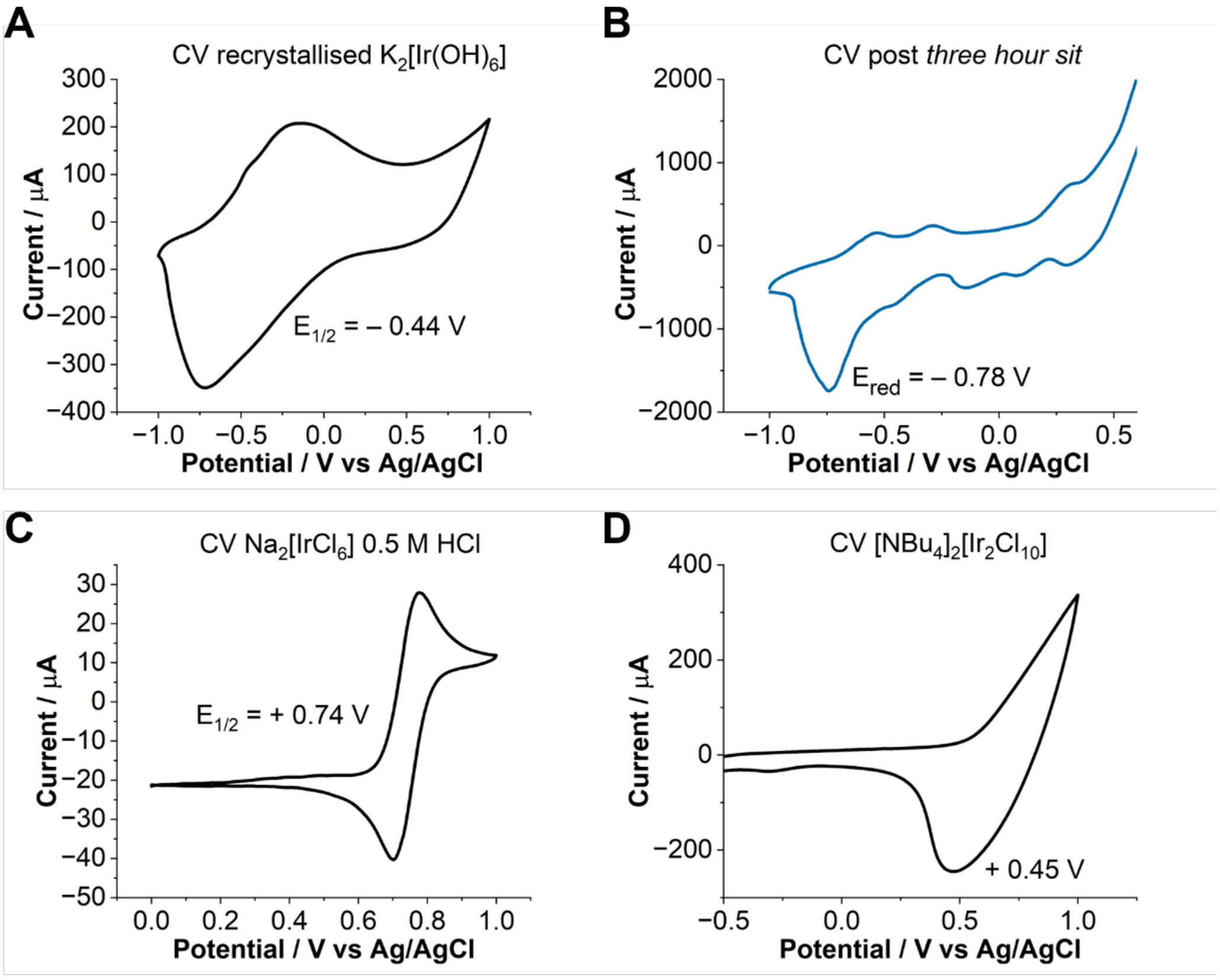

Figure S33. The characteristic electrochemical traces of monomeric and dimeric chloro- and hydroxy-iridate species. **A** CV of $K_2[Ir(OH)_6]$ at – 0.44 V in 2 M KOH; **B** CV of crude reaction mixture post *three hour sit* of $K_2[Ir(OH)_6]$ synthetic protocol showing irreversible reduction at – 0.78 V vs Ag/AgCl (approximately 0.3 V below redox potential of equivalent monomeric species); **C** CV of $Na_2[IrCl_6]$ at + 0.74 V in 0.5 M HCl; D CV of $[NBu_4]_2[Ir_2Cl_{10}]$ in 0.5 M HCl showing broad irreversible reduction at + 0.45 V vs Ag/AgCl (approximately 0.3 V below redox potential of equivalent monomeric species).

5.4. The synthesis of $K_2[Ir(OH)_6]$ from a crude post-refinery chloroiridate solution, $H_2[IrCl_6]$ in HCl

$H_2[IrCl_6]$ in HCl (500 µL, 1.02 mmol) was neutralised by the addition of 2 M KOH (~2 mL). The solution was then made up to 2 M KOH total concentration (25 mL) prior to heating at 130 °C with H_2O_2 (0.25 mL, 35 %) for 15 minutes. The resulting solution was left to sit at room temperature for three hours. Then, this solution was heated at 110 °C until its volume halved and left to slowly evaporate at room temperature over 36 hours, resulting in red crystals of $K_2[Ir(OH)_6]$ (135 mg, 35 %).

6.0. The temporal behaviour of $K_2[Ir(OH)_6]$

6.1. Air-aged crystalline $K_2[Ir(OH)_6]$

After approximately one week of storage of $K_2[Ir(OH)_6]$ in air, a colour change from red to blue was observed, attributed to iridium oxide formation characterised by IR spectroscopy and mass spectrometry.

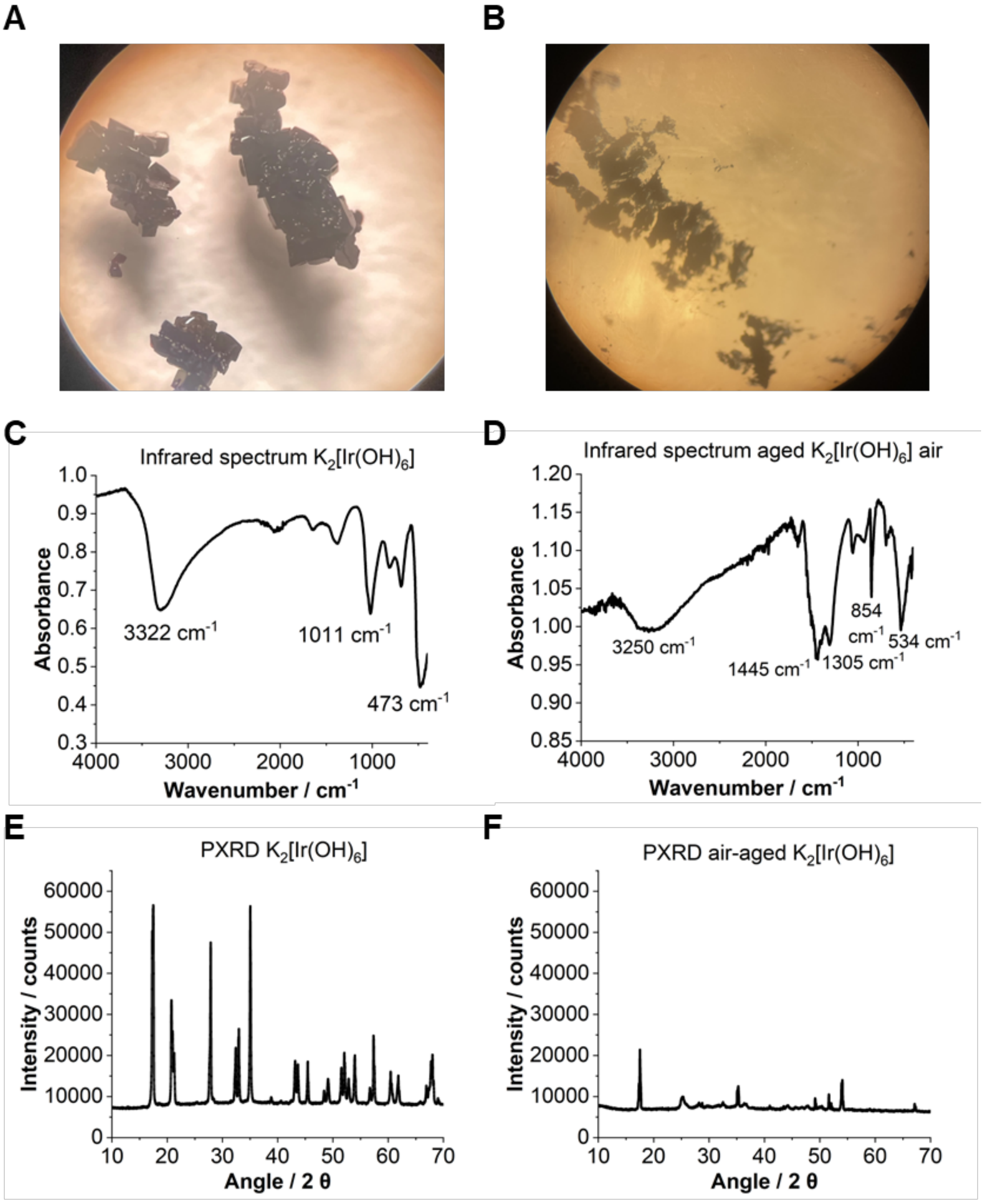

Figure S34. Solid state characterisation of one week air-aged sample of $K_2[Ir(OH)_6]$ showing change in colour from red to blue and loss of crystallinity. **A/B** Photographs taken down a microscope lens showing **A** fresh, red, prismatic crystals of $K_2[Ir(OH)_6]$ and **B** Air-aged sample of $K_2[Ir(OH)_6]$; **C** Infrared spectrum of fresh $K_2[Ir(OH)_6]$ showing characteristic bands at 3300, 1010 and 473 cm^{-1}; **D** Infrared spectrum of air-aged $K_2[Ir(OH)_6]$ showing new bands at 1445, 1305 and 854 cm^{-1}, associated with Ir=O multiple bonds[1, 2]; **E** Powder XRD of fresh $K_2[Ir(OH)_6]$; **F** Powder XRD of air-aged $K_2[Ir(OH)_6]$ showing reduced crystallinity.

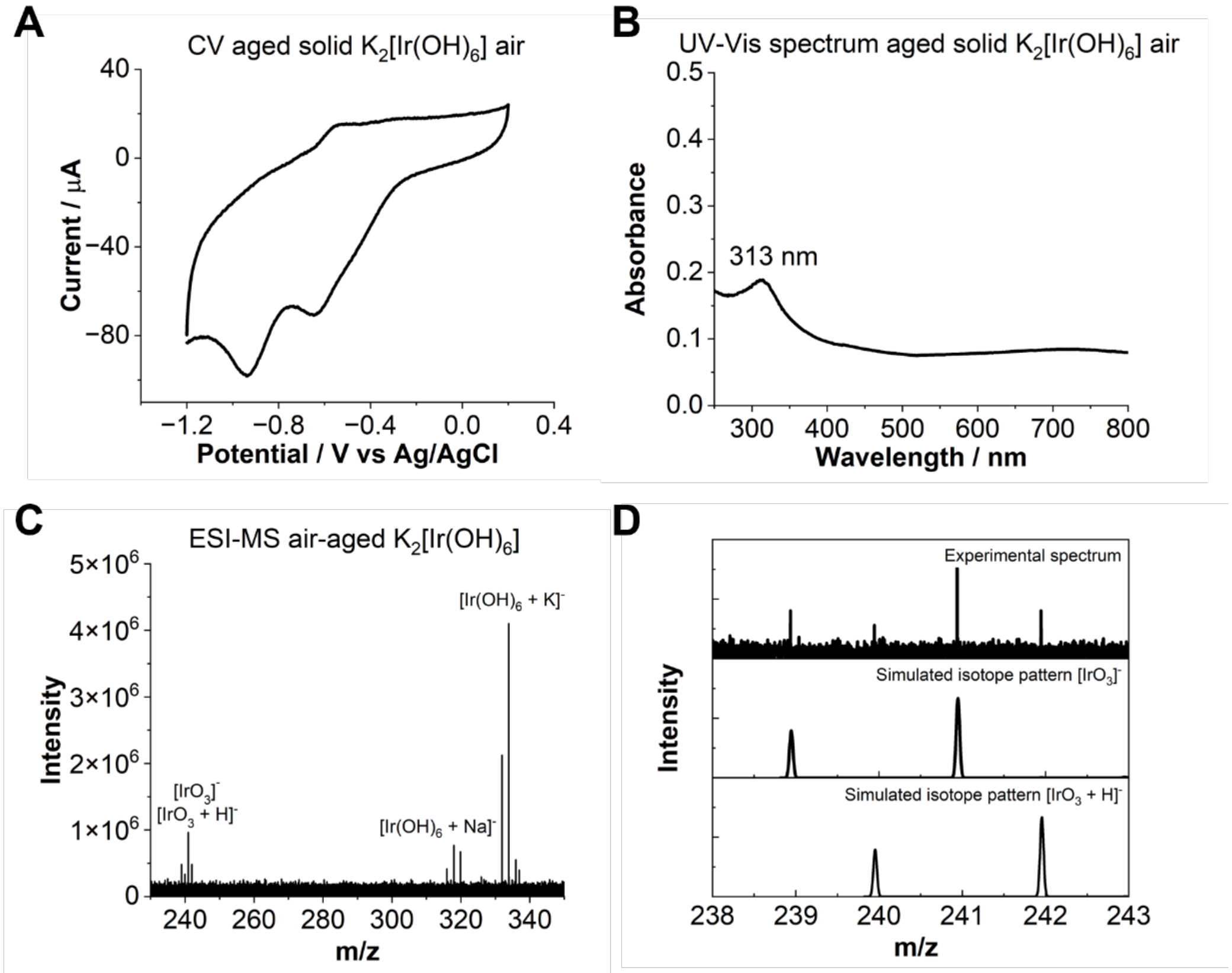

Figure S35. Solution-state characterisation of air-aged $K_2[Ir(OH)_6]$ dissolved in water. **A** CV (2 M KOH) showing two irreversible reduction events at − 0.94 and − 0.64 V vs Ag/AgCl and a minor oxidation event at − 0.54 V. No evidence of the redox event associated with $[Ir(OH)_6]^{2-}$ (− 0.44 V vs Ag/AgCl); **B** UV-Vis spectrum (water, 1 mg mL^{-1}, 0.1 cm path length cuvette) showing typical 313 nm LMCT band of Ir–OH containing species; **C** Mass spectrum (ESI−) of air-aged sample of $K_2[Ir(OH)_6]$ dissolved in water (0.1 mg mL^{-1}) showing events assigned as $[[Ir(OH)_6] + K]^-$ 333.92288 (calc. 333.94346), $[[Ir(OH)_6] + Na]^-$ 317.95085 (calc. 317.96970), $[IrO_3]^-$ 240.93818 (calc. 240.94823), $[[IrO_3] + H]^-$ 241.94597 (calc. 241.95605) matching simulated isotope patterns; **D** Observed and simulated isotope patterns of $[IrO_3]^-$ and $[[IrO_3]+H]^-$.

6.2. Air-aged aqueous solutions of $K_2[Ir(OH)_6]$

6.2.1. $K_2[Ir(OH)_6]$ speciation in 2 M KOH over 96 hours in air

6.2.1.1. UV-Vis spectrum

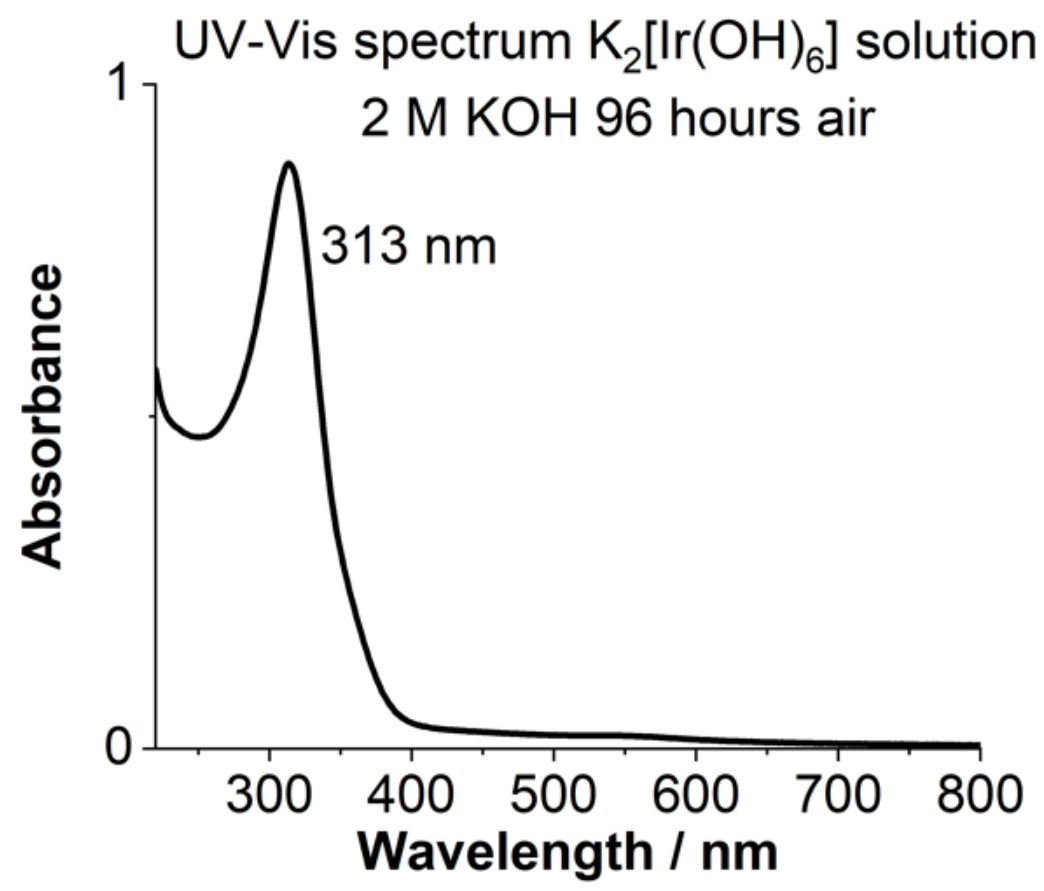

Figure S36. UV-Vis spectrum of $K_2[Ir(OH)_6]$ in 2 M KOH at 10 mg mL^{-1} concentration immediately after 96 hours equilibration time in air. Solution remains purple, and 580 nm band associated with iridium oxide nanoparticles remains absent.

6.2.2. $K_2[Ir(OH)_6]$ speciation in non-buffered aqueous solution over 96 hours in air

6.2.2.1 UV-Vis spectrum

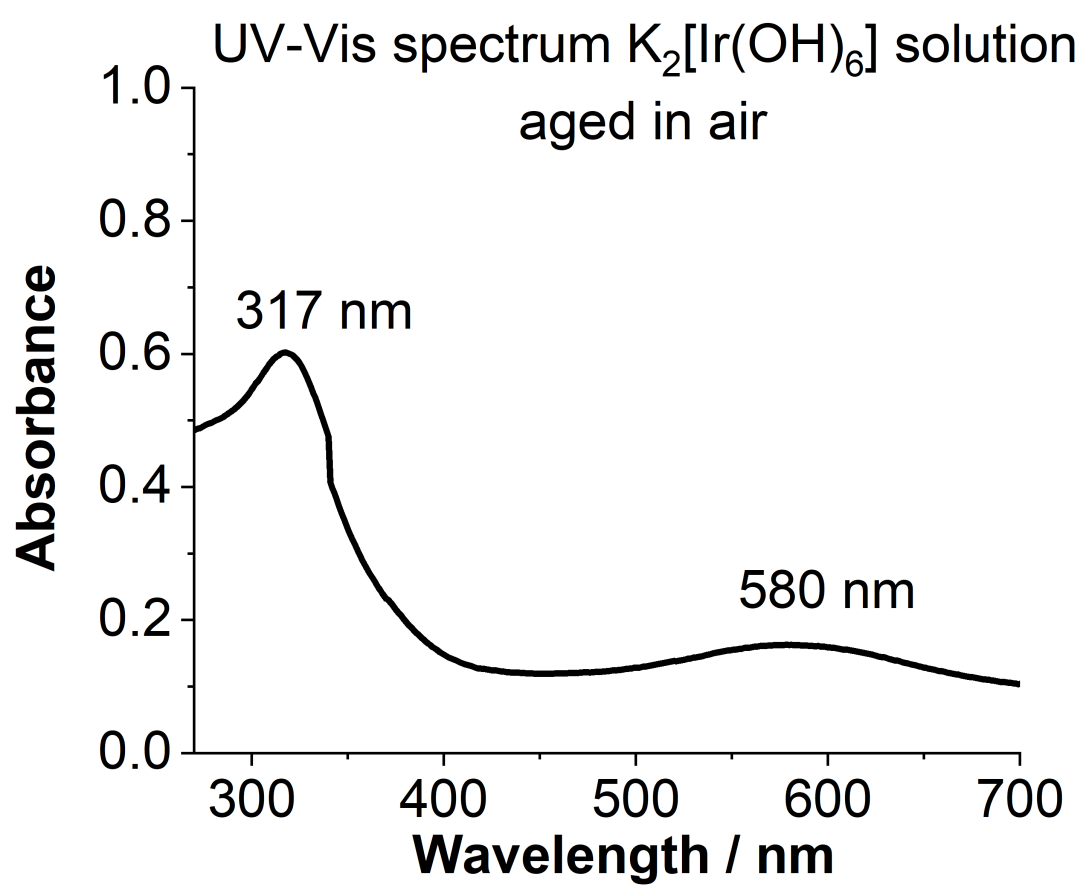

Figure S37. UV-Vis spectrum 96 hour air-aged aqueous solution $K_2[Ir(OH)_6]$ showing broad 580 nm absorbance associated with iridium oxide nanoparticles.

6.2.2.2. Electron Microscopy data

Experimental

High-resolution transmission electron microscopy (HRTEM) was performed using a double aberration-corrected JEOL NeoARM microscope operated at an accelerating voltage of 200 kV. Conventional low-magnification TEM imaging and selected area electron diffraction (SAED) were conducted on a JEOL 2100+. Scanning electron microscopy (SEM) and energy-dispersive X-ray (EDX) spectroscopy were carried out using a JEOL JSM-7800F field-emission SEM.

Solutions of $K_2[Ir(OH)_6]$ in deionised water were prepared at a concentration of 10 mg mL^{-1}. These were left sealed in air for 96 hours, during which time a colour change from purple to deep blue was observed. The resulting blue solutions were analysed by UV-Vis spectroscopy (main text) and Transmission Electron Microscopy (TEM).

Supporting data

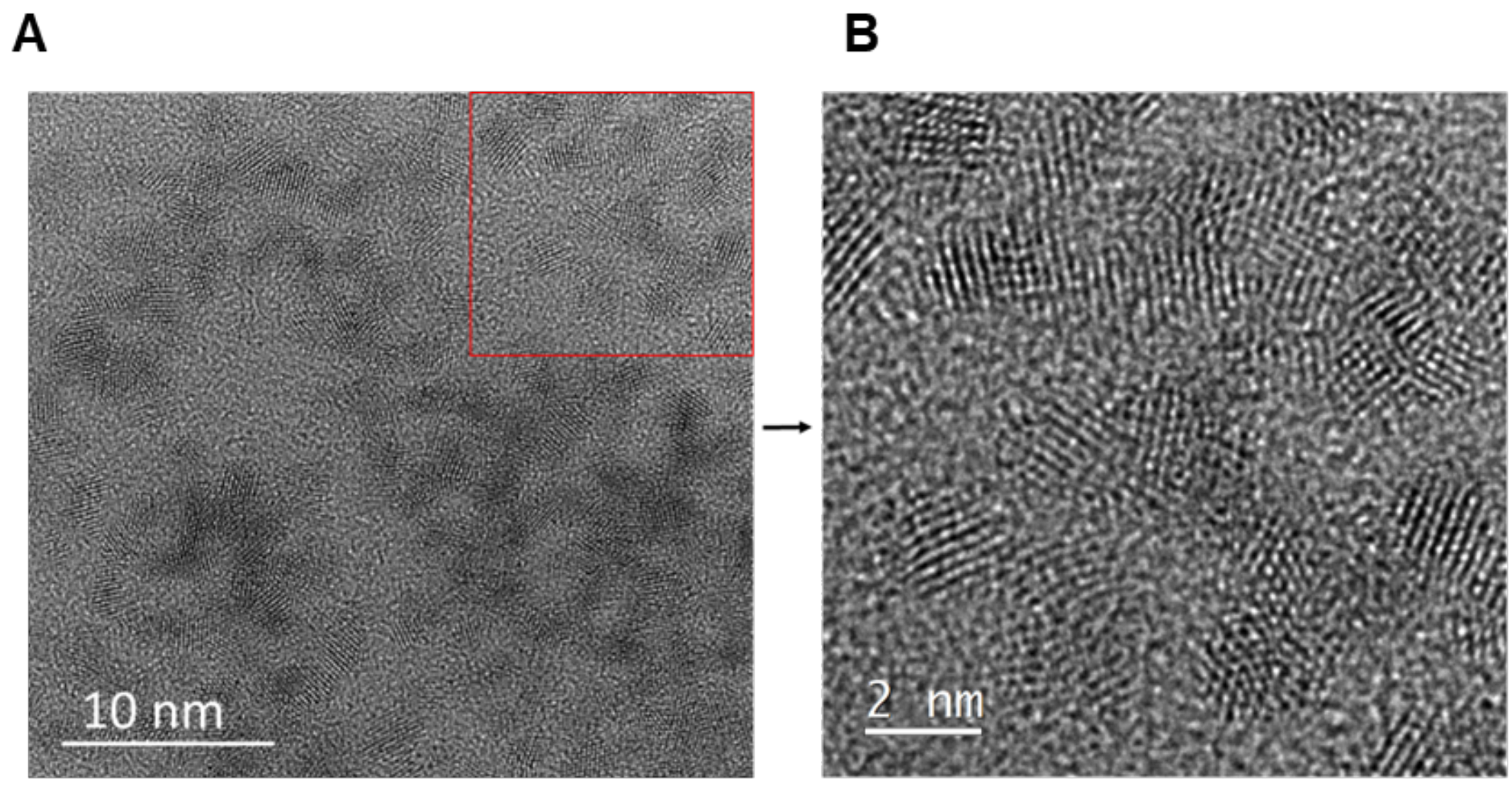

Figure S38. A High resolution TEM image of dispersed IrO_2 nanoparticles on a carbon support. **B** Magnified selected area from panel A (in red) showing different orientations of the IrO_2 with their atomic planes clearly resolved.

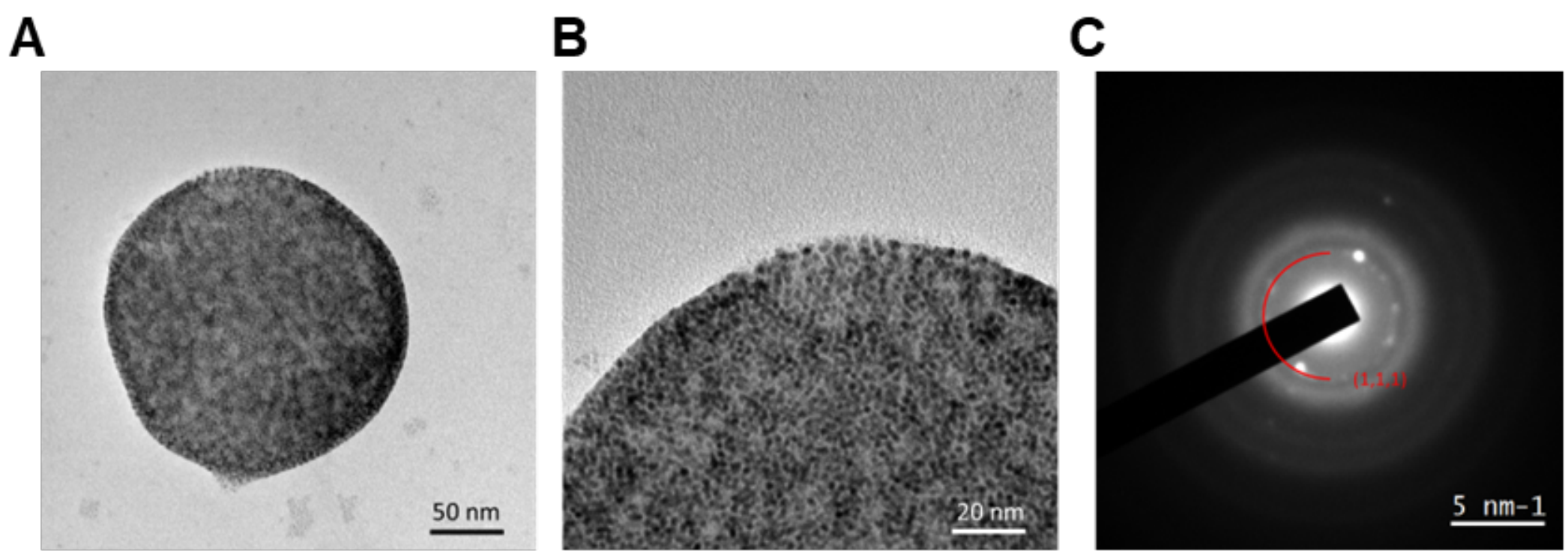

Figure S39. A Low magnification TEM image of IrO_2 nanoparticle cluster. **B** High magnification TEM image from the top of the cluster clearly showing individual IrO_2 NPs within the cluster. **C** Selected area diffraction from the cluster of IrO_2 nanoparticles presented in panel A, showing several diffraction rings with the most intense (111) diffraction rings that corresponds to (111) atomic planes of the IrO_2 cubic phase.

6.3. Argon-aged aqueous solutions of $K_2[Ir(OH)_6]$

6.3.1. CV argon-aged aqueous solution $K_2[Ir(OH)_6]$

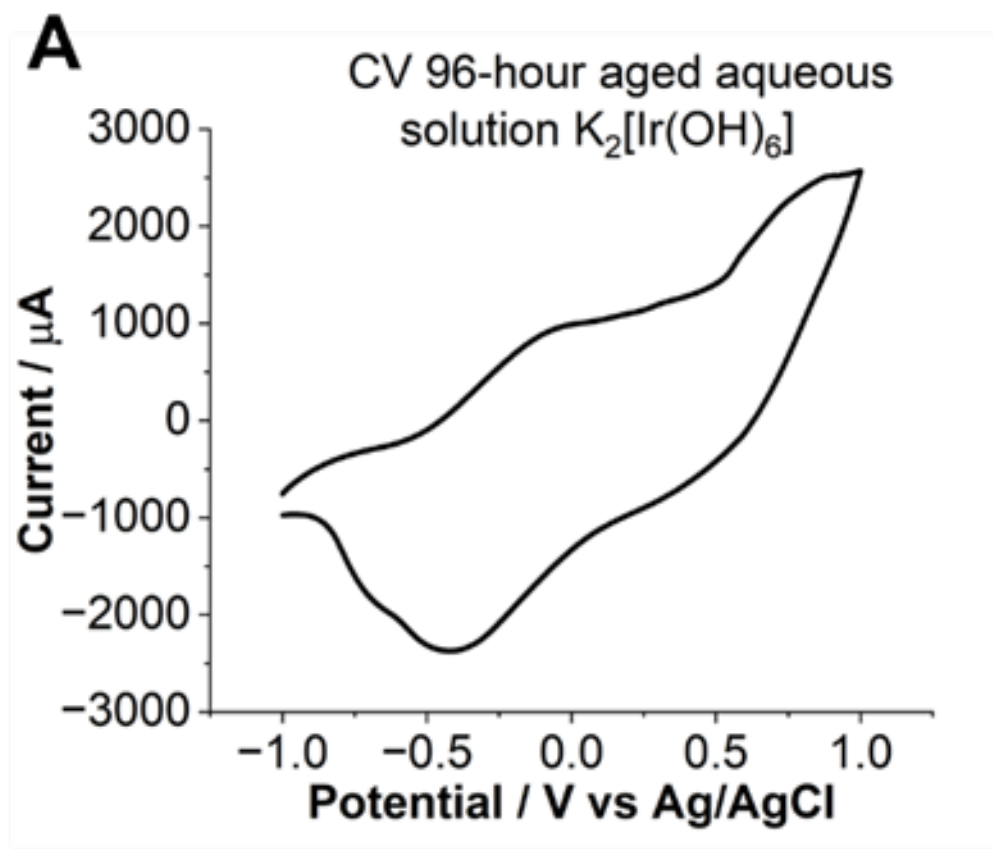

Figure S40. SPE-CV data of a 96-hour argon-aged aqueous solution of $K_2[Ir(OH)_6]$, 10 mg mL^{-1}. $E_{1/2}$ = − 0.30 V vs Ag/AgCl.

6.3.1.1. The impact of electrolyte concentration on cyclic voltammetry using screen printed electrodes

Given the significantly reduced interelectrode distance compared to typical three electrode cell set-ups, screen printed electrodes enable solutions with lower concentrations of electrolyte to be run. Whilst data collected in this way may be unsuitable for quantitative analysis, it avoids the possibility of the presence of electrolyte impacting speciation. Thus, cyclic voltammetry of iridium solutions without the addition of supporting electrolyte was considered preferable in this instance. CV data are presented here for the purpose of qualitative comparison.

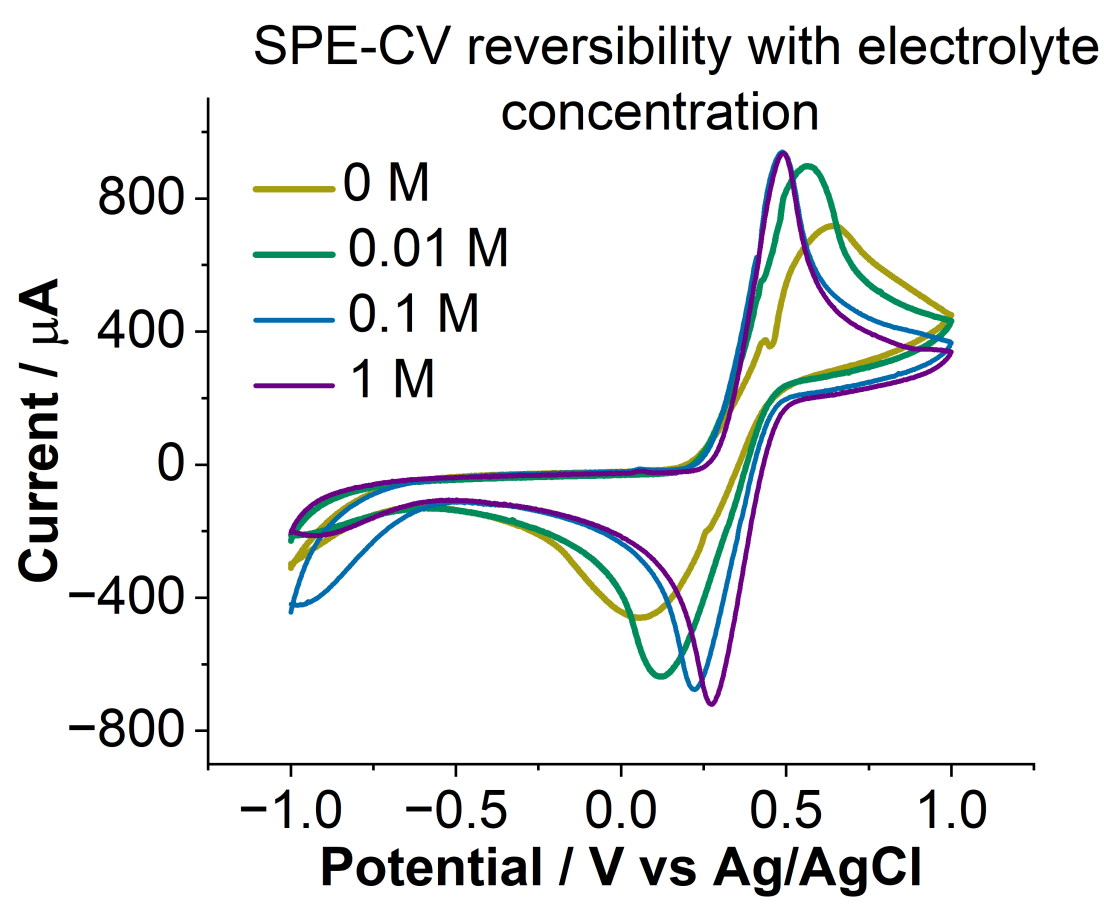

Figure S41. The effect of electrolyte concentration on the reversibility of the redox event of ferrocenylmethyl-trimethylammonium chloride. Reversibility characterised by the peak-to-peak separation, for which an optimal value for a reversible event is 0.059 V.

Solutions of (ferrocenylmethyl)-trimethylammonium chloride (10 mg mL^{-1}) were prepared with various concentrations of supporting electrolyte (between 0 – 1 M KCl). Cyclic

voltammetry was run with a potential window of −1.0 – 1.0 V vs Ag/AgCl using screen-printed electrodes at a scan rate of 0.3 V s^{-1}.

Table S5. Data showing the impact of electrolyte concentration on cyclic voltammetry conducted on screen-printed electrodes, by impact on the peak-to-peak separation of (ferrocenylmethyl)-trimethylammonium chloride.

Electrolyte concentration / M	Anodic potential / V vs Ag/AgCl	Cathodic potential / V vs Ag/AgCl	$E_{1/2}$ / V vs Ag/AgCl	Peak-to-peak separation / V
0	0.646	0.056	0.351	0.590
0.01	0.56	0.118	0.339	0.442
0.1	0.488	0.224	0.356	0.264
1	0.49	0.274	0.382	0.216

Deviations from 0.059 V optimum peak-to-peak separation of this magnitude are common. $E_{1/2}$ can be determined even in solutions containing no electrolyte, with the caveat that peak-to-peak separation observed is approximately double that observed compared to solutions with the optimum electrolyte concentration of 0.1 – 1 M. For qualitative purposes, therefore, SPE-CVs can be used to identify species in solutions with no electrolyte.

6.3.2. X-ray absorption spectroscopy (XAS) data showing the speciation of $K_2[Ir(OH)_6]$ in water under argon over time

6.3.2.1. Experimental protocol

X-ray absorption fine structure (XAFS) spectroscopy measurements were performed in transmission mode at the Durham University XAFS facility using a EasyXAFS300+ using the (8,4,0) reflection of a Si (2,1,0) spherically bent crystal analyser (SBCA), a 1200 W proto-X-ray tube with an Ag anode. The power of the X-ray source was set to maintain a collection deadtime on the silicon drift detector of <30 %.

Powder samples were prepared for Mn K-edge XAFS measurements by adding 15 - 30 mg of each catalyst and 100-110 mg of polyethylene glycol binder and grinding in an agate mortar and pestle until homogenous (masses targeting optimal transmission edge jumps versus total absorption, or determined by material available for recycle experiments). Pellets were produced from the resulting mixture by transfer to a 1.0 cm diameter die and applying up to 3 tonnes of pressure for 180 s using a Specac hydraulic press. For $K_2[Ir(OH)_6]$, the steps before pressing were conducted in a nitrogen filled glove bag and the pellet was quickly sealed in Kapton after pressing to minimise degradation. The samples were mounted on 7 mm inner diameter steel washers. The aqueous $K_2[Ir(OH)_6]$ samples were transferred between a sample vial and a thin walled 2.5 mm OD quartz capillary in the bottom of a Schlenk tube that was open, but under a high flow of nitrogen, and the capillary was then sealed with plasticine and mounted behind a 1 mm slit in a steel plate on the sample stage.

Spectra were obtained around the step edge from 11200 to 11255 eV with 0.5 eV steps and 3.0 s dwell time, and at lower resolution above and below the edge for normalisation. A

minimum of four spectra were acquired for each sample, checked for consistency, summed and normalised. Energy calibration was carried out by scanning a metallic Pt foil (Goodfellow Advanced Materials) using the same acquisition parameters and correcting to the maximum of the first derivative at 11564 eV for the Pt L3 transition. Transmission spectra were obtained using a separately recorded background (I_0) scan (with the same slit/holder and a water filled capillary or a blank washer of the size as used for the sample). Data collected from the instrument was background and energy corrected using the python based EasyXAFS software (easyXAFS, WA). Processing of XAFS data produced was performed using IFEFFIT with the Demeter package using Athena for normalisation, plotting and edge position determination.[12]

6.3.2.2. Supporting data

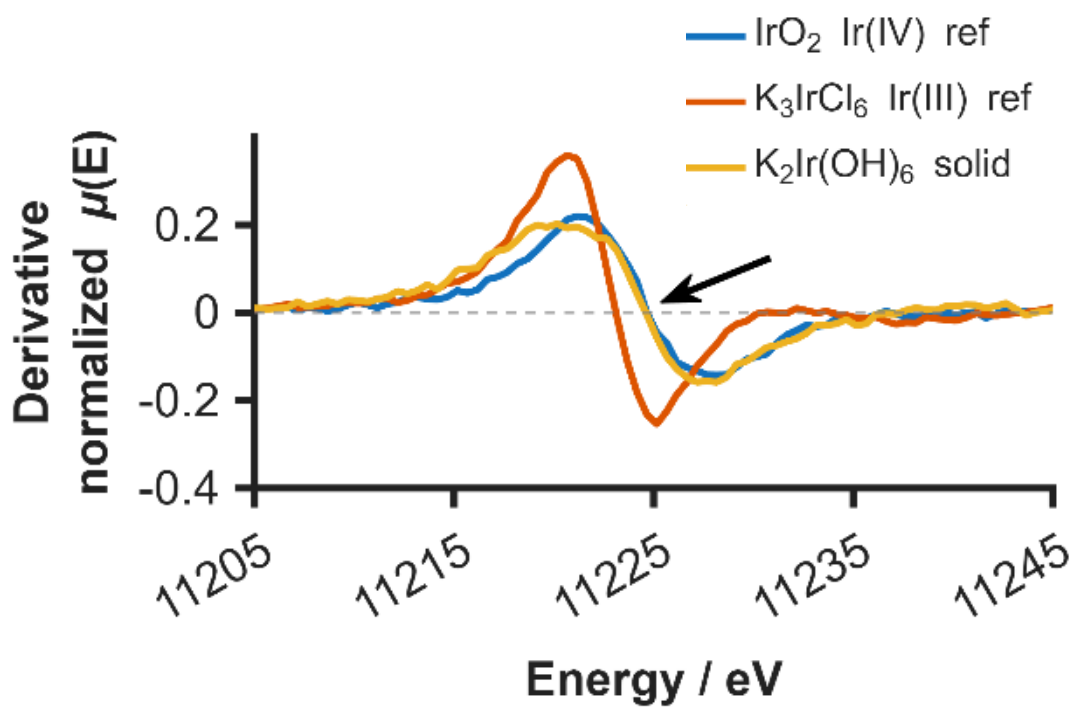

Figure S42. Derivative spectra of Ir L_3-edge X-ray Absorption Spectrum of solid $K_2[Ir(OH)_6]$ in the XANES (X-ray Absorption Near Edge Structure) region shown in comparison to $K_3[IrCl_6]$ and IrO_2 reference compounds for Ir(III) and Ir(IV) respectively. The zero crossing point denoted by the arrow shows the shift in the white line positions.

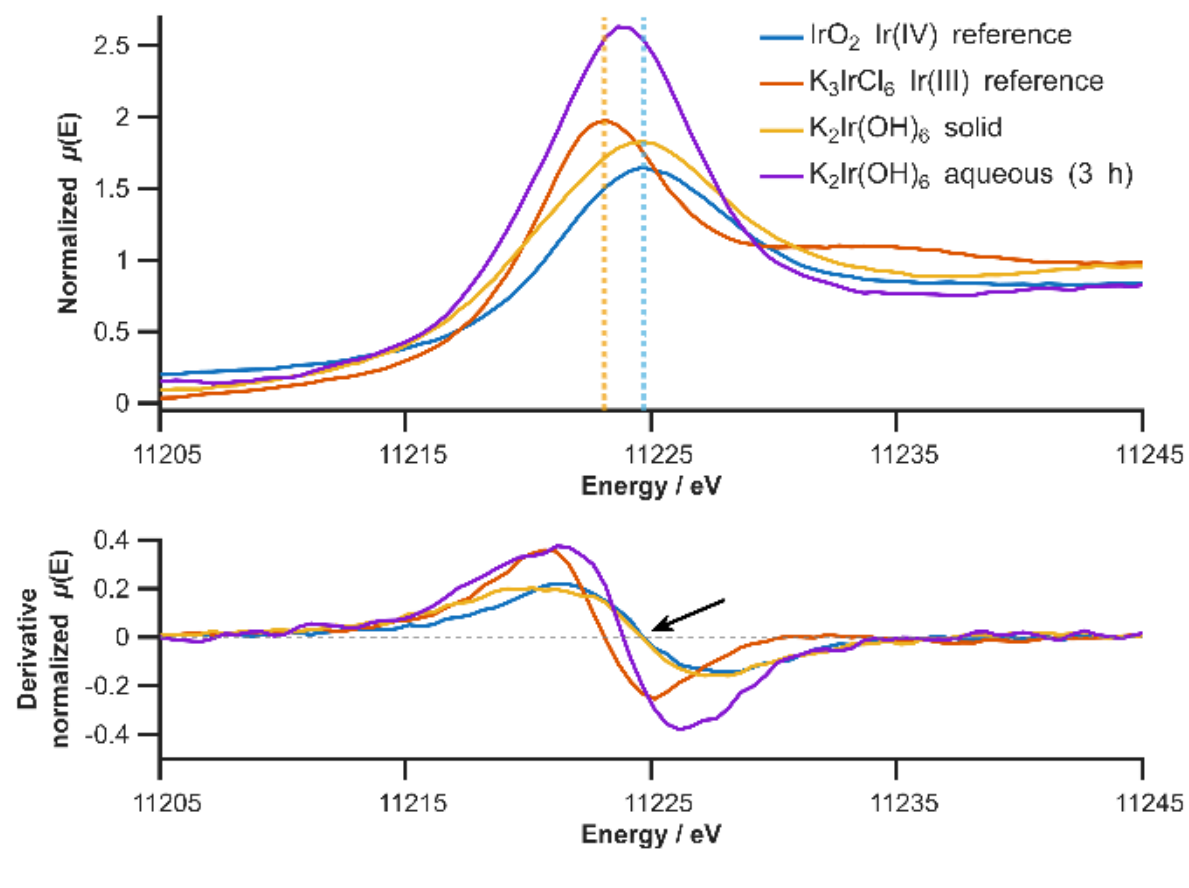

Figure S43. A version of main text Figure 3G, showing the Ir L_3-edge X-ray Absorption Spectrum of solid $K_2[Ir(OH)_6]$ in the XANES (X-ray Absorption Near Edge Structure) region now also including $K_2[Ir(OH)_6]$ after dissolving in water (after ~ 3 hours), still shown in comparison to $K_3[IrCl_6]$ and IrO_2 reference compounds for Ir(III) and Ir(IV), respectively, along with derivative spectra, where the shift in white line positions are easier to visualise by the zero-crossing point (denoted by the arrow). The vertical dashed lines denote are a guide to the eye for the white line peak maxima of the two reference compounds Ir(III) orange and Ir(IV) blue. This clearly shows a shift in the white line position, intensity and overall peak width (associated with reduced e_g vs $t2_g$ filling or spacing, which indicates a decrease in oxidation state at the metal towards Ir(III) in the aqueous form versus the solid).[13]

6.3.3. The Evans method for monitoring the speciation of $K_2[Ir(OH)_6]$ in water under argon over time

Magnetic susceptibility measurements were conducted by the Evans method.[14]

$K_2[Ir(OH)_6]$ (13 mg, 0.0349 mmol) was dissolved in D_2O (0.5 mL) under argon and transferred by cannula to a Youngs NMR tube containing a flame sealed glass capillary filled with D_2O. A proton NMR spectrum was acquired immediately post dissolution of the $K_2[Ir(OH)_6]$ (data collected within 20 minutes of dissolution) and after three hours of storage under argon. No change in Δf (Hz) between the solvent peak and the paramagnetic shifted solvent peak was observed between the two samples.

Table S6. Key data for the calculation of the effective magnetic moment of $K_2[Ir(OH)_6]$ in solution by the Evans method.

Volume D_2O / mL	0.5
$K_2[Ir(OH)_6]$ / mg	13
$K_2[Ir(OH)_6]$ / mmol	0.0349
Spectrometer frequency / Hz	600
Δf / Hz	138
Temperature / K	298
Molar magnetic susceptibility / $m^3\ mol^{-1}$	1.98×10^{-8}
Effective magnetic moment / μ_B	1.937

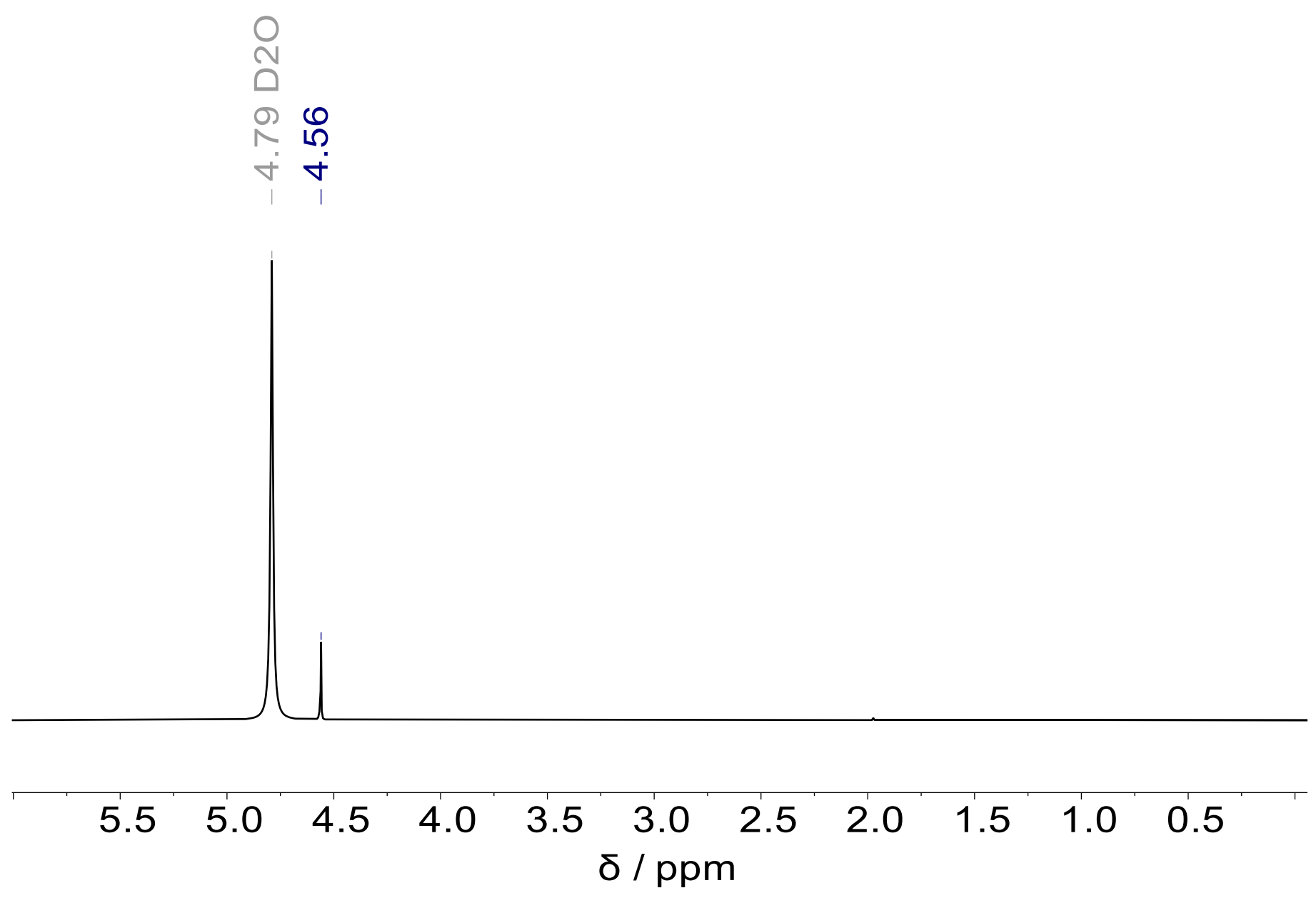

Figure S44. ^1H NMR spectrum (600 MHz, D_2O, 298 K) of $K_2[Ir(OH)_6]$ in D_2O under argon immediately post dissolution with an internal capillary of D_2O. Paramagnetic shift of solvent residual peak observed, with a Δf of 138 Hz, corresponding to an effective magnetic moment of 1.94 μ_B.

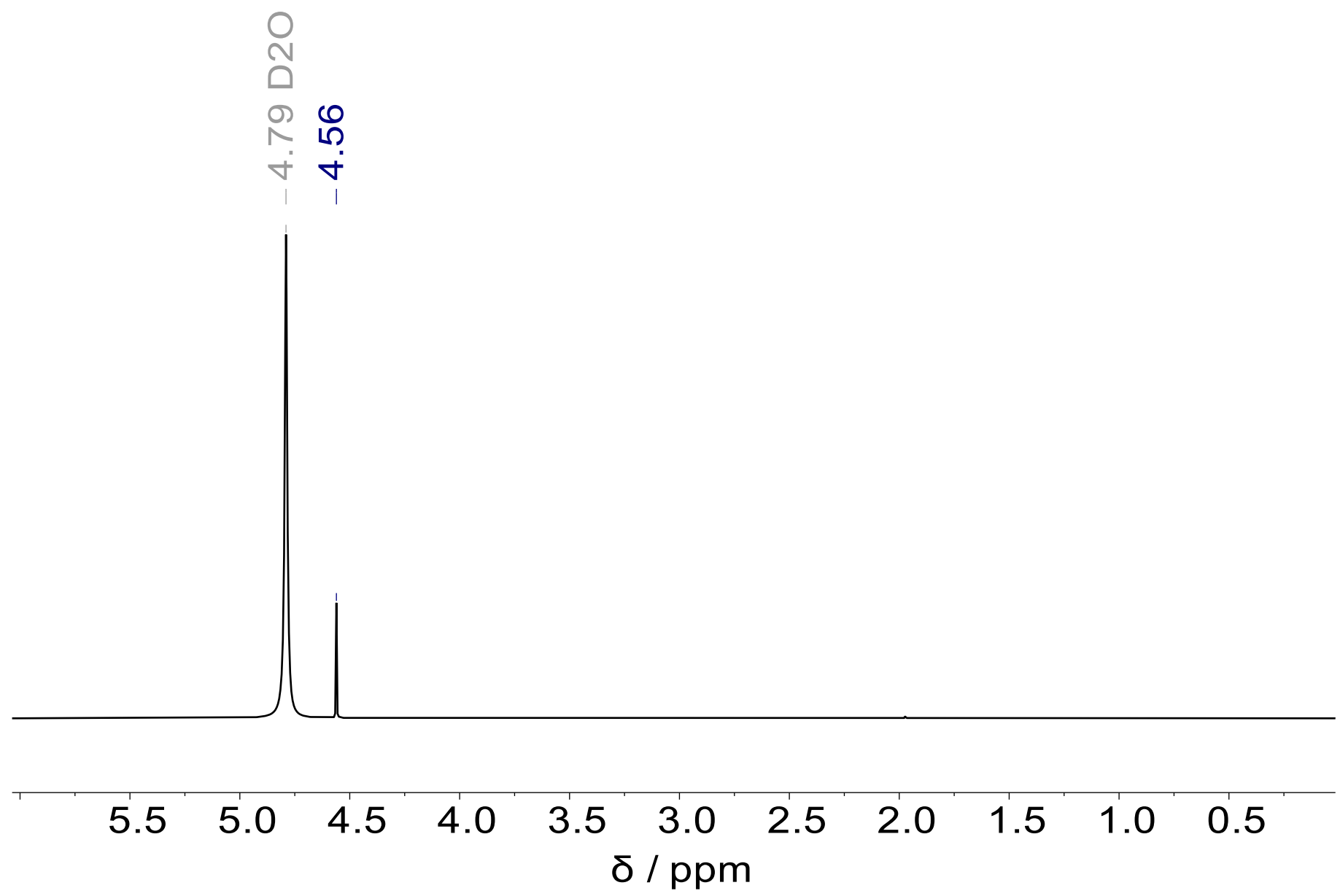

Figure S45. ^{1}H NMR spectrum (600 MHz, *D_2O*, 298 K) of $K_2[Ir(OH)_6]$ in *D_2O* after 96 hours storage under argon with an internal capillary of *D_2O*. Paramagnetic shift of solvent residual peak observed, with a Δf of 138 Hz, corresponding to an effective magnetic moment of 1.94 μ_B.

7.0. Optimisation of the synthesis of $Ir(acac)_3$ from $K_2[Ir(OH)_6]$

7.1. Tabulated conditions

Table S7. Optimisation experiments for the synthesis of $Ir(acac)_3$ from $K_2[Ir(OH)_6]$.

Conditions	Equiv. acac	Solvent	Temperature / °C	Reaction time / days	Isolated yield $Ir(acac)_3$ / %
A	excess (3.5 mL)	neat acac	25	7	16
B	excess (3.5 mL)	neat acac	50	7	27
C	excess (0.5 mL)	water: acac	50	7	67
D	excess (0.5 mL)	water: acac	25	20	73
E	4	water	25	10	41
F	4	water	50	10	73

7.1.1. Alternative workup for the synthesis of $Ir(acac)_3$ from $K_2[Ir(OH)_6]$ using water:acac, exemplified by conditions C

Where excess acetylacetone was used, the product $Ir(acac)_3$ was soluble in the resulting water:acac solvent system. Thus, an adapted work-up procedure was employed:

$K_2[Ir(OH)_6]$ (50 mg, 0.13 mmol) was dissolved in water (3.5 mL) with acetylacetone (0.5 mL, 4.9 mmol) under argon and heated to 50 °C for one week. The solvent was removed *in vacuo* and the crude product washed with cold dry diethyl ether under argon. The resulting yellow powder was dried under vacuum yielding $Ir(acac)_3$ as a yellow powder (44 mg, 67 %).

7.2. NMR spectra of crude reaction solutions

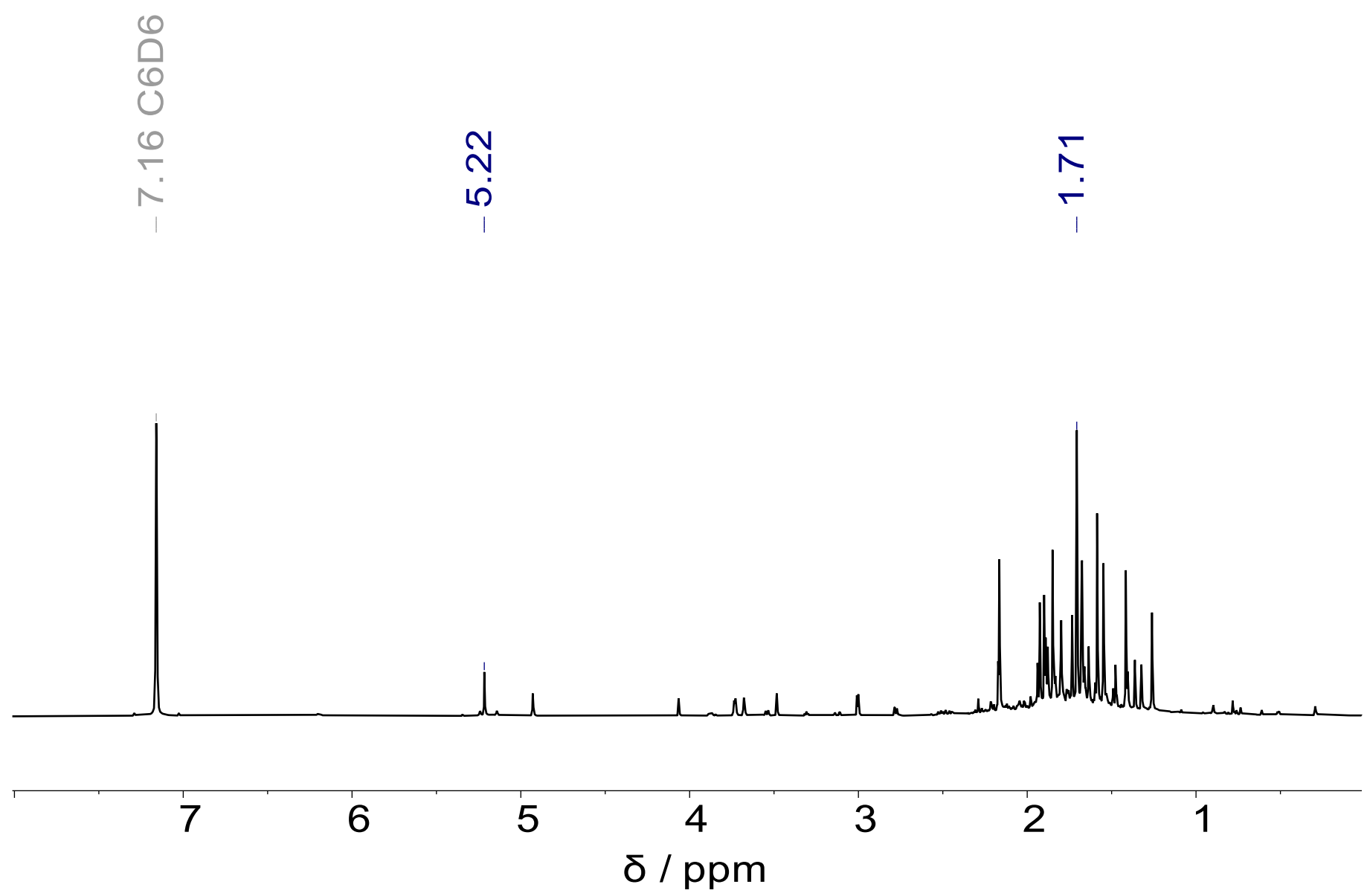

Figure S46. ^{1}H NMR spectrum (600 MHz, C_6D_6, Ar, 298 K) crude reaction solution Ir(acac)$_3$ synthesis, conditions A.

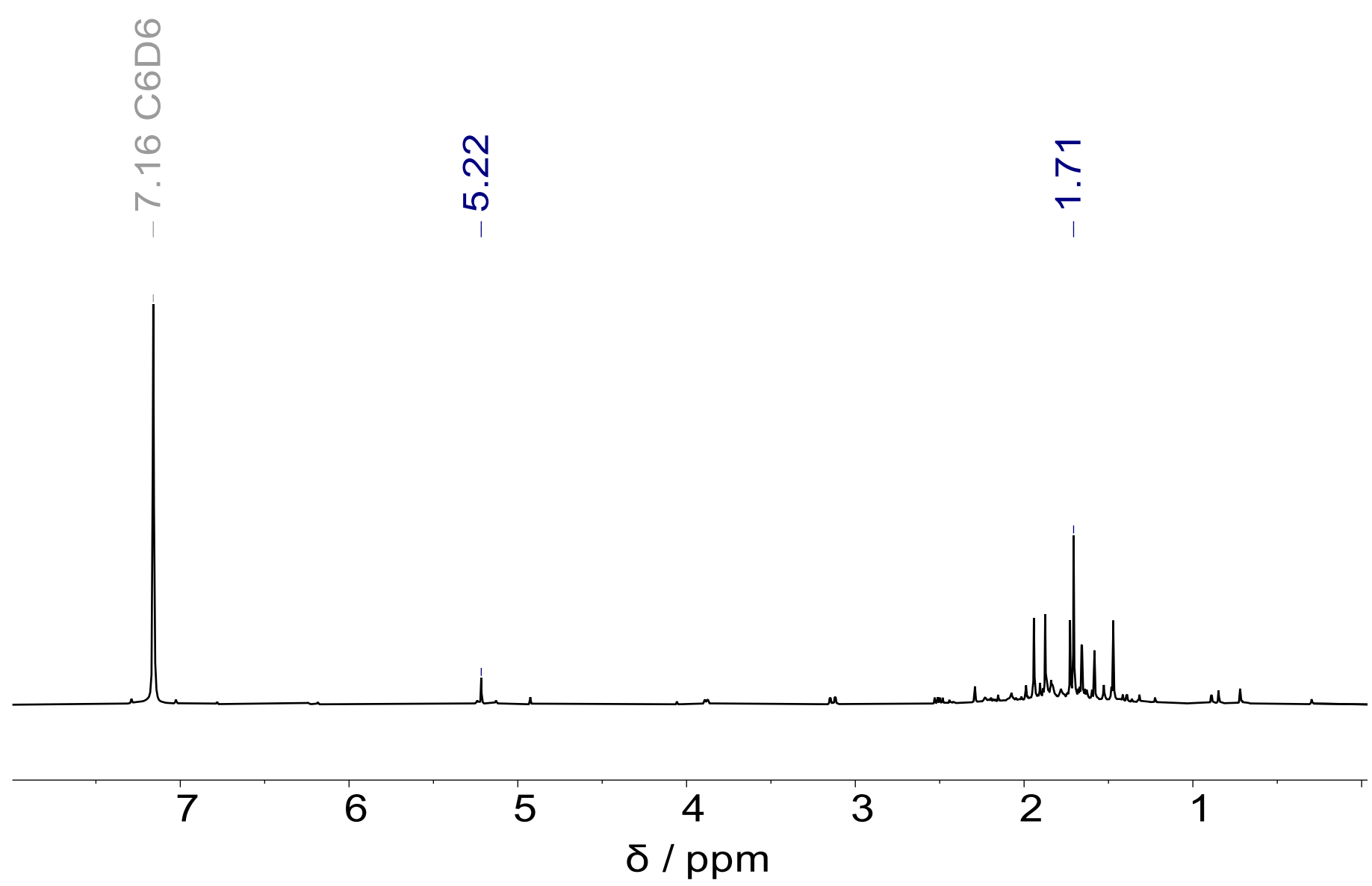

Figure S47. ^{1}H NMR spectrum (600 MHz, C_6D_6, Ar, 298 K) crude reaction solution Ir(acac)$_3$ synthesis, conditions B.

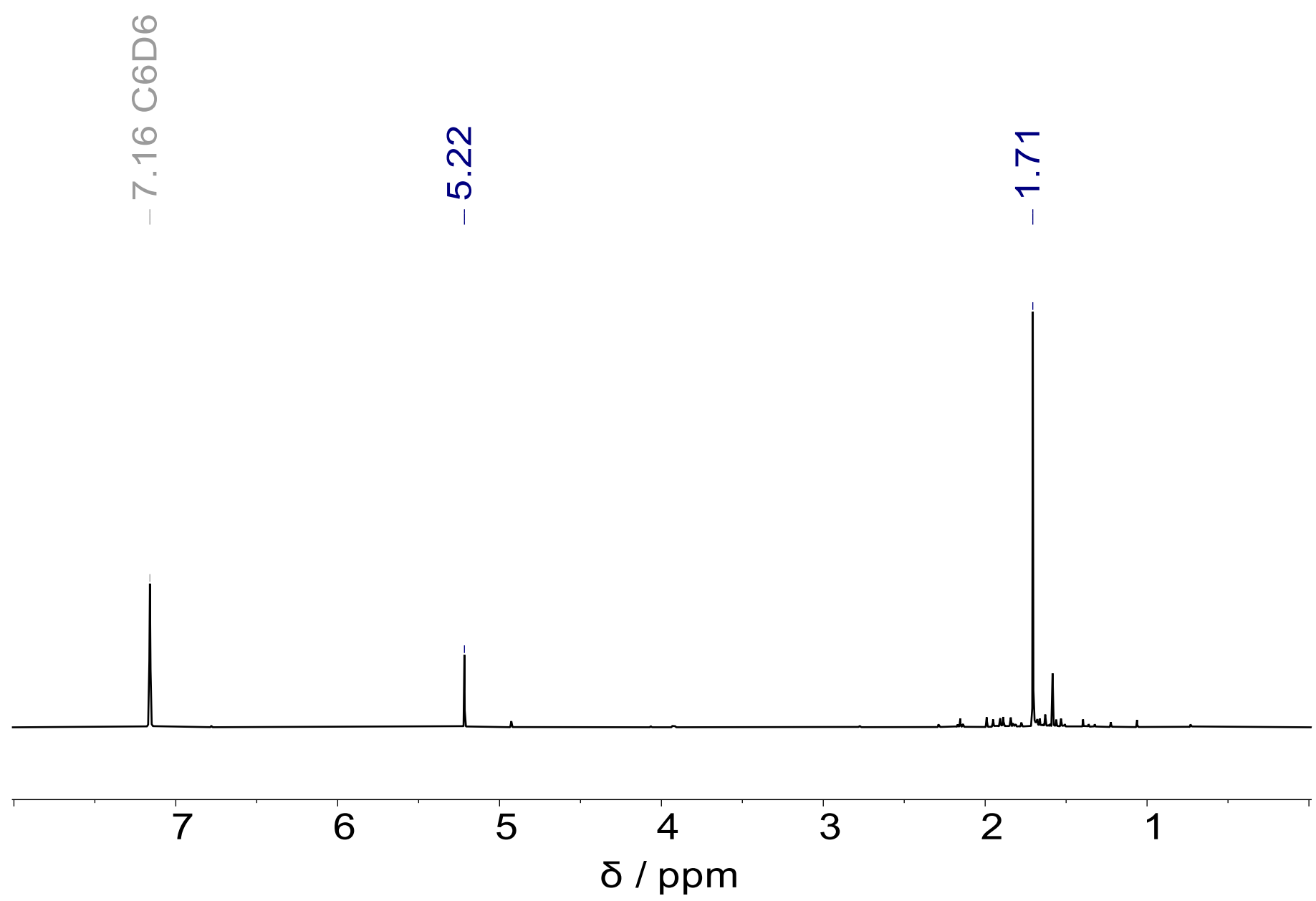

Figure S48. ^{1}H NMR spectrum (600 MHz, C_6D_6, Ar, 298 K) crude reaction solution $Ir(acac)_3$ synthesis, conditions C.

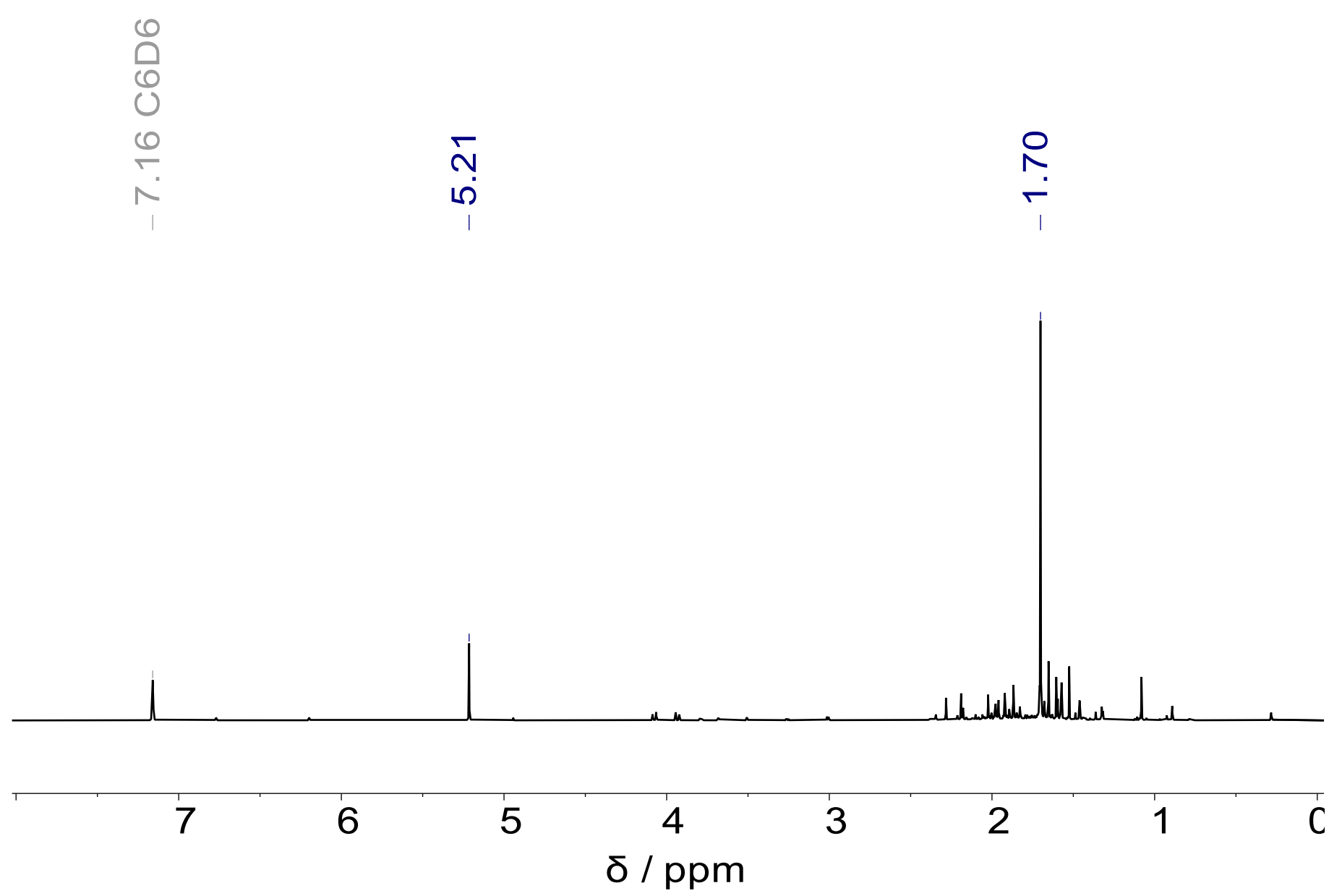

Figure S49. ^{1}H NMR spectrum (500 MHz, C_6D_6, Ar, 298 K) crude reaction solution $Ir(acac)_3$ synthesis, conditions D.

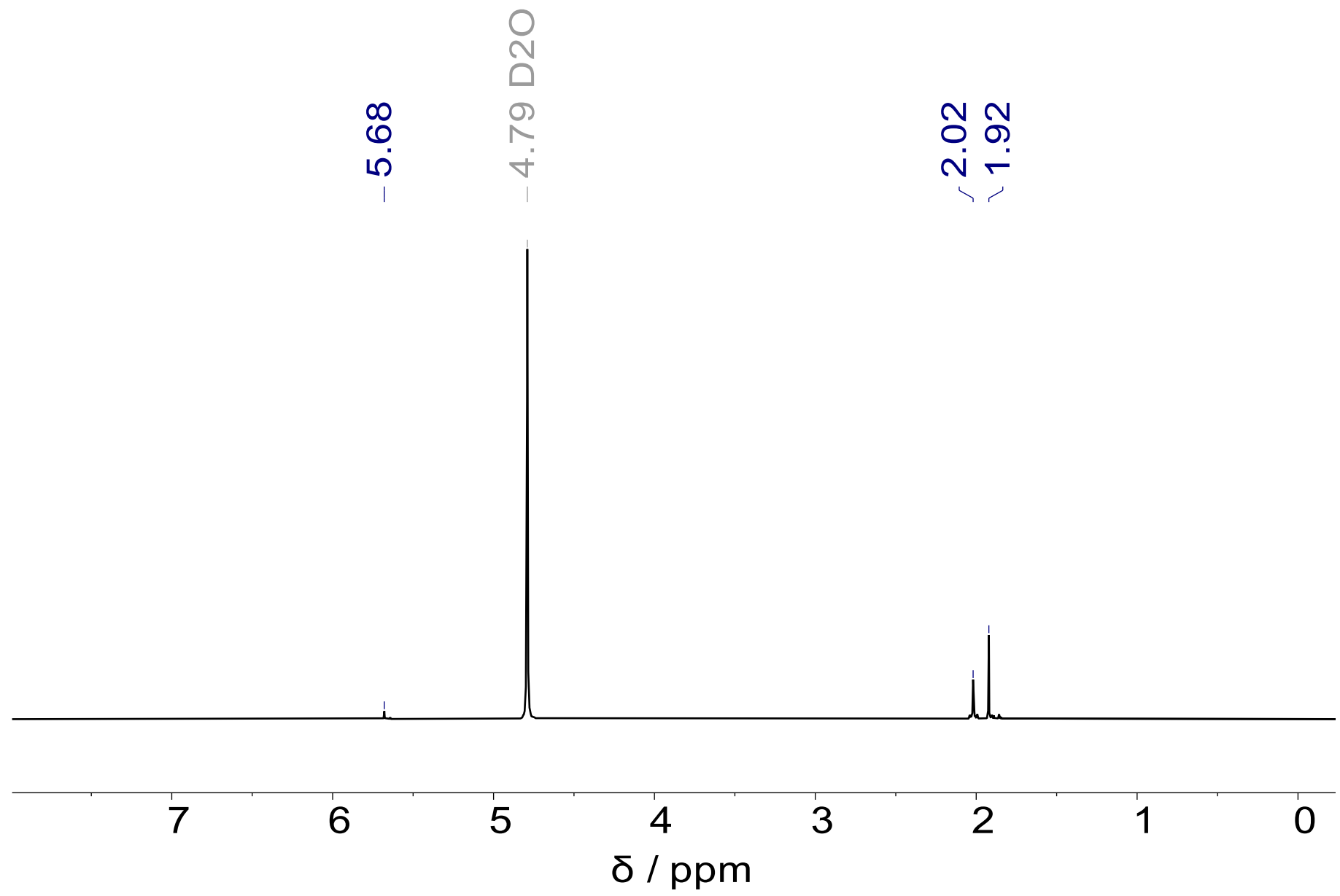

Figure S50. ^{1}H NMR spectrum (600 MHz, D_2O, Ar, 298 K) crude reaction solution Ir(acac)$_3$ synthesis, conditions E. Crude reaction solution separated from insoluble Ir(acac)$_3$ by cannula filtration. Solvent removed in vacuo from crude reaction solution and resulting oil redissolved in D_2O.

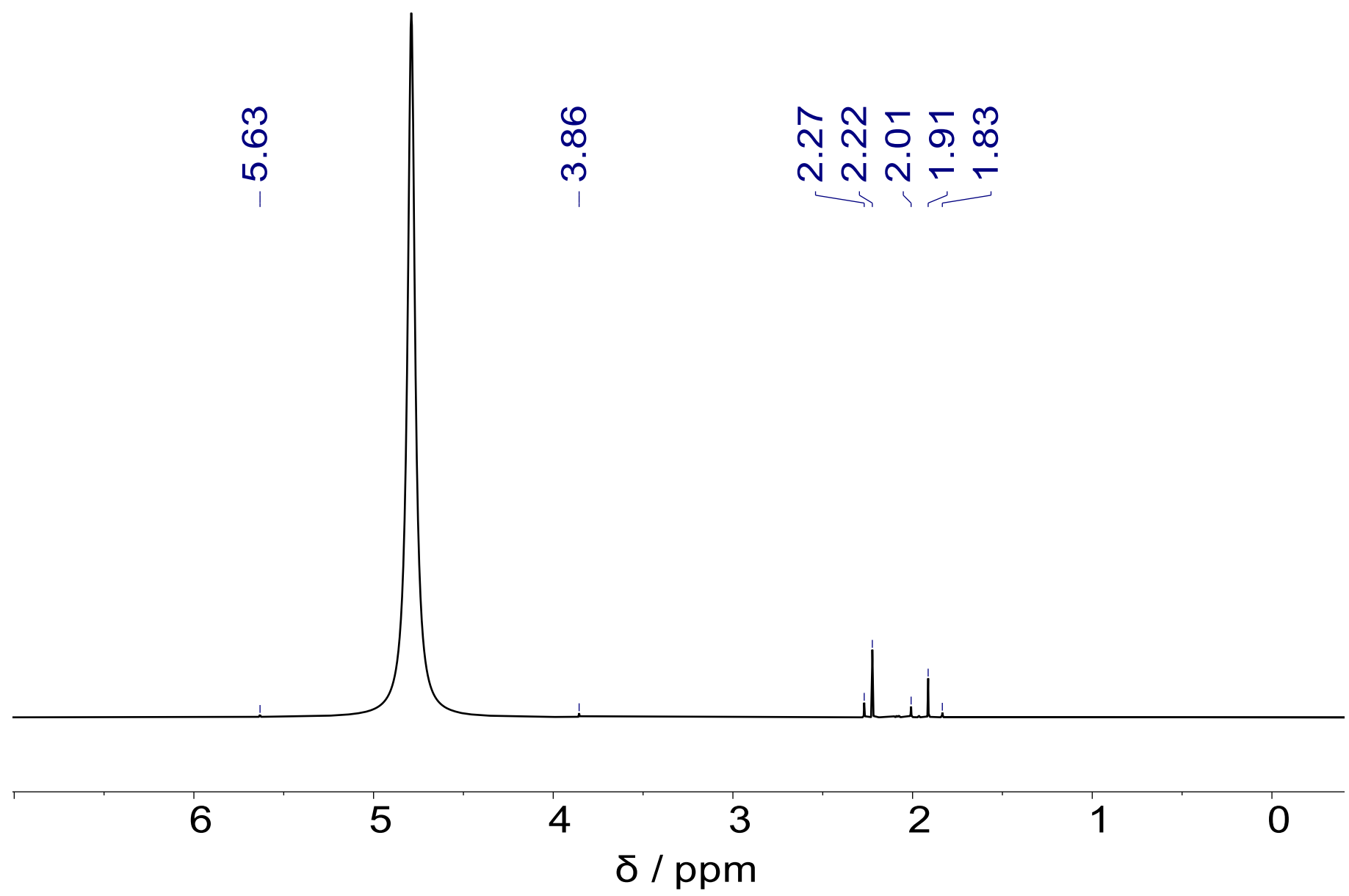

Figure S51. ^{1}H NMR spectrum (600 MHz, H_2O, Ar, 298 K) of crude reaction solution Ir(acac)$_3$ synthesis, conditions F. Crude reaction solution separated from insoluble Ir(acac)$_3$ by cannula filtration. Resonances associated with acetylacetone observed at δ 3.86 and δ 2.27 ppm (keto form) and δ 5.63 and δ 2.01 ppm (enol form, note: shifted ~0.1 ppm upfield from shifts observed in free acetylacetone).

7.3. The synthesis of $Ir(acac)_3$ from a 96-hour argon aged aqueous solution of $K_2[Ir(OH)_6]$

7.3.1. Experimental protocol

$K_2[Ir(OH)_6]$ (30 mg, 0.081 mmol) was dissolved in water (3 mL) under argon and left for 96 hours. Acetylacetone was added (4 equiv., 0.32 mmol, 35 μL) and the resulting solution was heated at 50 °C for ten days under argon. The resulting water-insoluble yellow precipitate was separated from the crude reaction mixture by cannula filtration and dried under vacuum to yield $Ir(acac)_3$ as a yellow powder (16 mg, 40 %).

7.3.2. NMR spectra

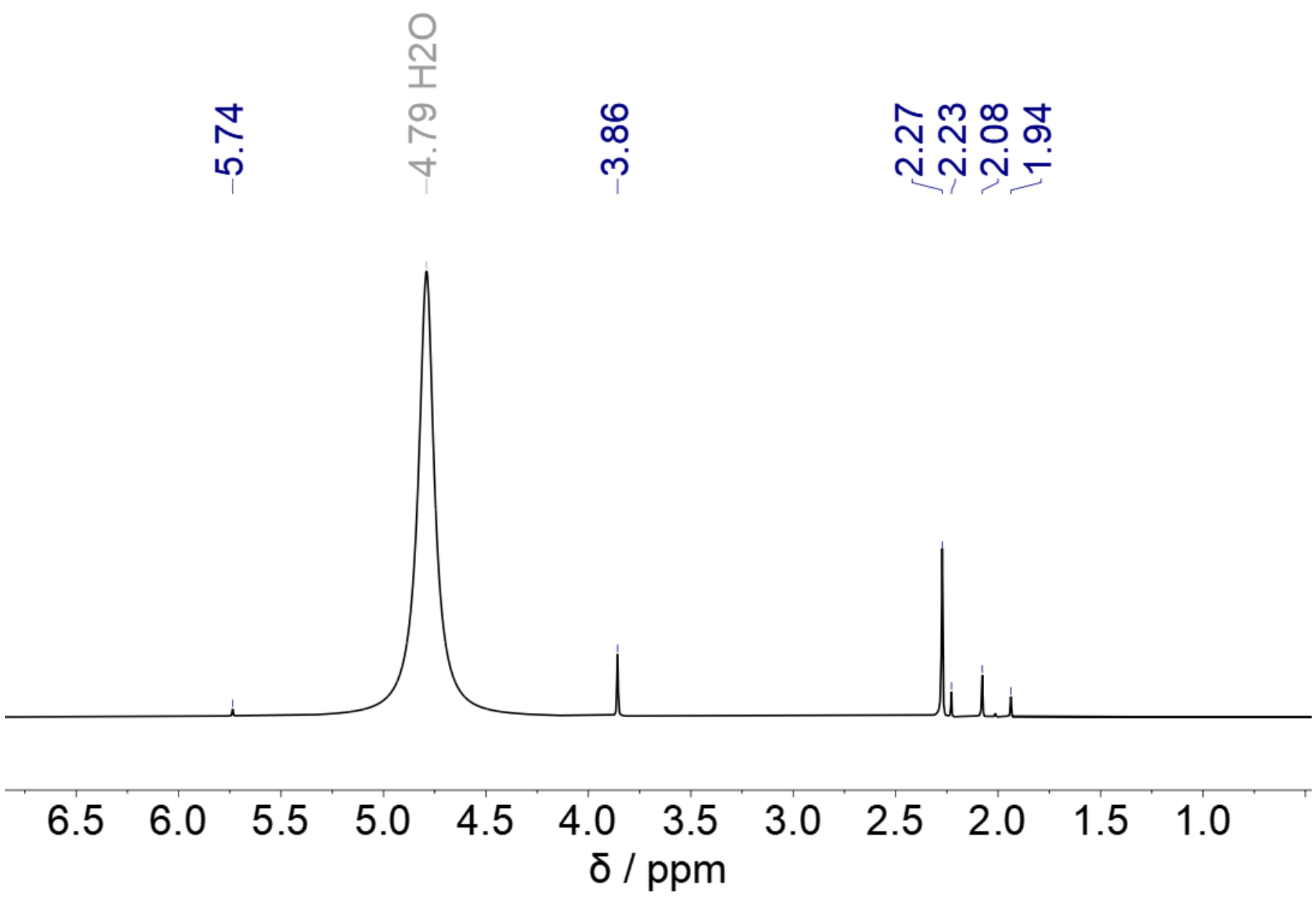

Figure S52. ^1H NMR spectrum (500 MHz, 298 K, H_2O) of crude reaction mixture generated from synthesis of $Ir(acac)_3$ from 96-hour aged solution of $K_2[Ir(OH)_6]$ under argon. Note product $Ir(acac)_3$ insoluble in water reaction solvent.

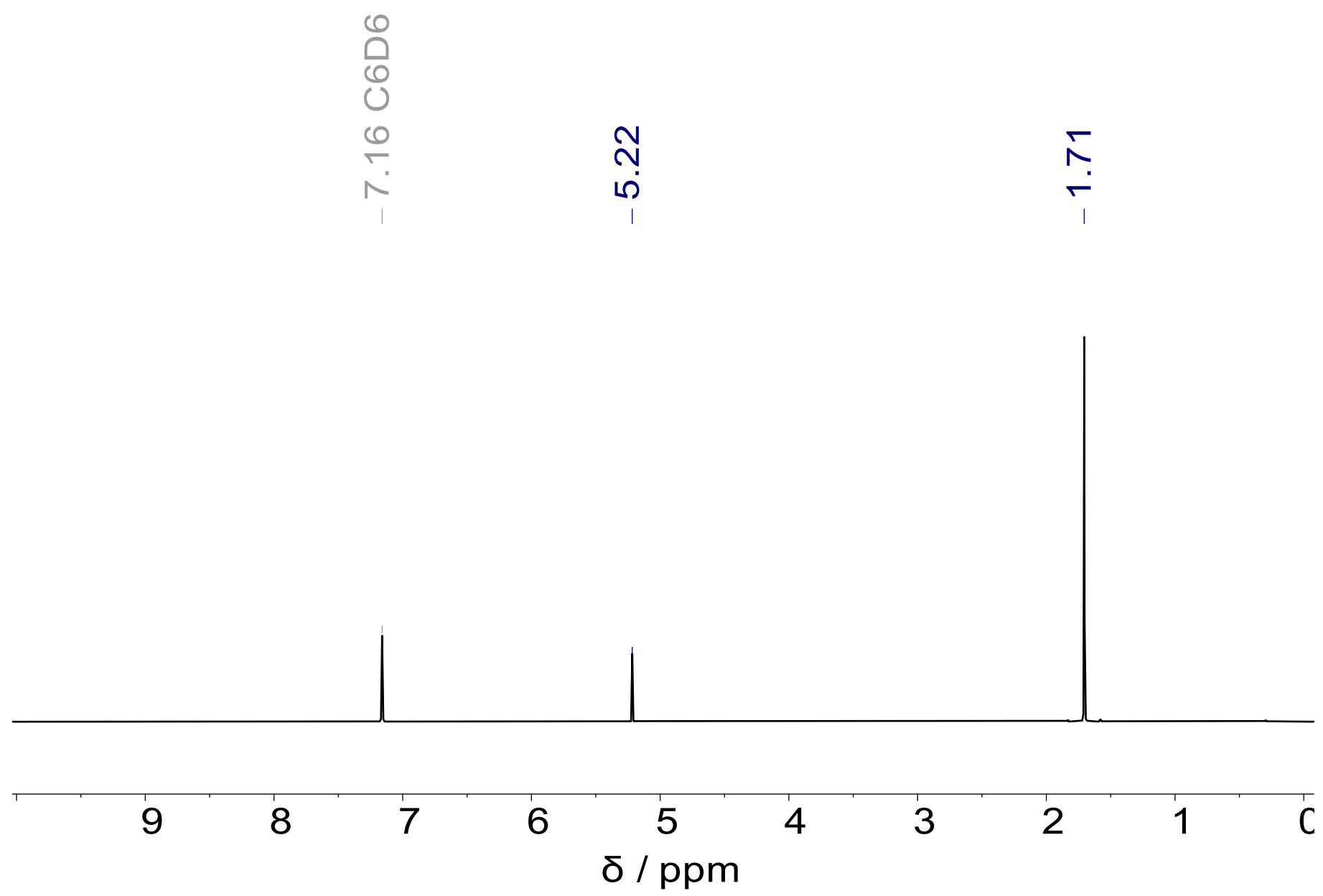

Figure S53. ^{1}H NMR spectrum (500 MHz, 298 K, C_6D_6) of isolated $Ir(acac)_3$ generated from synthesis of $Ir(acac)_3$ from 96-hour aged solution of $K_2[Ir(OH)_6]$ under argon.

8.0. The temporal solution-state behaviour of $K[Ir(C_2O_4)_2(H_2O)_2]$

Over five days, aqueous solutions of $K[Ir(C_2O_4)_2(H_2O)_2]$ show a change in speciation, demonstrated by ESI-MS. Growth in of adducts of $[Ir(C_2O_4)_3]^{3-}$ observed. An oxalate transfer process is hypothesised, as described previously for chromium bis(oxalate) complexes.[15]

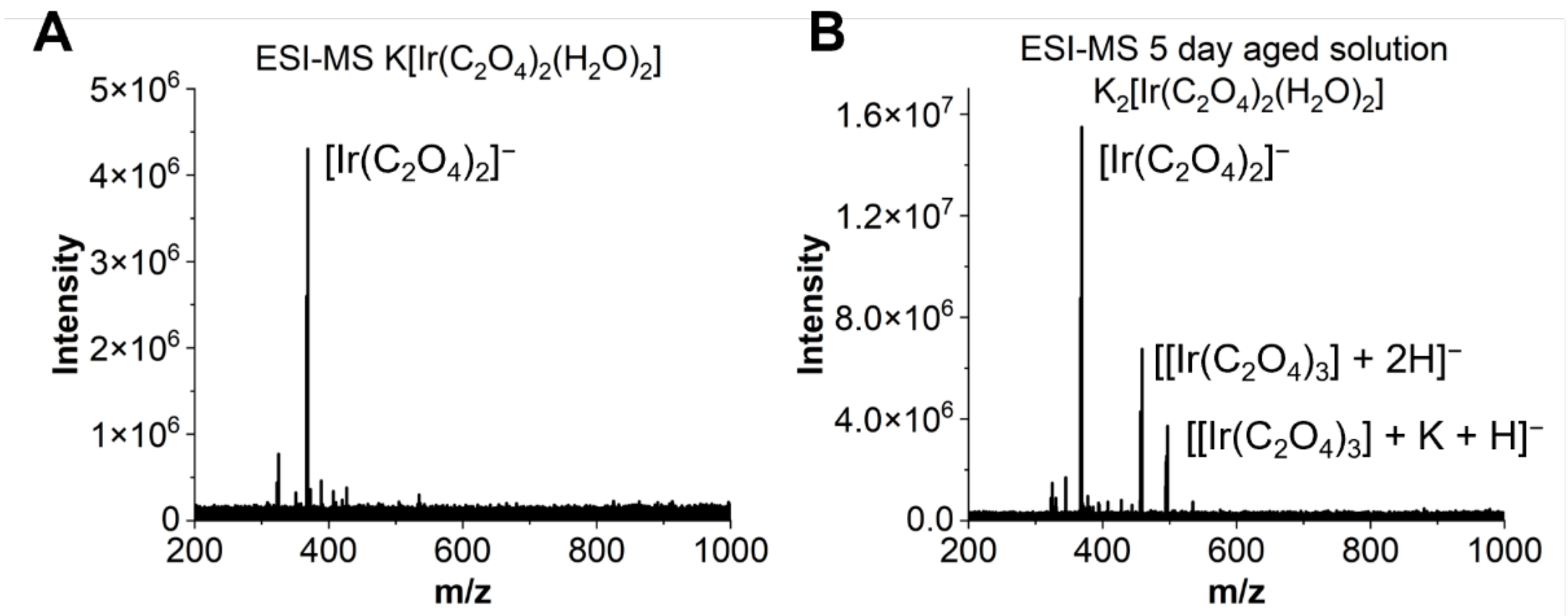

Figure S54. Mass spectra collected by ESI-MS of $K[Ir(C_2O_4)_2(H_2O)_2]$ immediately post dissolution in water (**A**) and after five days stored sealed in water in air (**B**).

9.0. Single-crystal diffraction data

9.1. Experimental details

9.1.1. X-ray diffraction

Single-crystal X-Ray diffraction data were collected on a Rigaku Synergy-S diffractometer with Cu-Kα (λ = 1.54184 Å) radiation equipped with a nitrogen gas Oxford Cryosystems Cryostream unit[16] at the University of York. Diffraction images from raw frame data were reduced using the CrysAlisPro suite of programmes. The structures were solved using SHELXT[17] and refined by full convergence on F^2 against all independent reflections by full matrix least-squares using SHELXL[18] (version 2018/3) through the Olex2 GUI.[19] All non-hydrogen atoms were refined anisotropically and hydrogen atoms were geometrically placed and allowed to ride on their parent atoms. Distances and angles were calculated using the full covariance matrix.

9.1.2. Neutron diffraction

The KOALA2 instrument stands at the end of guide position on TG-3, a thermal neutron supermirror guide in the neutron guide hall at the OPAL nuclear reactor at ANSTO, Lucas Heights, NSW Australia. Data were collected in the Laue mode - with the detector covering a full 360 degrees about the instrument phi axis by means of a neutron sensitive image plate. A total of 12 images for a single mounting of the rhombohedral crystal relative to the phi axis were measured for 83 minutes neutron beam exposure time per image, with an interframe rotation of 17 degrees between each pair of exposures. A typical image is shown below, and the program LaueG can be employed to index each frame (using the X-ray determined cell at the same temperature) to facilitate extraction of data for normalization and use in refinement.[20, 21] 9880 data in the wavelength range 0.7 ≤ λ ≤ 1.4 angstrom were extracted Rint = 6.1 (4.5) % wRint2 =7.9 (6.0) % GOOF = 1.3 (976 data > 4 sig) of which 1148 were unique. Rint = 13.8(12.3) % wRint2 =19.4 (18.6) % GOOF = 1.0 (for all data). The structure from the X-ray study was refined against the neutron diffraction data with the H atom site clearly defined in the difference map using CRYSTALS.[22] A free refinement on F of all atoms modelled with anisotropic displacement parameters converged to R = 0.061, Rw = 0.022 for 24 parameters, 602 data I ≥ 3σI with application of a suitable weighting scheme. Final residual densities of -1.55 and 1.45 (barn/fermi) in a featureless difference map and lie at background levels inconsistent with any unmodelled atomic sites.

Crystallographic data are available free of charge via the Cambridge Crystallographic Data Centre, under deposition numbers 2554247–2554252.

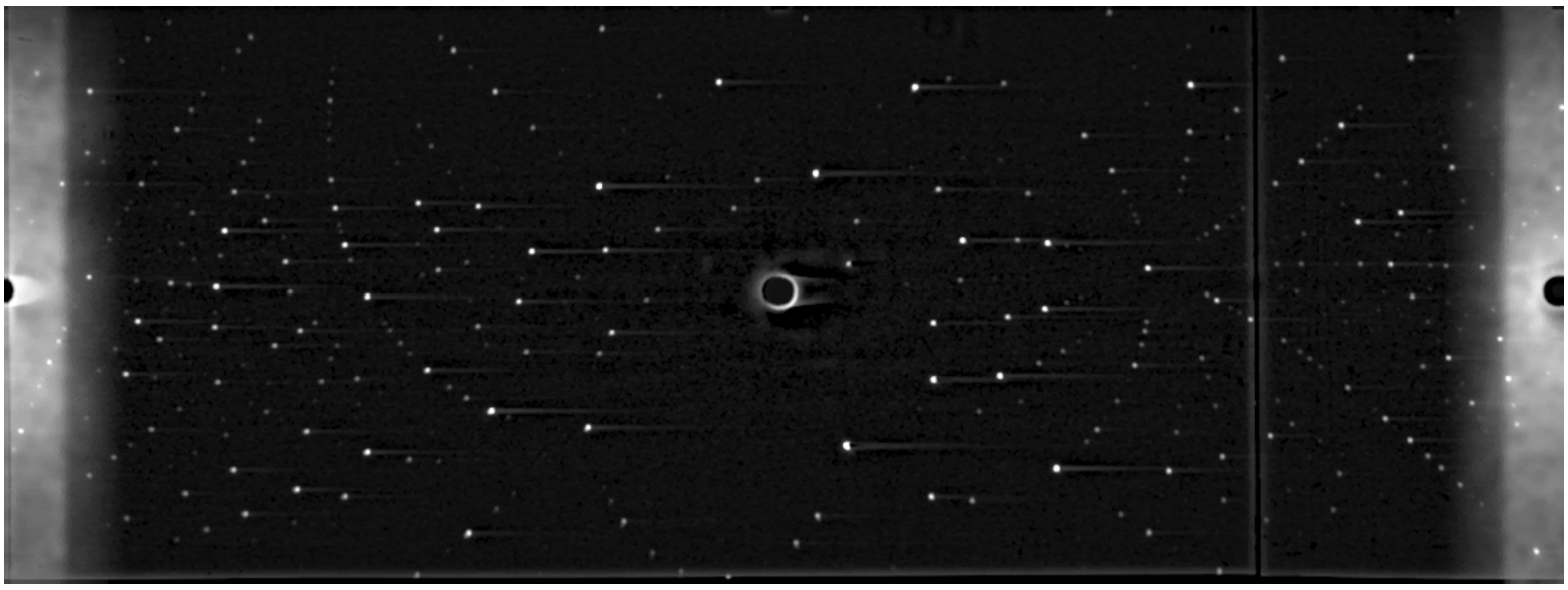

Figure S55. A selected neutron diffraction image.

9.2. $K_2[Ir(OH)_6]$

9.2.1. Supplementary crystallographic figures

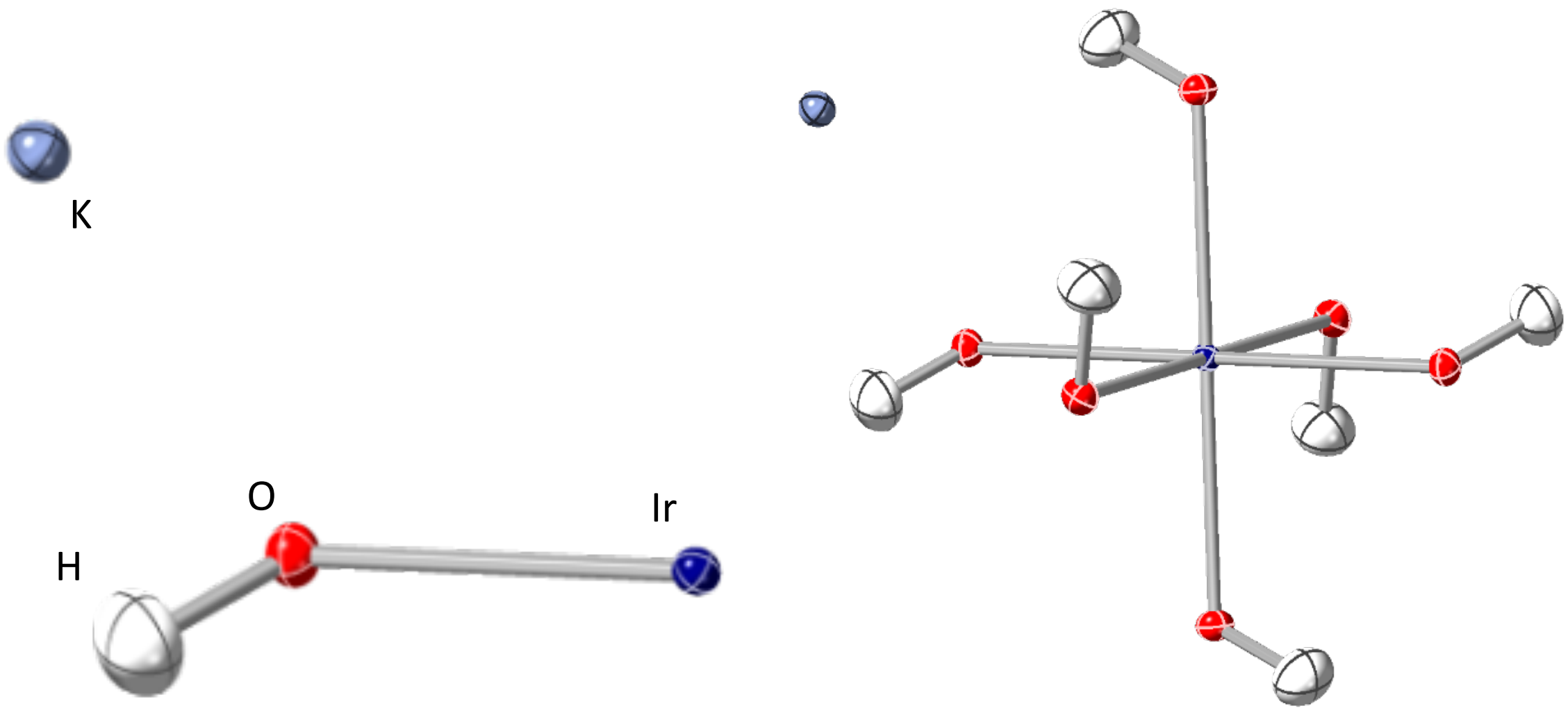

Figure S56. Asymmetric unit (left) and molecular structure (right) of $K_2[Ir(OH)_6]$ as determined by single-crystal neutron diffraction. Anisotropic displacement parameters are represented as 50 % probability ellipsoids. Selected bond lengths (Å): Ir–O 2.0137(3), O–H 0.9744(6). Selected bond angle (°): Ir–O–H 108.75(5).

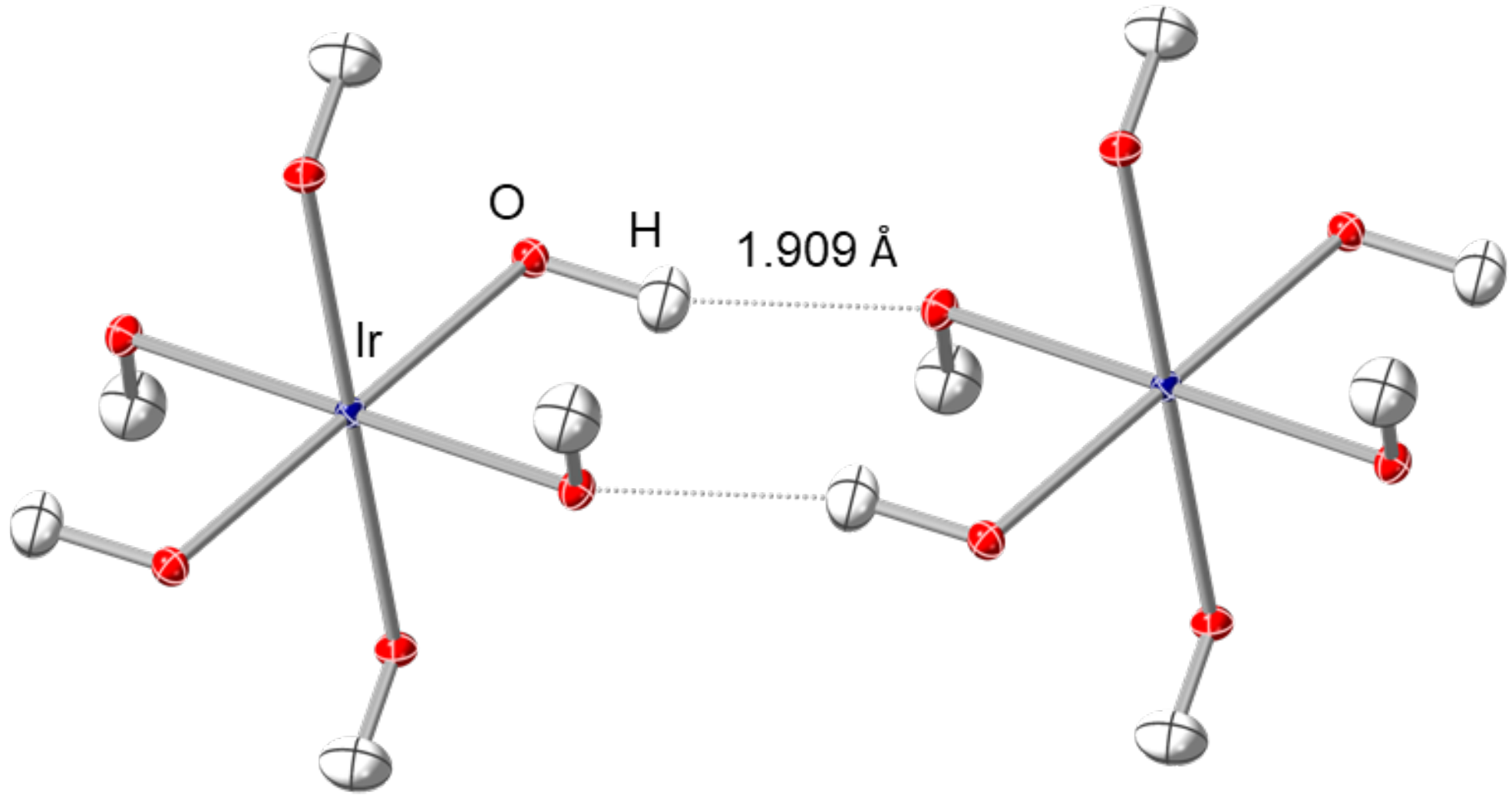

Figure S57. Hydrogen bonding between adjacent $[Ir(OH)_6]^{2-}$ octahedra with an O---H bond length of 1.909 Å as determined by single-crystal neutron diffraction data collected at 293 K. Potassium counterions omitted for clarity.

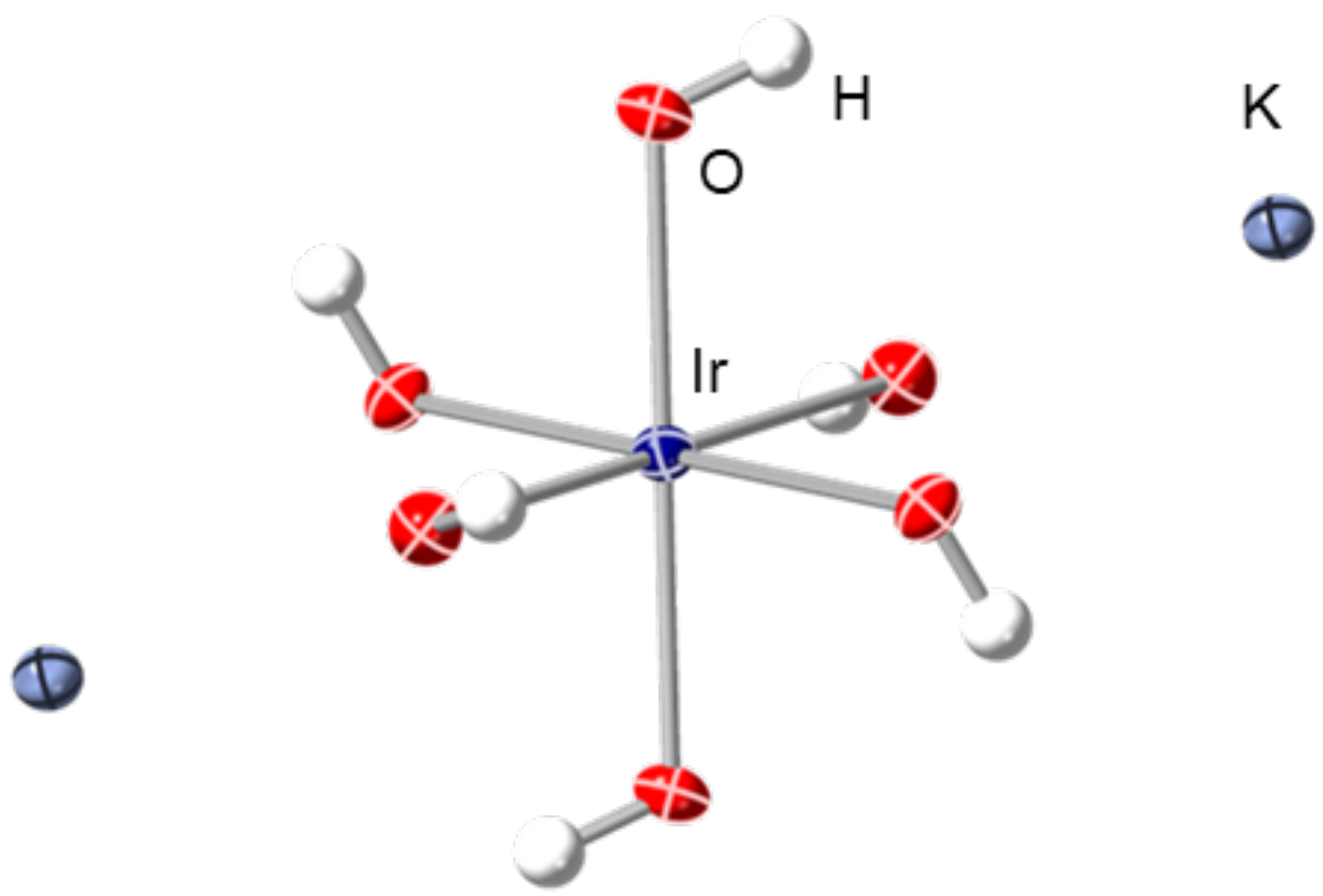

Figure S58. Molecular structure of $K_2[Ir(OH)_6]$ as determined by single-crystal X-ray diffraction, solved in the *R*-3 space group (R_1 = 2.02 %). Anisotropic displacement parameters are represented as 50 % probability ellipsoids. Hydrogen atoms represented as spheres of 0.2 Å radius. Bond lengths (Å) and angles (°): Ir–O: 2.016 (4), O–Ir–O 90.53 (17).

9.2.2. Crystallographic data table

Table S8. Selected crystallographic data for $K_2[Ir(OH)_6]$ collected by single-crystal X-ray and neutron diffraction.

	$K_2[Ir(OH)_6]$ (X-ray, 110 K)	$K_2[Ir(OH)_6]$ (neutron)	$K_2[Ir(OH)_6]$ (X-ray, 260 K)
CCDC number	2554250	2554252	2554251
Empirical formula	$H_6IrK_2O_6$	$H_6IrK_2O_6$	$H_6IrK_2O_6$
Formula weight	372.45	372.45	372.45
Temperature / K	110.00(10)	260	260
Crystal system	trigonal	trigonal	trigonal
Space group	*R*-3	*R*-3	*R*-3
a / Å	6.4026(2)	6.4026(2)	6.4243(1)
b / Å	6.4026(2)	6.4026(2)	6.4243(1)
c / Å	12.6887(5)	12.6887(5)	12.7430(2)
α / °	90	90	90
β / °	90	90	90
γ / °	120	120	120
Volume / $Å^3$	450.46(3)	450.46(3)	455.464(16)
Z	3	3	3
ρ_{calc} g / cm^3	4.119	4.119	4.074
μ / mm^{-1}	55.295	0.0	54.688
F(000)	507.0	93.0	507.0
Radiation	Cu Kα (λ = 1.54184)	NEUTRONS (λ = 0.70000)	Cu Kα (λ = 1.54184)
2Θ range for data collection / °	17.454 to 159.516	9.618 to 180	17.392 to 159.052
Index ranges	−7 ≤ h ≤ 7, −8 ≤ k ≤ 6, −15 ≤ l ≤ 15	-15 ≤ h ≤ 15, -15 ≤ k ≤ 15, −24 ≤ l ≤ 22	−7 ≤ h ≤ 8, −7 ≤ k ≤ 7, −15 ≤ l ≤ 15
Reflections collected	2693	9880	2893
Independent reflections	208 [R_{int} = 0.0506, R_{sigma} = 0.0160]	1148 [R_{int} = 0.1381, R_{sigma} = 0.1631]	215 [R_{int} = 0.0484, R_{sigma} = 0.0154
Data / restraints / parameters	208/1/19	1148/0/24	215/0/16
Goodness-of-fit on F^2	1.300	1.392	1.287
Final R indexes [I>=2σ(I)]	R_1 = 0.0202 wR_2 = 0.0569	R_1 = 0.0610 wR_2 = 0.0219	R_1 = 0.0147 wR_2 = 0.0412
Final R indexes [all data]	R_1 = 0.0202 wR_2 = 0.0569	R_1 = 0.0450 wR_2 = 0.0209	R_1 = 0.0147 wR_2 = 0.0412
Largest diff. peak/ hole / e $Å^{-3}$	0.80/−1.63	1.45 / −1.55	0.55 / −1.08

9.3. Ir(acac)$_3$

9.3.1. Supplementary crystallographic figures

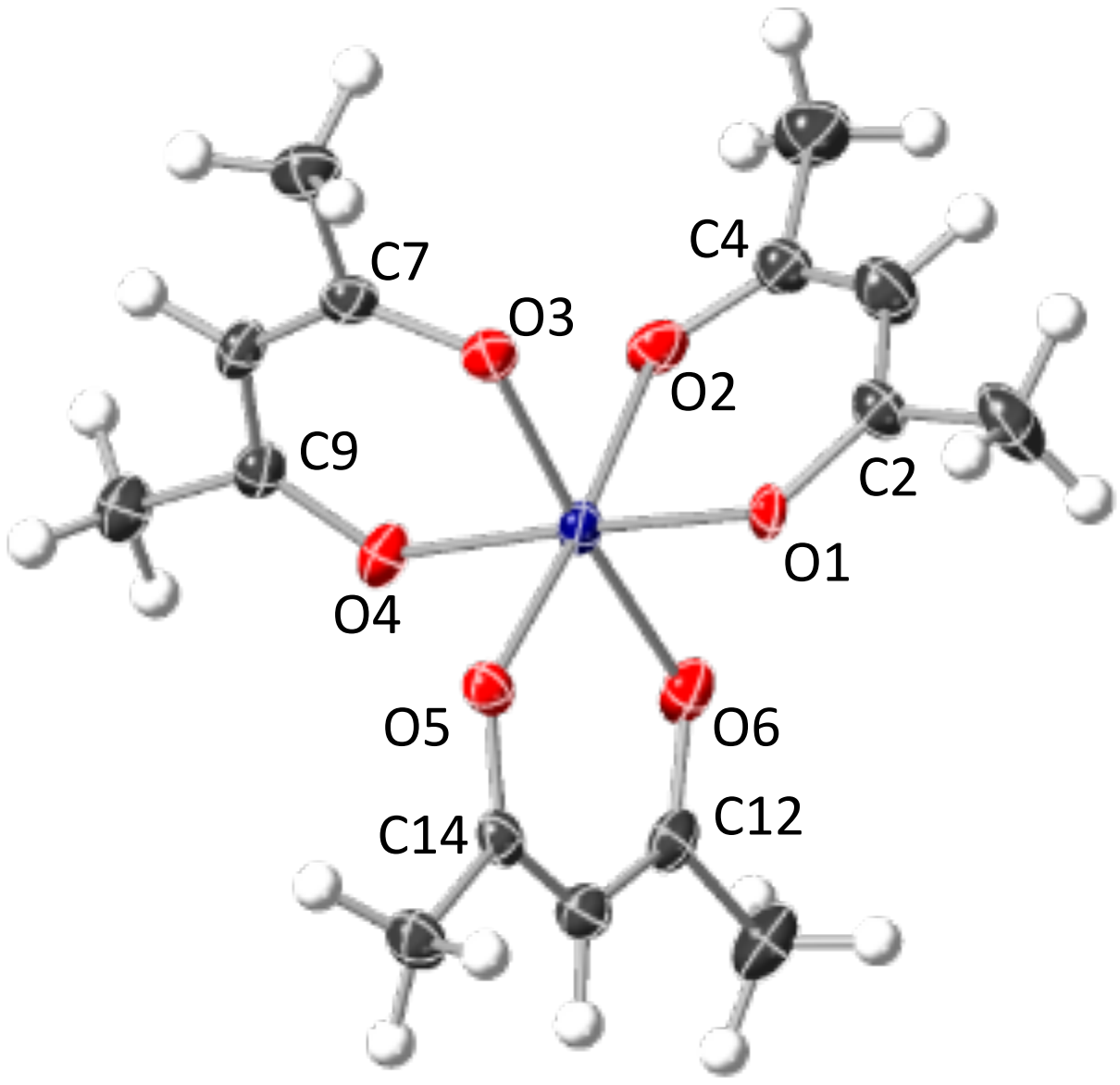

Figure S59. Asymmetric unit of Ir(acac)$_3$ as determined by single-crystal X-ray diffraction. Anisotropic displacement parameters are represented as 50 % probability ellipsoids. Hydrogen atoms represented as spheres of 0.2 Å radius. Selected bond lengths (Å): Ir–O(1) 2.030(7), Ir–O(2) 2.021(7), Ir–O(3) 2.0130(19), Ir–O(4) 2.0137(18), Ir–O(5) 2.0170(16), Ir–O(6): 2.0205; O(1)–C(2) 1.283(8), O(2)–C(4) 1.281(8), O(3)–C(7) 1.281(3), O(4)–C(9) 1.281(3), O(5)–C(14) 1.281(3), O(6)–C(12) 1.278(3).

9.3.2. Crystallographic data table

Table S9. Selected crystallographic data for $Ir(acac)_3$ collected by single-crystal X-ray diffraction.

	$Ir(acac)_3$
CCDC number	2554247
Empirical formula	$C_{15}H_{21}IrO_6$
Formula weight	489.52
Temperature / K	110.05(10)
Crystal system	monoclinic
Space group	$P2_1/c$
a / Å	13.6688(1)
b / Å	7.4236(1)
c / Å	16.3287(1)
α / °	90
β / °	98.889(1)
γ / °	90
Volume / $Å^3$	1637.00(3)
Z	4
ρ_{calc} g / cm^3	1.986
μ / mm^{-1}	16.026
F(000)	944.0
Radiation	Cu Kα (λ = 1.54184)
2 Θ range for data collection / °	10.968 to 134.132
Index ranges	$-16 \le h \le 16$, $-8 \le k \le 8$, $-16 \le l \le 19$
Reflections collected	20485
Independent reflections	2924 [R_{int} = 0.0355, R_{sigma} = 0.0215]
Data / restraints / parameters	2924/101/272
Goodness-of-fit on F^2	1.045
Final R indexes [I>=2σ(I)]	R_1 = 0.0153, wR_2 = 0.0352
Final R indexes [all data]	R_1 = 0.0165, wR_2 = 0.0357
Largest diff. peak/ hole / e $Å^{-3}$	0.56/−0.47

9.4. *Cis-/trans*-K[Ir(C_2O_4)$_2$(H_2O)$_2$]

9.4.1. Supplementary crystallographic figures

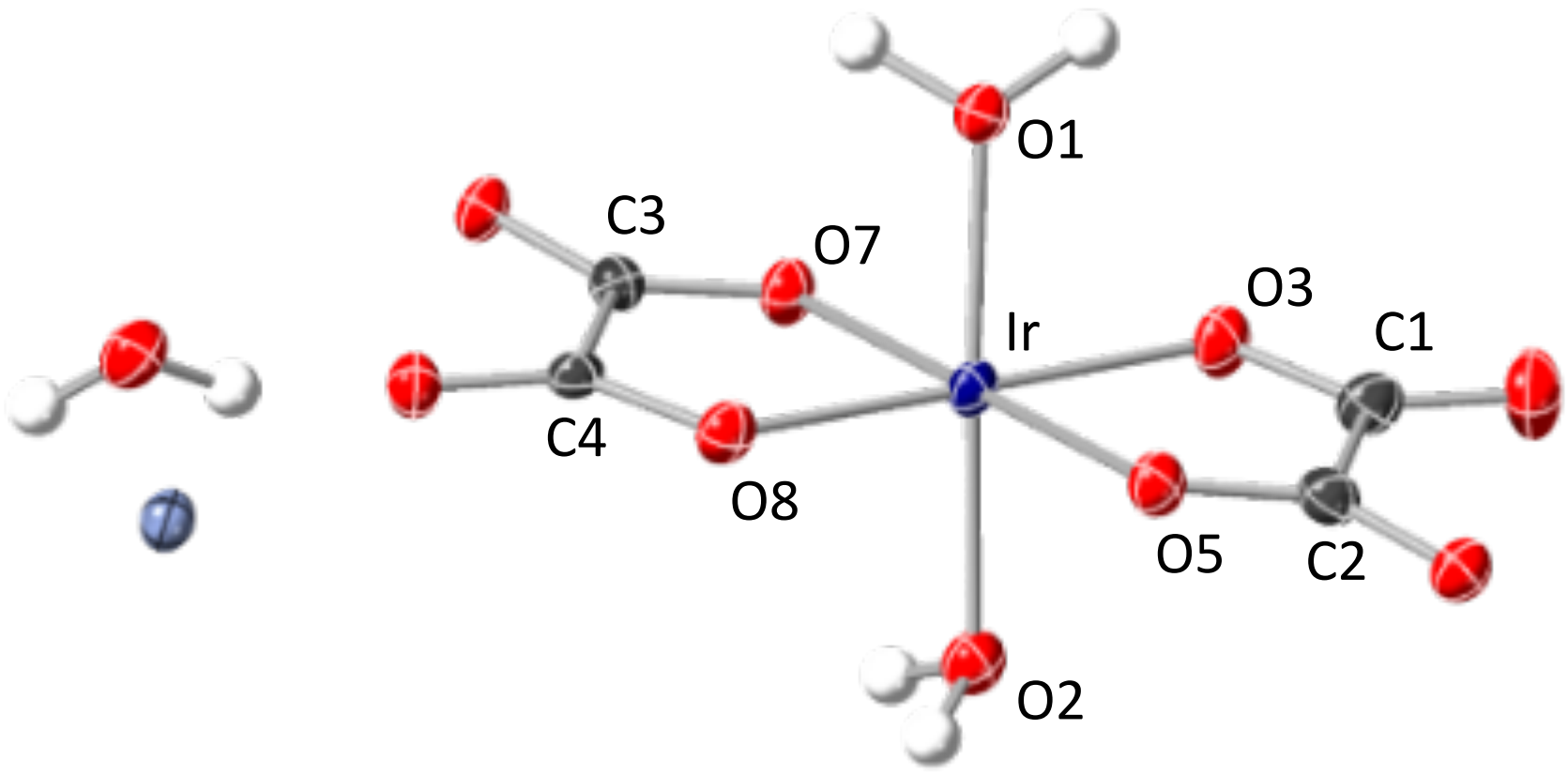

Figure S60. Asymmetric unit of *trans*-K[Ir(C_2O_4)$_2$(H_2O)$_2$] as determined by single-crystal X-ray diffraction. Anisotropic displacement parameters are represented as 50 % probability ellipsoids. Hydrogen atoms represented as spheres of 0.2 Å radius. Selected bond lengths (Å): Ir(1)–O(1) 2.070(3), Ir(1)–O(2) 2.050(3), Ir(1)–O(3) 2.040(3), Ir(1)–O(5) 2.044(3), Ir(1)–O(7) 2.040(3), Ir(1)–O(8) 2.034(3); O(3)–C(1) 1.283(6), O(5)–C(2) 1.284(6), O(7)–C(3) 1.273(5), O(8)–(C4) 1.297(5). Selected bond angles (°): O(3)–Ir(1)–O(5) 80.97(13), O(7)–Ir(1)–O(8) 81.54(12), O(5)–Ir(1)–O(7) 179.84, O(3)–Ir(1)–O(8) 179.06(13); O(1)–Ir(1)–O(2) 177.60(12).

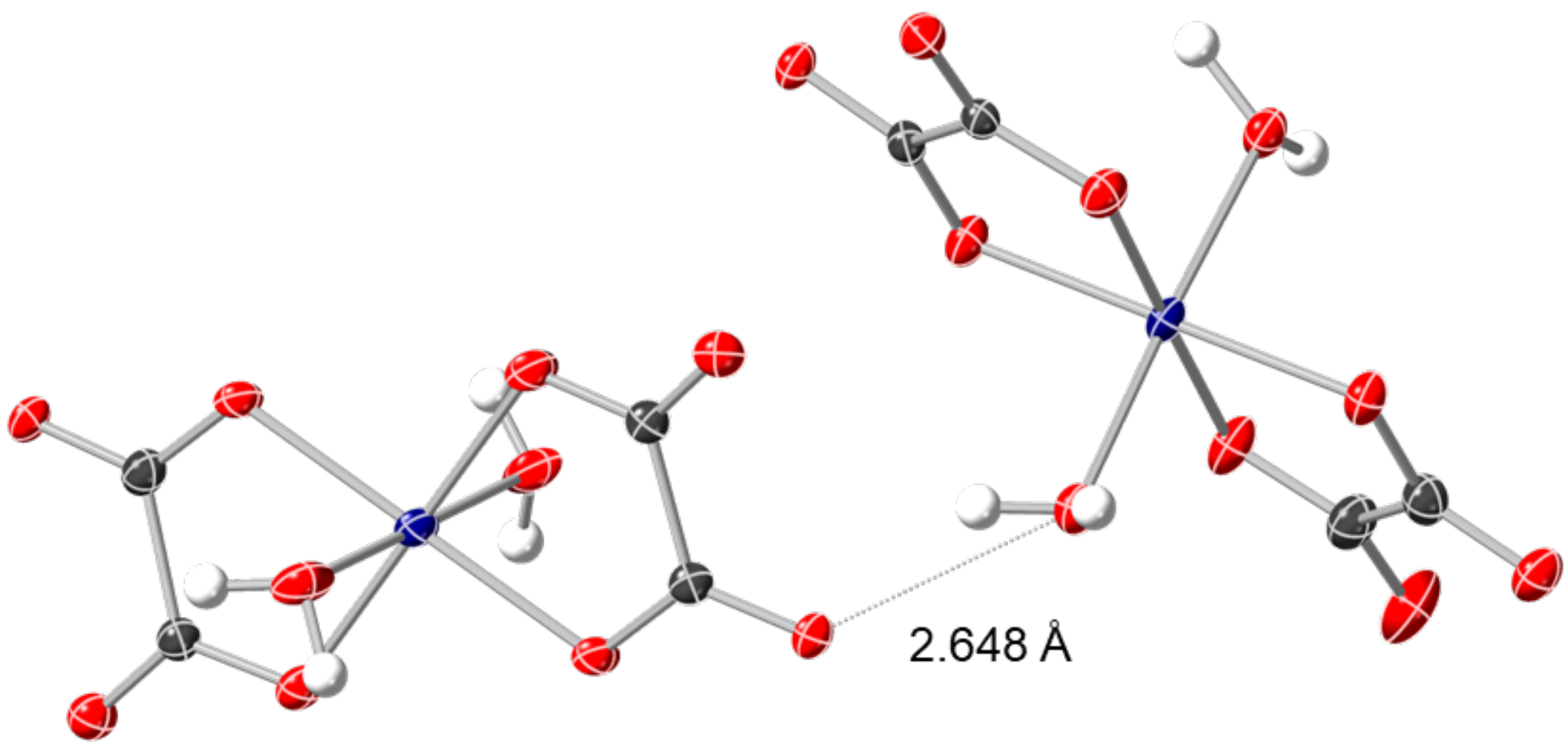

Figure S61. Hydrogen bonding between coordinated water ligands and terminal oxygens of oxalate ligands on adjacent octahedra of *trans*-K[Ir(C_2O_4)$_2$(H_2O)$_2$]. O---O bond distances shown. Anisotropic displacement parameters are represented as 50 % probability ellipsoids. Hydrogen atoms represented as spheres of 0.2 Å radius.

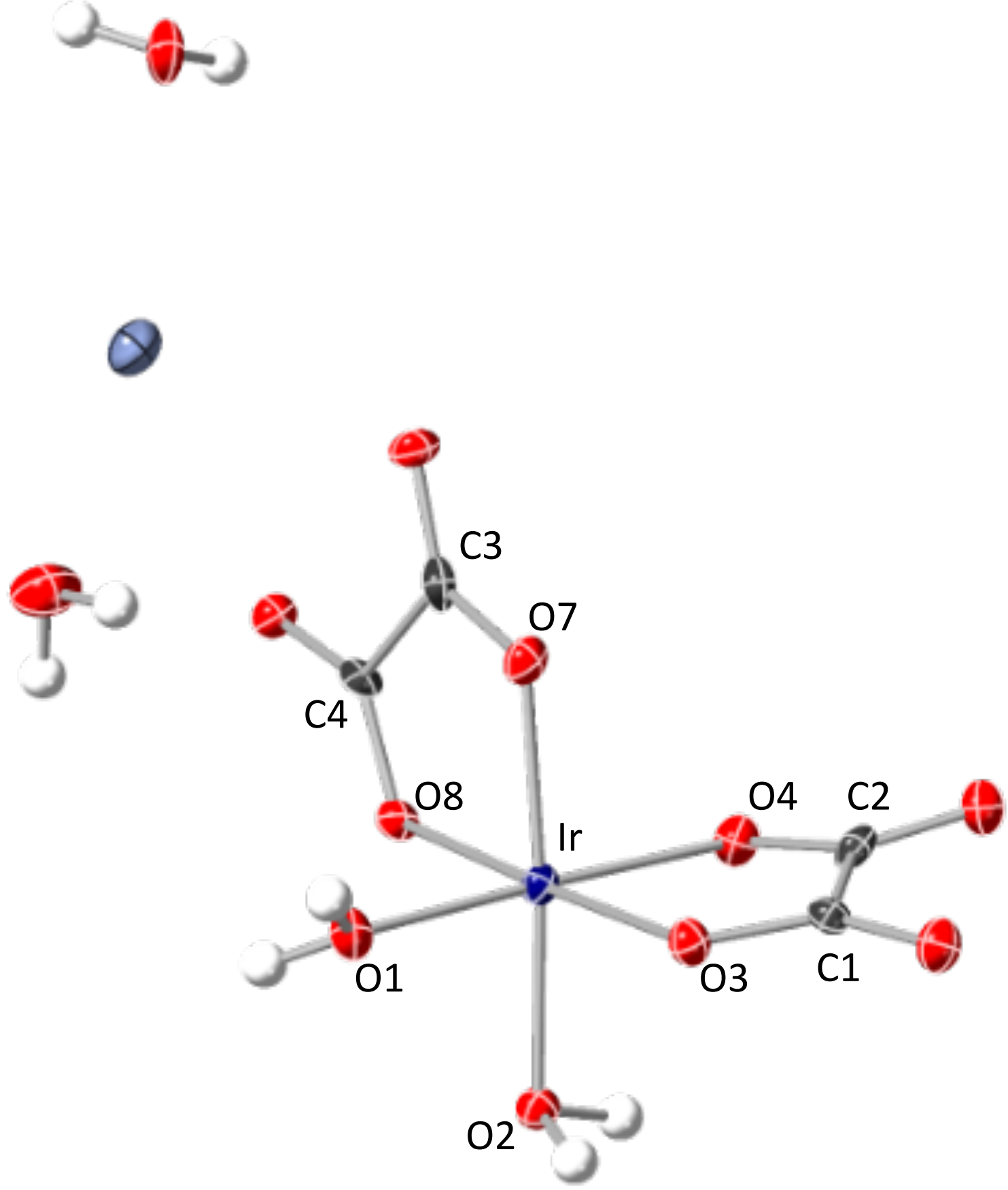

Figure S62. Asymmetric unit of *cis*-K[Ir(C_2O_4)$_2$(H_2O)$_2$] as determined by single-crystal X-ray diffraction. Anisotropic displacement parameters are represented as 50 % probability ellipsoids. Hydrogen atoms represented as spheres of 0.2 Å radius. Selected bond lengths (Å): Ir–O(1) 2.043(5), Ir–O(2) 2.042(5), Ir–O(3) 2.048(4), Ir–O(4) 2.043(4), Ir–O(7) 2.027(5), Ir–O(8) 2.048(4); O(3)–C(1) 1.300(8), O(4)–C(2) 1.291(8), O(7)–C(3) 1.295(8), O(8)–C(4) 1.289(8). Selected bond angles (^{o}): O(3)–Ir–O(4) 82.16(18), O(7)–Ir–O(8) 81.40(17), O(3)–Ir–O(8) 177.65(16), O(3)–Ir–O(7) 97.30(18), O(4)–Ir–O(8) 95.84(18), O(1)–Ir–O(4) 176.76(16), O(2)–Ir–O(7) 175.11.(17).

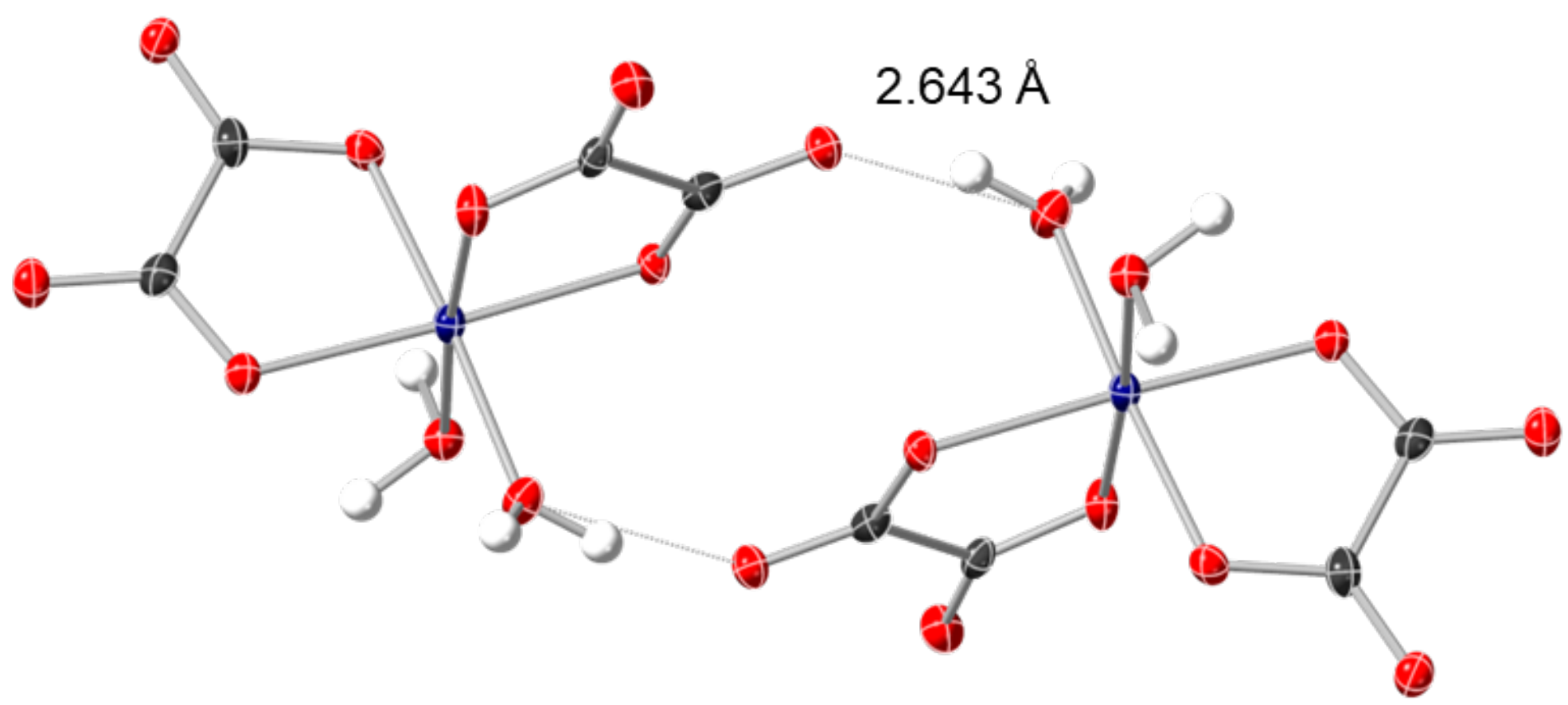

Figure S63. Hydrogen bonding between coordinated water ligands and terminal oxygens of oxalate ligands on adjacent octahedra of *cis*-K[Ir(C_2O_4)$_2$(H_2O)$_2$].. O---O bond distances shown. Anisotropic displacement parameters are represented as 50 % probability ellipsoids. Hydrogen atoms represented as spheres of 0.2 Å radius.

9.4.2. Crystallographic data table

Table S60. Selected crystallographic data for *cis*- and *trans*-K[Ir(C_2O_4)$_2$(H_2O)$_2$] collected by single-crystal X-ray diffraction.

	trans-K[Ir(C_2O_4)$_2$(H_2O)$_2$]	*cis*-K[Ir(C_2O_4)$_2$(H_2O)$_2$]
CCDC number	2554249	2554248
Empirical formula	$C_{4.75}H_6IrKO_{10.75}$	$C_4H_8IrKO_{12}$
Formula weight	467.40	479.40
Temperature / K	109.99(13)	110.0(2)
Crystal system	triclinic	triclinic
Space group	P-1	P-1
a / Å	6.26020(10)	6.1437(3)
b / Å	12.3561(2)	7.7367(4)
c / Å	14.5247(2)	12.2030(9)
α / °	104.686(2)	75.459(5)
β / °	96.3000(10)	88.291(5)
γ / °	97.8280(10)	72.203(5)
Volume / Å^3	1064.41	533.89(6)
Z	4	2
ρ_{calc} g / cm^3	2.917	2.982
μ / mm^{-1}	28.372	28.400
F(000)	870.0	448.0
Radiation	Cu Kα (λ = 1.54184)	Cu Kα (λ = 1.54184)
2 Θ range for data collection / °	11.096 to 136.498	7.494 to 136.434
Index ranges	−7 ≤ h ≤ 7, −14 ≤ k ≤ 14, −17 ≤ l ≤ 17	−7 ≤ h ≤ 7, −8 ≤ k ≤ 9, −14 ≤ l ≤ 14
Reflections collected	19457	5052
Independent reflections	3904 [R_{int} = 0.0384, R_{sigma} = 0.0276]	1934 [R_{int} = 0.0446, R_{sigma} = 0.0509]
Data / restraints / parameters	3904/2/313	1934/1/175
Goodness-of-fit on F^2	1.068	1.034
Final R indexes [I>=2σ(I)]	R_1 = 0.0224 wR_2 = 0.0544	R_1 = 0.0289 wR_2 = 0.0727
Final R indexes [all data]	R_1 = 0.0247 wR_2 = 0.0553	R_1 = 0.0315 wR_2 = 0.0740
Largest diff. peak/ hole / e Å^{-3}	0.94/−1.46	1.16/−1.91

10.0. References

(1) Bardin, M. B.; Ketrush, P. M. Potassium Hexahydroxyiridate(IV). *Russ. J. Inorg. Chem.* **1973**, *18*, 693-696.
(2) Zhang, H.; Chen, B.; Liu, T.; Brudvig, G. W.; Wang, D.; Waegele, M. M. Infrared Spectroscopic Observation of Oxo- and Superoxo-Intermediates in the Water Oxidation Cycle of a Molecular Ir Catalyst. *J. Am. Chem. Soc.* **2024**, *146*, 878-883.
(3) Henderson, W.; McIndoe, J. S. Ionisation techniques. In *Mass Spectrometry of Inorganic and Organometallic Compounds: Tools - Techniques - Tips*, John Wiley & Sons, Ltd, West Sussex, England, 2005; pp 47-106.
(4) DeArmond, M. K.; Hillis, J. E. Phosphorescence of Transition-Metal Chelates. *J. Chem. Phys.* **1968**, *49*, 466-467.
(5) Isakova, V. G.; Baidina, I. A.; Morozova, N. B.; Igumenov, I. K. γ-Halogenated iridium(III) acetylacetonates. *Polyhedron* **2000**, *19*, 1097-1103.
(6) Sato, H.; Taniguchi, T.; Nakahashi, A.; Monde, K.; Yamagishi, A. Effects of Central Metal Ions on Vibrational Circular Dichroism Spectra of Tris-(β-diketonato)metal(III) Complexes. *Inorg. Chem.* **2007**, *46*, 6755-6766.
(7) Wilkie, C. A.; Harworth, D. T. A 13C NMR study of some metal acetylacetone complexes. *J. Inorg. Nucl. Chem.* **1978**, *40*, 195-197.
(8) Sanchis-Perucho, A.; Martínez-Lillo, J. Ferromagnetic exchange interaction in a new Ir(iv)–Cu(ii) chain based on the hexachloroiridate(iv) anion. *Dalton Trans.* **2019**, *48*, 13925-13930.
(9) Parker, S. F.; Williams, K. P. J.; Smith, T.; Ramirez-Cuesta, A. J.; Daemen, L. L. Vibrational Spectroscopy of Hexahalo Complexes. *Inorg. Chem.* **2022**, *61*, 5844-5854.
(10) Hollmann, P.; Preetz, W. Darstellung und Charakterisierung von Dekachlorodiiridat(IV), $[Ir_2Cl_{10}]^{2-}$, und Normalkoordinatenanalyse an Dioktaederkomplexen des Typs $[M_2X_{10}]^{2-}$, M = Re, Os, Ir, Pt; X = Cl, Br / Preparation and Characterization of Decachlorodiiridate(IV), $[Ir_2Cl_{10}]^{2-}$, and Normal Coordinate Analysis of Bioctahedral Complexes $[M_2X_{10}]^{2-}$, M = Re, Os, Ir, Pt; X = Cl, Br. *Z. Naturforsch., B* **1992**, *47*, 1115-1118.
(11) Maltese, M.; Orville-Thomas, W. J. The infrared spectra and structure of some complex hydroxosalts. *J. Inorg. Nucl. Chem.* **1967**, *29*, 2533-2544.
(12) Ravel, B.; Newville, M. ATHENA, ARTEMIS, HEPHAESTUS: data analysis for X-ray absorption spectroscopy using IFEFFIT. *J. Synchrotron Radiat.* **2005**, *12*, 537-541.
(13) Choy, J.-H.; Kim, D.-K.; Hwang, S.-H.; Demazeau, G.; Jung, D.-Y. XANES and EXAFS Studies on the Ir-O Bond Covalency in Ionic Iridium Perovskites. *J. Am. Chem. Soc.* **1995**, *117*, 8557-8566.
(14) Evans, D. F. 400. The determination of the paramagnetic susceptibility of substances in solution by nuclear magnetic resonance. *J. Chem. Soc.* **1959**, 2003-2005.
(15) Stevenson, K. L.; Matson, M. S.; Patrick, R. D. Cis-diaquodioxalatochromate(III) oxalate transfer in aqueous solution. *J. Inorg. Nucl. Chem.* **1971**, *33*, 147-151.
(16) Cosier, J.; Glazer, A. M. A nitrogen-gas-stream cryostat for general X-ray diffraction studies. *J. Appl. Crystallogr.* **1986**, *19*, 105-107.
(17) Sheldrick, G. SHELXT - Integrated space-group and crystal-structure determination. *Acta Crystallogr. A* **2015**, *71*, 3-8.
(18) Sheldrick, G. Crystal structure refinement with SHELXL. *Acta Crystallogr. C.* **2015**, *71*, 3-8.

(19) Dolomanov, O. V.; Bourhis, L. J.; Gildea, R. J.; Howard, J. A. K.; Puschmann, H. OLEX2: a complete structure solution, refinement and analysis program. *J. Appl. Crystallogr.* **2009**, *42*, 339-341.
(20) Piltz, R. Accurate data processing for neutron Laue diffractometers. *J. Appl. Crystallogr.* **2018**, *51*, 635-645.
(21) Piltz, R. LaueG software for displaying and processing neutron Laue images. *J. Appl. Crystallogr.* **2018**, *51*, 963-965.
(22) Betteridge, P. W.; Carruthers, J. R.; Cooper, R. I.; Prout, K.; Watkin, D. J. CRYSTALS version 12: software for guided crystal structure analysis. *J. Appl. Crystallogr.* **2003**, *36*, 1487.
